# Supplementary material for: Skill dependencies uncover nested human capital
Source: Nat Hum Behav. 2025 Feb 24;9(4):673–87. doi: 10.1038/s41562-024-02093-2 (PMC12018457; doi:10.1038/s41562-024-02093-2)
Supplement: Supplementary file 1 — Supplementary Figs. 1–64, Discussion and Tables 1–8. [file 41562_2024_2093_MOESM1_ESM.pdf]

nature human behaviour

Supplementary information https://doi.org/10.1038/s41562-024-02093-2

Skill dependencies uncover nested human capital

In the format provided by the authors and unedited

Contents

1 Skill Groups **1**

2 Skill Nestedness **5**
2.1 Nested Modular structure in Skills and Occupations 5
2.2 Skill's contribution to Nestedness 6
2.3 Nested and Un-nested Skills 7
2.4 Alternative Approach for Deriving Skill Categories 10

3 Conditional Skill Dependencies **12**
3.1 Skills-Occupation Matrix 12
3.2 Skill Dependency from Conditional Probabilities 12
3.3 Linkage to Skill Co-occurrence Networks 18
3.4 Skill Hierarchy Captures Career Progress (Specialization) 20
3.5 Skill Hierarchy Captures Skill Entrapment 21

4 Skill Categories in Career Trajectories **23**
4.1 Resume Data 23
4.2 Occupational Median Age 28
4.3 Individuals' Age and Skills 28

5 Skill Investment and Payoffs **30**
5.1 Investment and Payoffs of Skill Subtypes 30
5.2 Automation Risk and Skills 35
5.3 Skill Payoffs for Different Occupations 35
5.4 Skill Investment and Payoffs in 2005 36

6 Skills' Geographic Distribution **37**
6.1 Counties' Skill Endowments 37
6.2 Skills and Population 38
6.3 Skills and Manufacturing Industries 39

7 Skills' Demographic Distribution **43**
7.1 Parenthood and the Diverging Skills of Male and Female Workers 44
7.2 Gender and Jobs Sorting 45
7.3 Skills and Wage Gaps Have Narrowed Over Years 47
7.4 Gender-Age Divergence of Skills across Demographic Groups 48

8 Historical Patterns of Skill Change for Occupations **50**
8.1 Changes in the Skill Levels 50
8.2 Changes in the Skill Dependency Network 51
8.3 Occupation Taxonomy 52

9 Robustness Checks with Management and Admin Occupations and Social Skills **54**
9.1 Role of Management and Administrative Occupations 54
9.2 Role of Social Skills 59

Introduction to the Supplementary Information

Section 1 offers details on the statistical derivations and robustness checks corresponding to the results on Generality in the main text (Figs. 1-4), and the resulting skill groups (Tab. 1) introduced in the main Fig. 1 (a-c) and used across the paper.

Section 2 expands on the nestedness of occupation-skill networks in part shown in Fig. 3 and used throughout the paper. It describes the rationale for (Fig. 5 and 6), the methodology of measuring skill-level contribution to nestedness, alternative measurement of skills' contributions, the results based on different measures (Fig. 7-9), and the resulting split of skills based on nestedness we used throughout the paper (Tab. 2). It also includes an alternative approach to splitting skills based on correlation, which yields consistent results(Tab. 3).

Section 3 articulates the construction of the skill hierarchy of the main Fig. 2 (a and b). It describes how we derive conditional probabilities between pairs of skills (which is briefly introduced in Fig. 1 (d), the choice of parameters (Figs. 11-13), and sensitivity analysis (Figs. 14). Figs. 15 and 16 show the full and backboned skill hierarchy network with all skill labels attached. The section highlights the linkage between our skill hierarchy and a skill co-occurrence network (Fig. 17), and offers two cases based on comparing registered nurses with nurse practitioners (Fig. 18), and the skill entrapment of some immigrants (Figs. 19-20), to showcase how the skill hierarchy captures career progress.

Section 4 expands on the temporal analyses reported in the main Fig. 4. We have explicated the preparation process (Figs. 21-23 and Tab. 4) Bootstrapping of the job sequences in resume data (Fig. 25-26), and included the result for all skill cateogires (Fig. 24). We also include details about the analysis of median age of workers (Fig. 27), and the analysis of synthetic birth cohorts based on CPS (Fig. 28). As robustness checks, we also show that the skill development observed in the main Fig. 4 continues long after education (Fig. 29) and also emerges for individuals without college education (Fig. 30).

Section 5 expands the analyses of the main Fig. 5, capturing the correlation of occupational wages, educational requirement, and experience with their average levels of each skill category (Fig. 32-34), supplements these results by robustness checks, using alternative measures of skill levels (Fig. 35-37), and regression analyses (Tab. 5). We show the robustness of the main wage findings across major occupational groups (Fig. 39), replicate the main Fig. 5 based on the data of year 2005 (Fig. 40), and finally show the correlation between levels of each skill categories and occupational automation risk [1] (Fig. 38).

Section 6 offers a descriptive geographic analysis of skill distribution. We offer evidence that part of the urban wage premiums is explained by the distribution of general and nested skills (Tab. 8, and Figs. 41-47), but leave an in-depth study of the topic for future work.

Section 7 extends the demographic skill analysis of the main Fig. 6, in Fig. 48. Figs. 51 and 52 capture temporal patterns of these factors, exhibiting the gaps have narrowed over time. Fig. 53 depicts that racial/ethnic and gender differentials in skills follow similar age trends observed in the main Fig. 4, and are robust to time-variant economic factors (Fig. 54). In Fig. 50, we highlight the differential influence of parenthood on male and female workers, observed in the diverging growth of general and nested skills in the main Fig. 4.

Section 8 expands on the changes in occupational skill requirements between 2005 and 2019 (the main Fig. 7), and the resulting changes in the skill hierarchy (Figs. 55-57). The section also provides a brief discussion of the changes in the occupational taxonomy (Fig. 58).

Section 9 offers a battery of robustness checks on whether administrative and managerial occupations (Figs. 59-63 and Tab. 10) or social skills (Figs. 64) derive the increasingly important role of general skills.

1 Skill Groups

We obtain data-driven categories of skill generality by grouping skills based on their Level Distributions. We employ a k-means clustering algorithm (see Fig. 1 in the main) and supplement the results with two more measures of skill generality (the average skill level and occupation counts), explained later in this and the next sections. Here, we discuss clustering skills based on their distribution shapes, as Fig. 1 shows in the main text.

The k-means clustering algorithm requires two inputs, a distance metric and the number of clusters, k. We choose the correlation distance (as in equation 1) for the former and $k = 3$ for the latter. We did not use Euclidean distance because it does not differentiate the shape distributions as inputs compared to correlation. Second, we choose $k = 3$ because it seems to be in the range of optimal numbers (2-4) from various statistical tests shown in Fig. 1. Finally, we provide two alternative categories of skill generality, which are consistent with the results of the k-means clustering.

To measure the correlation similarity among the distribution shapes, we binned the distribution with intervals of 0.1. For instance, the skill level ranges from 0 to 7, resulting in a vector of 35 entries, each corresponding to bins of [0,0.10), [0.10, 0.20),... Correlation similarities are measured across these vectors. Table S1 shows the assignment of skills resulting from k-means clustering (based on correlation similarity and $k = 3$) used in the main text.

$$d = 1 - \frac{\Sigma_i x_i y_i - \frac{1}{n}\Sigma_i x_i \Sigma_i y_i}{\sqrt{\Sigma_i x_i^2 - \frac{1}{n}(\Sigma_i x_i)^2}\sqrt{\Sigma_i y_i^2 - \frac{1}{n}(\Sigma_i y_i)^2}} \quad (1)$$

We use three statistical tests to determine the optimal k. These include the elbow method, silhouette analysis, and gap statistics, as shown in Fig. 1. These results suggest optimal numbers from 2 to 4. We provide the clusters resulting from each choice of k in Figs. 2-4.

The conventional *Elbow method* calculates the within-cluster sum of squares for different numbers of clusters k in order to find a sharp decline from one k to another followed by a more gradual decrease in slope, where we find $k = 3$ is the best. *Silhouette analysis* [2] measures the similarity of each observation with the cluster to which it is assigned, producing a metric that ranges from -1 (dissimilar) to 1 (similar). In determining the optimal k, one looks for the value at which the average (silhouette width) is maximum, providing $k = 2$ for the optimal number. The *Gap statistic* [3] compares the total intracluster variation for different k with their expected values under a null model (i.e., a distribution with no obvious clustering, generated using (1,000 iterations of) Monte Carlo simulations of the sampling process,) wherein maximal intracluster variation is desired, providing $k = 4$ for the optimal number. To determine the optimal number of clusters, k, based on Gap Statistic, we used the criterion proposed by [3], wherein the smallest k such that the *change* in intracluster variation, f, is smaller than an (error-adjusted) standard deviation, s of the null model ($f(k+1) - f(k) \geq s_{k+1}$).

Figures 2-4 show individual skills within categories. The number of groups does not change the order of generality of skills, which is central to our analysis. The context of our study encourages a focus on the most and the least general skills because those epitomize two skill categories of broad theoretical interest: general skills and specialized skills. Therefore, it is a practical choice for us to start with three clusters, focus primarily on the two extremes, and subject the skills in the remaining cluster to secondary examination. Given the visual shapes of distributions and the semantic benefit of differentiating the most general and moderately general skills (so-called intermediate skills), we continue using $k = 3$ in the main text. To reduce any inherent noise due to the skills between general and specifics, we choose $k = 3$ and

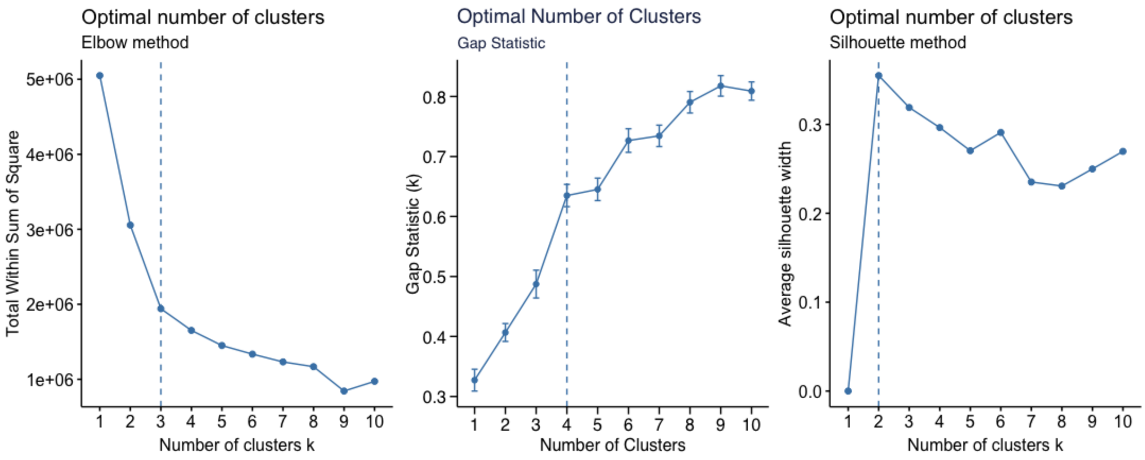

Supplementary Figure 1: Statistical tests to determine the optimal k for k-mean clustering algorithms. The figure shows the results of Elbow method, Gap statistic ($n_{MonteCarloiterations} = 1,000$, standard error as error bars), and Silhouette analysis.

focus on general and specifics. Table 1 shows the resulting split and offers some supporting statistics.

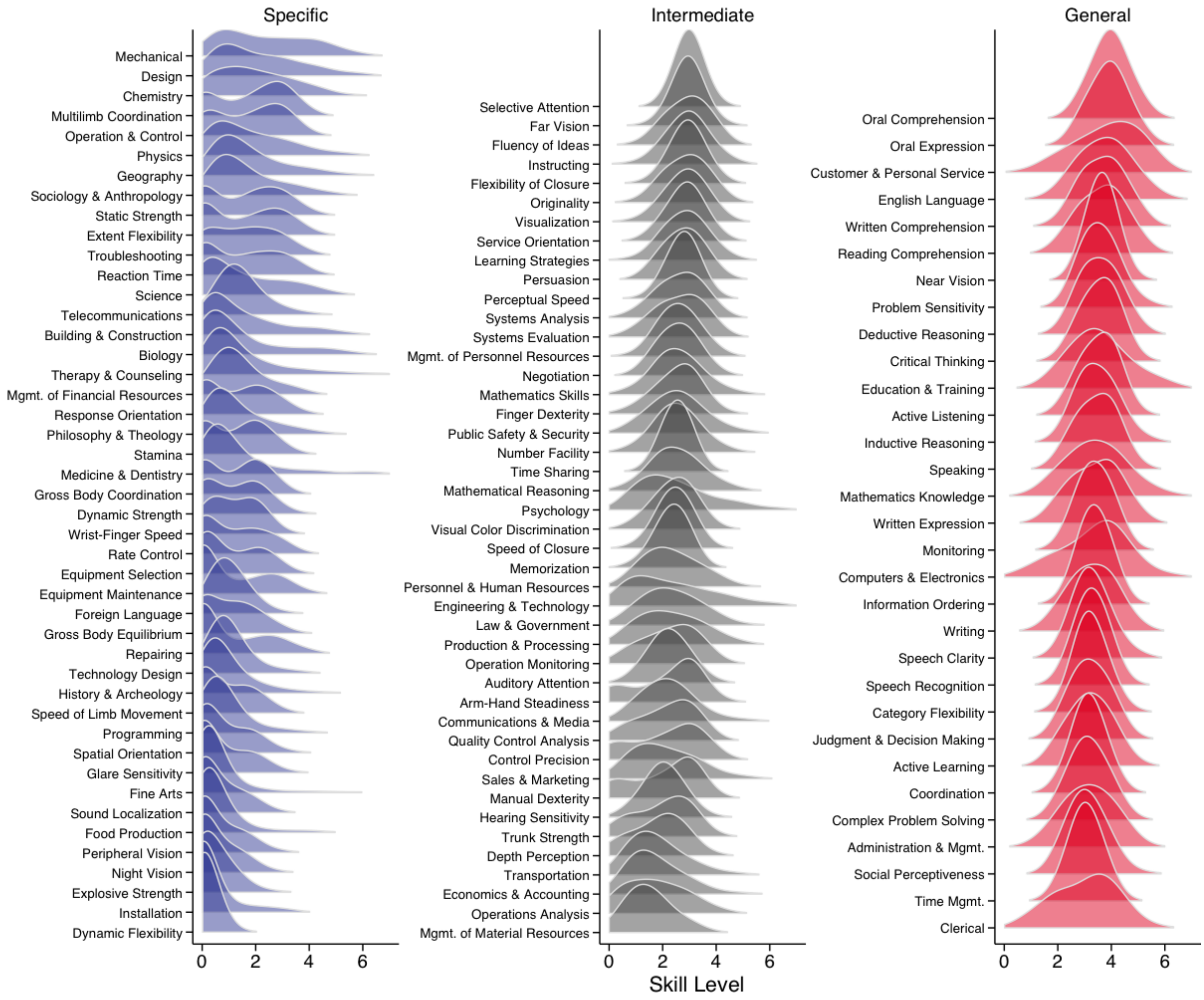

Supplementary Figure 2: Skill Level Distribution with $k = 3$. Skills are in descending order of generality. The depicted distribution of skills is used in the main text.

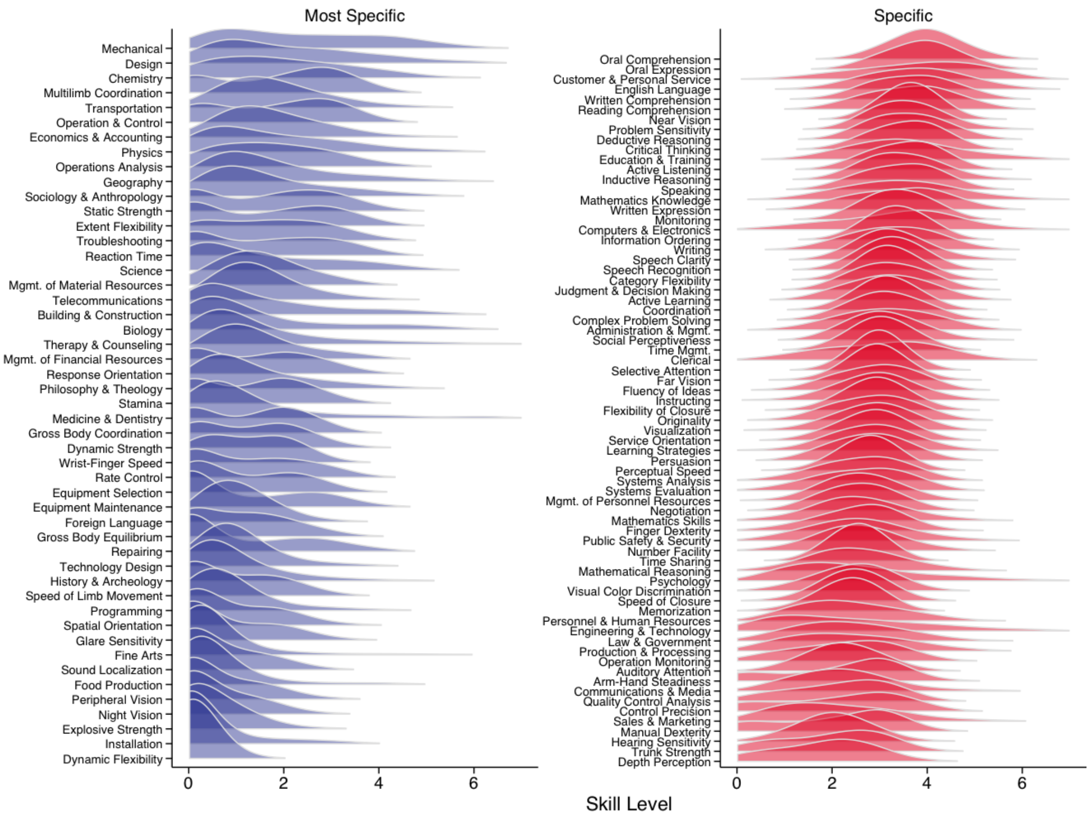

Supplementary Figure 3: Skill Level Distribution with $k = 2$. Skills are in descending order of generality.

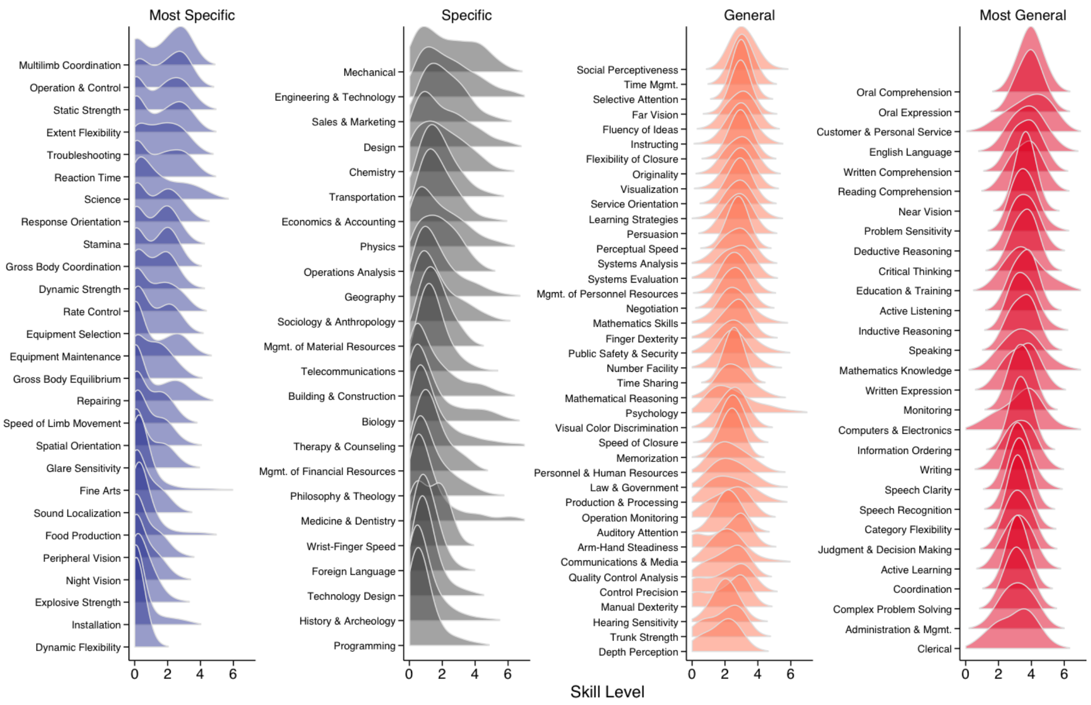

Supplementary Figure 4: Skill Level Distribution with $k = 4$. Skills are in descending order of generality.

Supplementary Table 1: The Skill Group Assignment Resulting from Clustering Skills based on their Usage Distribution Shapes.. Skills are ordered by their generality (their average levels demanded across occupations, and their marginal totals in the binary skill-occupation network).

Skill Group	Skill Titles (skill generality, occupation counts)
General (3.43, 563.61)	Oral Comprehension (3.96, 682), Oral Expression (3.9, 680), Customer and Personal Service (3.85, 604), English Language (3.76, 590), Written Comprehension (3.68, 598), Reading Comprehension (3.65, 593), Near Vision (3.64, 703), Problem Sensitivity (3.58, 571), Deductive Reasoning (3.58, 553), Critical Thinking (3.57, 594), Education and Training (3.55, 539), Active Listening (3.54, 593), Inductive Reasoning (3.48, 533), Speaking (3.45, 550), Mathematics Knowledge (3.43, 538), Written Expression (3.4, 532), Monitoring (3.38, 589), Computers and Electronics (3.38, 582), Information Ordering (3.37, 616), Writing (3.29, 514), Speech Clarity (3.28, 474), Speech Recognition (3.26, 589), Category Flexibility (3.23, 610), Judgment and Decision Making (3.21, 520), Active Learning (3.19, 531), Coordination (3.16, 534), Complex Problem Solving (3.15, 498), Administration and Management (3.15, 507), Social Perceptiveness (3.1, 486), Time Management (3.05, 477), Clerical (3.03, 492)
Intermediate (2.44, 281.42)	Selective Attention (2.99, 496), Fluency of Ideas (2.94, 451), Far Vision (2.94, 451), Flexibility of Closure (2.92, 420), Instructing (2.92, 435), Originality (2.88, 440), Visualization (2.86, 415), Service Orientation (2.85, 442), Learning Strategies (2.84, 387), Persuasion (2.79, 366), Perceptual Speed (2.76, 317), Systems Analysis (2.64, 321), Systems Evaluation (2.63, 337), Management of Personnel Resources (2.6, 245), Mathematics Skills (2.57, 284), Negotiation (2.57, 277), Finger Dexterity (2.56, 352), Public Safety and Security (2.55, 298), Number Facility (2.54, 264), Time Sharing (2.54, 164), Mathematical Reasoning (2.52, 267), Psychology (2.47, 305), Visual Color Discrimination (2.45, 207), Speed of Closure (2.44, 128), Memorization (2.34, 120), Personnel and Human Resources (2.33, 234), Engineering and Technology (2.33, 296), Law and Government (2.32, 241), Production and Processing (2.29, 302), Operation Monitoring (2.28, 250), Auditory Attention (2.23, 175), Communications and Media (2.21, 200), Arm-Hand Steadiness (2.21, 327), Quality Control Analysis (2.19, 216), Control Precision (2.17, 318), Sales and Marketing (2.09, 230), Manual Dexterity (2.08, 302), Hearing Sensitivity (2.04, 85), Trunk Strength (2.01, 238), Depth Perception (1.84, 122), Transportation (1.79, 119), Economics and Accounting (1.77, 129), Operations Analysis (1.75, 128)
Specific (1.22, 93.3)	Mechanical (2.4, 354), Design (2.08, 244), Chemistry (1.97, 204), Multilimb Coordination (1.88, 264), Operation and Control (1.79, 192), Physics (1.76, 147), Geography (1.71, 159), Sociology and Anthropology (1.65, 153), Static Strength (1.64, 211), Extent Flexibility (1.59, 234), Troubleshooting (1.58, 126), Reaction Time (1.53, 189), Science (1.52, 154), Management of Material Resources (1.5, 32), Telecommunications (1.48, 53), Building and Construction (1.47, 137), Biology (1.42, 152), Therapy and Counseling (1.42, 119), Management of Financial Resources (1.37, 69), Response Orientation (1.32, 72), Philosophy and Theology (1.31, 102), Medicine and Dentistry (1.26, 108), Stamina (1.26, 79), Gross Body Coordination (1.24, 55), Dynamic Strength (1.21, 56), Wrist-Finger Speed (1.2, 21), Rate Control (1.18, 93), Equipment Selection (1.09, 15), Equipment Maintenance (1.06, 95), Foreign Language (1.03, 17), Gross Body Equilibrium (1.03, 33), Technology Design (1.02, 19), Repairing (1.02, 91), History and Archeology (0.93, 52), Speed of Limb Movement (0.86, 11), Programming (0.84, 29), Spatial Orientation (0.83, 21), Glare Sensitivity (0.74, 13), Fine Arts (0.6, 43), Sound Localization (0.6, 3), Peripheral Vision (0.59, 8), Food Production (0.59, 44), Night Vision (0.53, 2), Explosive Strength (0.48, 4), Installation (0.37, 11), Dynamic Flexibility (0.15, 2)

2 Skill Nestedness

2.1 Nested Modular structure in Skills and Occupations

As the scope of knowledge expands, the need for specialization grows. Unlike findings of the economic complexity about the nested landscape of national, regional, and urban capabilities [4, 5], occupations often bundle few skills and therefore encompass much narrower knowledge domains. While the main focus of our paper is revealing the underlying structure of workplace skills, noting the distinction is vital. Here, we empirically offer evidence of the difference between occupation and skill scopes in two ways before discussing nestedness in the skill space. We find higher variation among skills (in the number of occupations that demand a skill) than among occupations (in the number of skills an occupation demands), as seen in Fig. 5), suggesting a non-trivial nested structure (Fig. 6).

Figure 5 shows the Level Distribution of skills (red) and the distribution of the total skill amounts in occupations (blue). To obtain the skill Level Distribution, one measures the demand for each skill and makes a distribution. For example, how much English skills are needed for the entire labor market or how much Physics skills are needed across occupations. The former is more broadly used (i.e., general) and therefore has a higher demand than the latter. A skill's demand is calculated by summing skill levels/importances in the occupations (red). Similarly, by adding the total levels/importance of each occupation, one obtains occupations' skill endowments, the total level of skills needed to undertake the job's tasks.

Figure 5 shows, unlike broad skill generality, occupation's endowment is narrowly distributed. This narrow distribution indicates that the total amount of skills needed for an occupation is not much different from each other, regardless of how much they are paid and how advanced education is needed. We attribute occupations' limited scope of skills to the limited scope or attention that individual workers can offer. There is only so much a single person can equip and do. Thus, individuals' capacity restricts how many skills occupations can bundle. This constraint explains the process of specializations needed for a complex job. In contrast to occupations, skills do not have such constraints. While some skills are niche, general skills epitomize expertise of widespread demand, as they are needed in most occupations.

This stark difference in the scope of occupations and skills requires quantifying the nestedness structure of the skill-occupation matrix differently. In ecological terms, there is no site/area/biome (occupation) that is large enough to nest other sites (occupations), whereas there are species (skills) that can nest other species (skills) as they can appear anywhere. This explains why Fig. 6 shows the noisy nested structure in the skill-occupation matrix, far from the perfect nested triangle. The skill-occupation structure allows mutually exclusive presences, possibly due to competition between skills within an occupation.

We construct and measure a nested structure of a skill-occupation matrix in Fig. 6. The original skill-occupation matrix's entry is a continuous variable (indication of the degree or point along a continuum to which a particular descriptor is required or needed to perform the occupation). But most conventional nestedness analyses require binary entries, and thus, we employ a disparity filter to make the matrix binary entries of statistically significant presences (see Sec. 3.1). We then sort the matrix entries in descending orders of marginal totals [6]. As Fig. 6 shows, the result deviates from the perfect nested structure as an upper-left triangle. Nevertheless, the upper left is highly populated, indicating a nested structure. This imperfect nested structure may account for the constraints on occupations (limited carrying capacity), introducing severe competition between skill species. This constraint distinguishes the nestedness of extensive economies of nations, regions, and urban areas from occupation's nestedness,

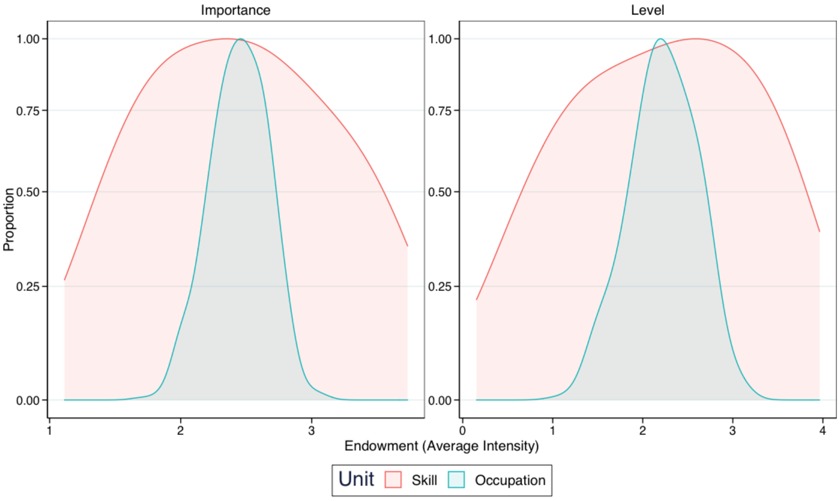

Supplementary Figure 5: Scaled Density Distribution of Skill and Occupation Endowments. Endowment for skills and occupations is calculated by averaging the intensity values for each column and row, respectively. We compute endowment using both intensity measures of Importance and Level. The results contrast for endowment distribution of skills from occupations. Unlike skills, occupations show closer average Importance values. This finding implies occupation's attention is constrained. Hence, they must allocate their limited attention to skills.

for which specializations dominate the evolution more than diversification. As a result, the skill-occupation matrix is expected to be modular as well as nested with mutually exclusive modules. *Nested-modular matrix* is a complicated structure and will be beyond our current scope [7]. Here, we will focus on individual skills' contributions to the nested structure and differentiates skills that contribute to the nested structure from those that do not.

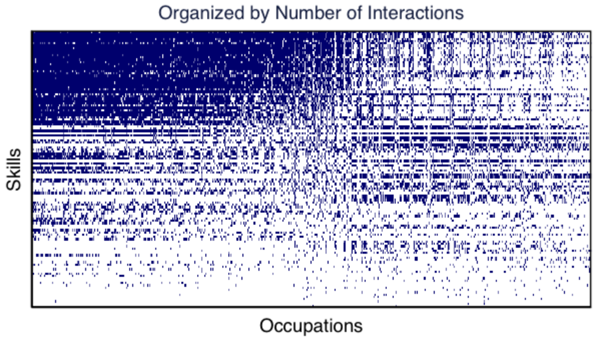

Supplementary Figure 6: The skill-occupation matrix The occupations and skills of the matrix are arranged in descending order of their marginal fills (along the x and y axes, respectively.)

2.2 Skill's contribution to Nestedness

The above evidence reveals that the landscape of human capital is partially nested due to matching increasing complexity with specialization (perhaps, niche constructions), resulting in a nested-modular structure. We speculate that increasing complexity mainly generates nested structure, and specialization mainly generates modular structure. We think mathematical modeling of a labor ecosystem according to this insight can be extremely interesting, and we leave this for future work while we focus on empirical observations and quantifications for now.

Due to the structural complications, described above, conventional approaches for quantifying nested structure (sorting the matrix to observe an upper triangle or calculating pres-

ences/absences accounting for a well-defined nested structure) are likely imperfect. Instead, we look for skills' individual contributions to the current nested structure compared to their counterfactual contributions under a null model. For instance, [8] proposes such an approach based on the idea of randomizing edges for a focal skill and comparing the nestedness in the simulated network with the observed value in the system. In our case, we create counterfactual worlds as if a focal skill can appear equally likely in any occupation. This equally probable null hypothesis randomly chooses a focal skill's occupations (edges) without considering education, domain knowledge, industrial requirements, or historical contingency, imposing the current socio-economic structure. Then, we measure an increase/decrease in nestedness by destroying the current imposition. For simplicity, this method is only available for a presence/absence bipartite network [9, 10]. We use the disparity filter [11] because the method preserves degree heterogeneity, which is crucial to distinguishing general from niche skills. We explain this method in more detail in the supplementary section 3.

We use three commonly used metrics of nestedness (checkerboard score, Temperature, and NODF) to quantify nestedness N at the level of the skill-occupation matrix. Checkerboard score measures the deviation from nestedness as checkerboard appearance of fills [12]. This score is consistent with the well-known nestedness index, N_c, counting the number of times that a species' presence at a site correctly predicts its presence at richer sites and sums these counts across species and sites [13]. The presence of a checkerboard, an empty site when the nested site predicts the fill, decreases the nestedness. Temperature measures as the total number of "surprises" on the assumption of a perfectly nested matrix as temperature increases thermal noises to destroy perfect structure [14]. Although this is a great measure, this index has its underlying assumption that the system is actually following the mechanism for a perfect nested structure if there is no temperature. NODF quantifies nested overlaps, the notion that all species in a poor habitat are present in richer habitats, and decreasing fill (marginal totals of interactions between habitats and species) [10].

Now that we identify the null hypothesis to generate a focal skill's counterfactuals and nestedness indexes let's calculate skills' contributions to nestedness, c_s. For each skill, we run 5,000 simulations, wherein, the focal skills' ties to occupations are randomly shuffled, keeping the number of ties constant. Therefore, all ties of the focal skill (meaning the skill's generality) are preserved. Then, we measured a nestedness index of the generated matrix mentioned above, as N^*. We quantify a skill s's contribution as:

$$c_s = \frac{N - < N_s^* >}{\sigma_{N_s^*}} \tag{2}$$

$< N_s^* >$ and $\sigma_{N_s^*}$ denote the mean and standard deviation of the nestedness of the simulated matrix, in which skill s's edges were randomized.

2.3 Nested and Un-nested Skills

Supplementary Figs. 7, 8, and 9 show the nestedness contribution of skills using checkerboard score, Temperature, and NODF, respectively. We would like to compare skills at the same generality level to avoid comparing apples to oranges. For example, it is not fair to compare general skills to specific skills as they have more edges. Given that general skills mass at the root of those dense dependency webs, we show more closely examine the nestedness contribution of intermediate and specific skills, and use the measurement to split them into categories of nested and un-nested.

Table 2 shows the resulting split of skills into categories or subtypes based on the checker-

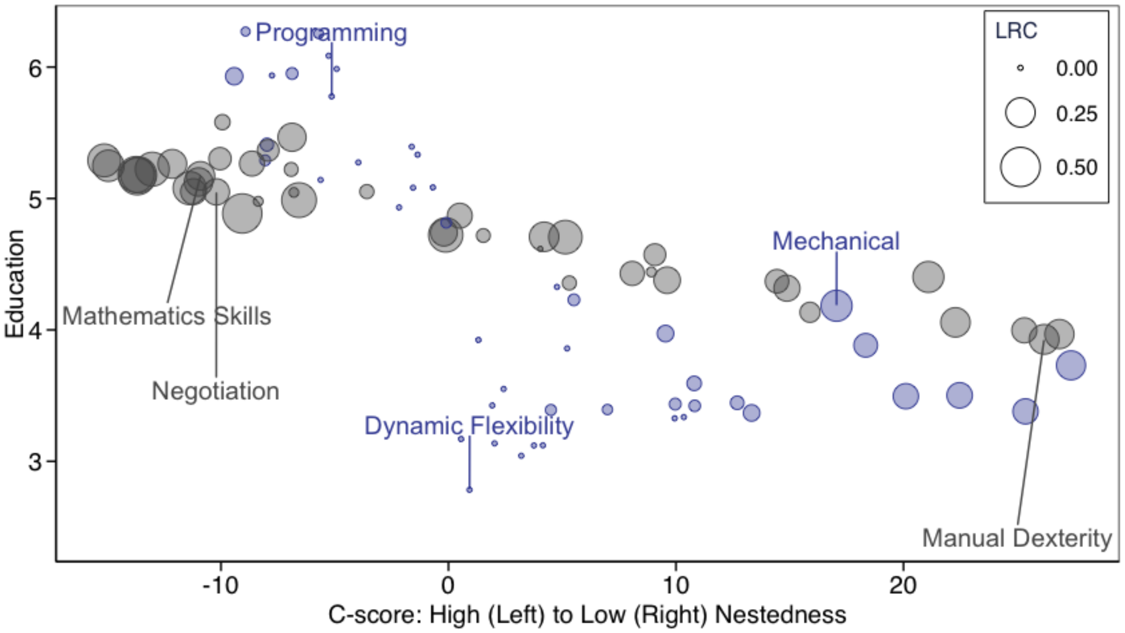

Supplementary Figure 7: Nestedness Contribution of Skills based on checkerboard [12].

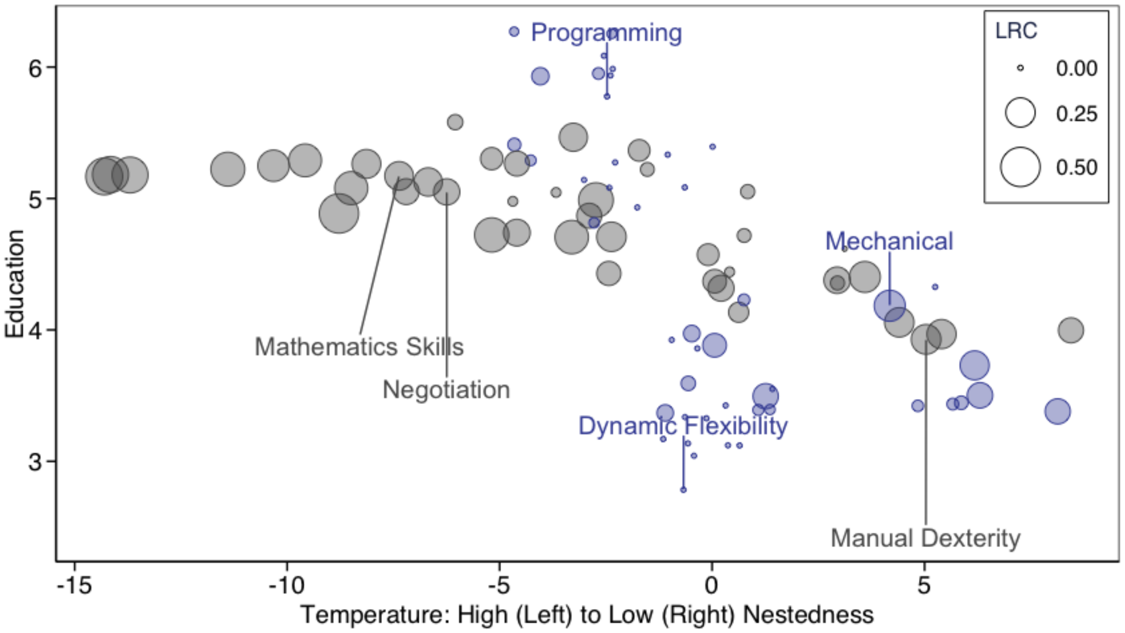

Supplementary Figure 8: Nestedness Contribution of Skills based on Temperature [14].

board score [12] also shown in Fig. 7. To be clear, we refer to the result of our skill clustering based on generality *skill clusters* (general, intermediate, and specific) and refer to the further split made based on nestedness *skill categories* or *skill subtypes* (general, nested intermediate, nested specific, un-nested intermediate, un-nested specific.). Finally, Fig. 10 plots education against nestedness score based on N_c, which is used in the main text.

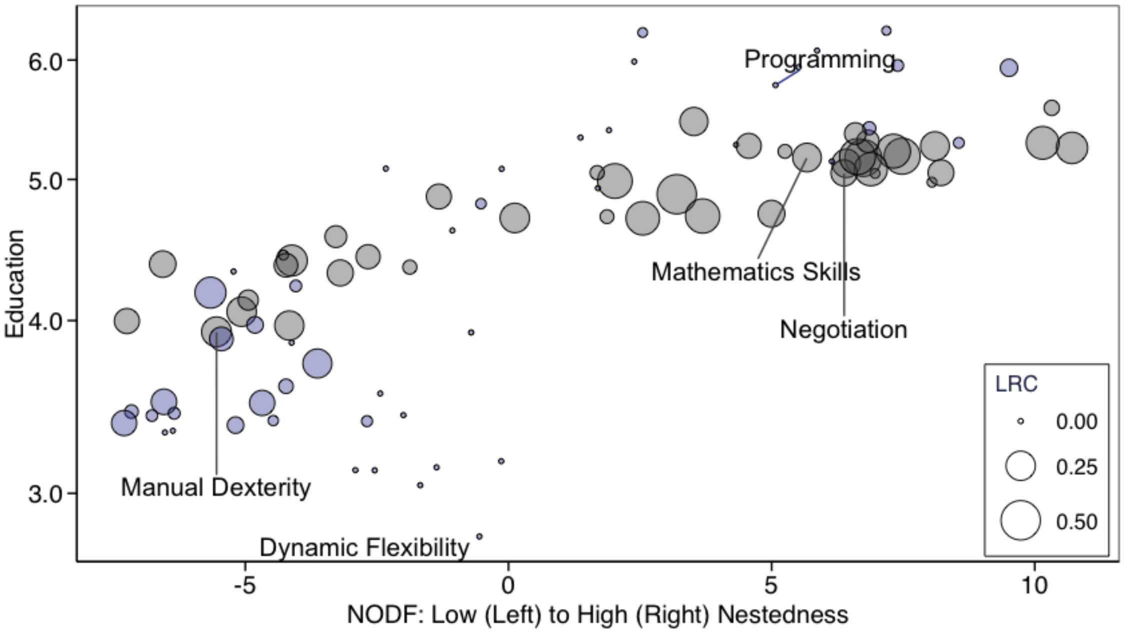

Supplementary Figure 9: Nestedness Contribution of Skills based on NODF[10]. Extreme values arise from large denominators for some of the skills.

Supplementary Table 2: The Skill Split Resulting from Nested Contribution of Skills.

Skill Category	Skill Titles
Nested Specific (18 skills)	Biology, Chemistry, Design, Fine Arts, Foreign Language, Geography, History and Archeology, Management of Financial Resources, Management of Material Resources, Medicine and Dentistry, Philosophy and Theology, Physics, Programming, Science, Sociology and Anthropology, Technology Design, Telecommunications, Therapy and Counseling
Un-nested Specific (28 skills)	Building and Construction, Dynamic Flexibility, Dynamic Strength, Equipment Maintenance, Equipment Selection, Explosive Strength, Extent Flexibility, Food Production, Glare Sensitivity, Gross Body Coordination, Gross Body Equilibrium, Installation, Mechanical, Multilimb Coordination, Night Vision, Operation and Control, Peripheral Vision, Rate Control, Reaction Time, Repairing, Response Orientation, Sound Localization, Spatial Orientation, Speed of Limb Movement, Stamina, Static Strength, Troubleshooting, Wrist-Finger Speed
Nested Intermediate (25 skills)	Communications and Media, Economics and Accounting, Far Vision, Flexibility of Closure, Fluency of Ideas, Instructing, Law and Government, Learning Strategies, Management of Personnel Resources, Mathematical Reasoning, Mathematics Skills, Memorization, Negotiation, Number Facility, Operations Analysis, Originality, Personnel and Human Resources, Persuasion, Psychology, Sales and Marketing, Selective Attention, Service Orientation, Speed of Closure, Systems Analysis, Systems Evaluation
Un-nested Intermediate (18 skills)	Arm-Hand Steadiness, Auditory Attention, Control Precision, Depth Perception, Engineering and Technology, Finger Dexterity, Hearing Sensitivity, Manual Dexterity, Operation Monitoring, Perceptual Speed, Production and Processing, Public Safety and Security, Quality Control Analysis, Time Sharing, Transportation, Trunk Strength, Visual Color Discrimination, Visualization

Supplementary Table 2: The Skill Split Resulting from Nested Contribution of Skills.

Skill Category	Skill Titles
General (31 skills)	Active Learning, Active Listening, Administration and Management, Category Flexibility, Clerical, Complex Problem Solving, Computers and Electronics, Coordination, Critical Thinking, Customer and Personal Service, Deductive Reasoning, Education and Training, English Language, Inductive Reasoning, Information Ordering, Judgment and Decision Making, Mathematics Knowledge, Monitoring, Near Vision, Oral Comprehension, Oral Expression, Problem Sensitivity, Reading Comprehension, Social Perceptiveness, Speaking, Speech Clarity, Speech Recognition, Time Management, Writing, Written Comprehension, Written Expression

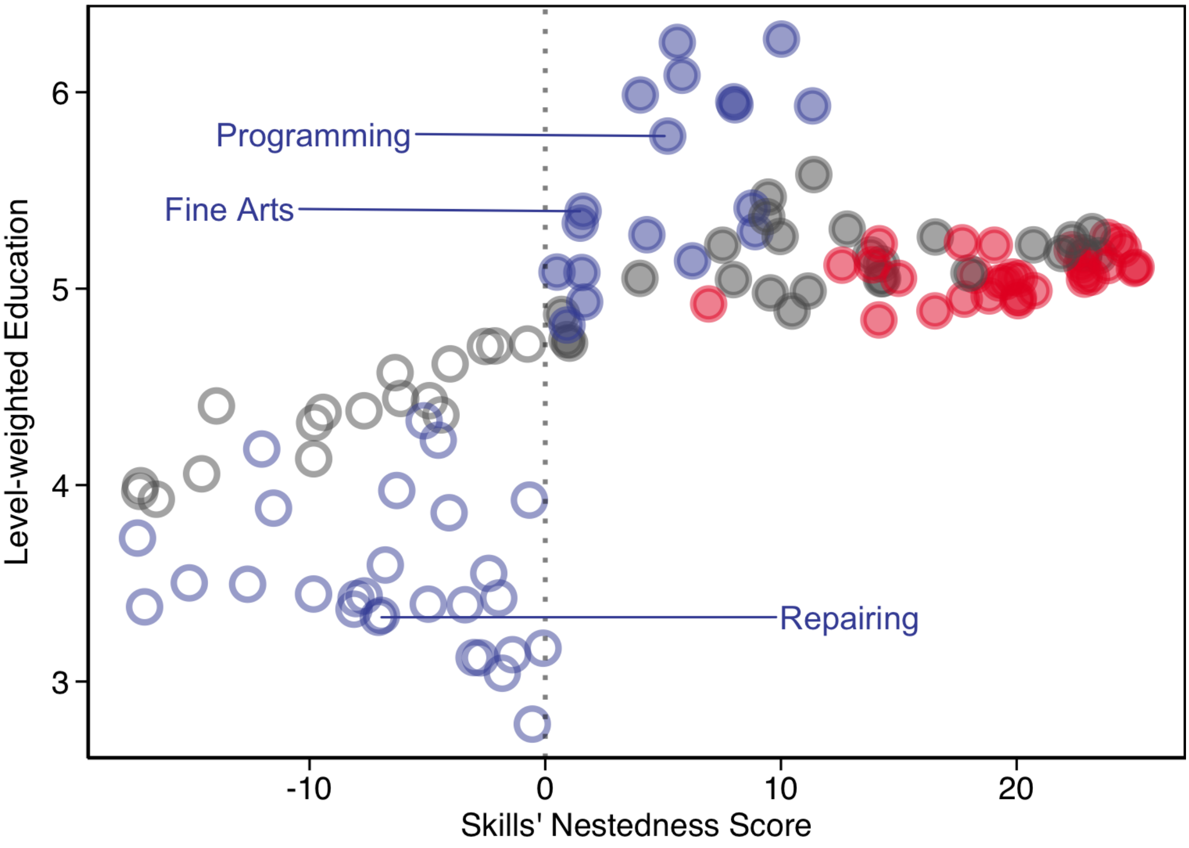

Supplementary Figure 10: Education vs. Nestedness Contribution of Skills based the overlap index, N_c.

2.4 Alternative Approach for Deriving Skill Categories

We split the skills of each cluster (general, intermediate, and specific) based on their correspondence with general skills. We measure such correspondence C by calculating the correlation between the importance of given skill i, and the importance of each of the general skills j:

$$C_{i,j\in<\text{general}>}^{<Level>} = \frac{\Sigma_o(Level_{i,o} - \mu_{Level_i})(Level_{j,o} - \mu_{Level_j})}{\sqrt{\Sigma_o(Level_{i,o} - \mu_{Level_i})^2 \Sigma_o(Level_{j,o} - \mu_{Level_j})^2}} \quad (3)$$

Aggregating values of $C_{i,j}$ over general skills j, we obtain a measure of correspondence between skill i and the set of general skills, $C_{i,<general>}$. Then, we compare skill i to other skills l in the same cluster k to which i belongs— given our assignment from supplementary section 1. To do so, for skills l of cluster $k \in \{\text{intermediate}, \text{specific}\}$, we calculate the mean correlation to general skills:

$$C_k^{<mean>} = mean_{l \in k} C_{i,<general>} \tag{4}$$

Finally, we suggest a skill i of cluster $k \in Specific, Intermediate$ is nested if it depends on general skills above the mean level and call it '*nested*' if $C_{i,<general>} \geq C_k^{<mean>}$, and suggest it is independent of general skills and call it '*un-nested*', otherwise. Table 3 shows the resulting assignment of skills based on this approach. One obtains a similar split of skills if the Importance measure instead of Level is used.

Supplementary Table 3: The Skill Split Resulting from Correlation Dependence Analysis described above.

Skill Category	Skill Titles
Nested Specific (20 skills)	Biology, Building and Construction, Chemistry, Design, Fine Arts, Food Production, Foreign Language, Geography, History and Archeology, Management of Financial Resources, Management of Material Resources, Medicine and Dentistry, Philosophy and Theology, Physics, Programming, Science, Sociology and Anthropology, Technology Design, Telecommunications, Therapy and Counseling
Un-nested Specific (26 skills)	Dynamic Flexibility, Dynamic Strength, Equipment Maintenance, Equipment Selection, Explosive Strength, Extent Flexibility, Glare Sensitivity, Gross Body Coordination, Gross Body Equilibrium, Installation, Mechanical, Multilimb Coordination, Night Vision, Operation and Control, Peripheral Vision, Rate Control, Reaction Time, Repairing, Response Orientation, Sound Localization, Spatial Orientation, Speed of Limb Movement, Stamina, Static Strength, Troubleshooting, Wrist-Finger Speed
Nested Intermediate (23 skills)	Communications and Media, Economics and Accounting, Flexibility of Closure, Fluency of Ideas, Instructing, Law and Government, Learning Strategies, Management of Personnel Resources, Mathematical Reasoning, Mathematics Skills, Memorization, Negotiation, Number Facility, Operations Analysis, Originality, Personnel and Human Resources, Persuasion, Psychology, Sales and Marketing, Service Orientation, Speed of Closure, Systems Analysis, Systems Evaluation
Un-nested Intermediate (20 skills)	Arm-Hand Steadiness, Auditory Attention, Control Precision, Depth Perception, Engineering and Technology, Far Vision, Finger Dexterity, Hearing Sensitivity, Manual Dexterity, Operation Monitoring, Perceptual Speed, Production and Processing, Public Safety and Security, Quality Control Analysis, Selective Attention, Time Sharing, Transportation, Trunk Strength, Visual Color Discrimination, Visualization
General (31 skills)	Active Learning, Active Listening, Administration and Management, Category Flexibility, Clerical, Complex Problem Solving, Computers and Electronics, Coordination, Critical Thinking, Customer and Personal Service, Deductive Reasoning, Education and Training, English Language, Inductive Reasoning, Information Ordering, Judgment and Decision Making, Mathematics Knowledge, Monitoring, Near Vision, Oral Comprehension, Oral Expression, Problem Sensitivity, Reading Comprehension, Social Perceptiveness, Speaking, Speech Clarity, Speech Recognition, Time Management, Writing, Written Comprehension, Written Expression

3 Conditional Skill Dependencies

To obtain the skill structure, as seen in main Fig. 2, we extract conditional probabilities of the appearance of a skill u, given the appearance of another, v, in the skill-occupation matrix, which was used for nested structure in the previous section [15].

3.1 Skills-Occupation Matrix

The original skill-occupation matrix's entry is a continuous variable (indication of the degree, or point along a continuum, to which a particular descriptor is required or needed to perform the occupation). But most conventional nestedness analyses, used in section 2, and conditional probability measures for main Fig. 2, require binary entries. Thus, we employ a disparity filter to make the matrix binary entries of statistically significant presences [11].

We chose this method for two reasons. First, it allows the user to set different restrictions on the skill and occupation sides of the bipartite network. This feature is desirable given the differences in the strength and degree distributions of occupations and skills. Second, it accommodates heterogeneous degree distribution, which we know is a key characteristic of our skill side. In choosing the parameters, we ensured the resulting binary network satisfied the following conditions. First, the filtered network has to remain faithful to the skill and occupation degree distributions (macro-level features). We show that the filter indeed kept the distribution shapes in Fig. 11 for the skills strength distribution and Fig. 12 for occupations' strength correlations. The Pearson correlation between skills' strengths (sum of edge weights) and their transformed degree is 0.95. The ranking of skills across these two measures is also preserved (correlation is 0.97). Note that we used comparisons between node strengths and node degrees because of our idiosyncratic empirical data structure. Each occupation includes a survey for every skill, resulting in every occupation having every skill entry with numbers ranging from 0 to 7. The surviving skills preserve the distribution and ranking of occupations (correlations between occupations' strength and ranking before and after transformation are 0.79, and 0.79, respectively).

In the end, the parameter pair ($\alpha_{in} = 0.4$, $\alpha_{out} = 0.275$) results in 33,865 (29%) edges. We also conducted validity checks on the choice of parameters by examining the sampled results (5% of occupations). The test compares the survived and eliminated skills to common sense. For example, have the links between "Surgeon" and the skill "Medicine and Dentistry", and "Programmer" and skill "Programming" survived? Conversely, has the link between "Mathematician" and "Explosive Strength", defined as *The ability to use short bursts of muscle force to propel oneself (as in jumping or sprinting), or to throw an object*, been eliminated? The goal of this exercise is to ensure the parameters are not set too strictly or too lenient, and that the retained information in ties conforms to expectations.

3.2 Skill Dependency from Conditional Probabilities

We first account for the significant conditional appearances, and discount for noise from independent co-appearances (of two skills in occupation) by random chance with a z-score threshold, z_{th} [15]. That is, we account only for those skills that appear together more than randomly expected by z_{th} magnitude. Here, z_{th} is a threshold for the extent to which we eliminate chance from two skills appearing in the same occupation.

$$z_{u,v} = \frac{N(u,v) - \mu}{\sigma} > z_{th} \tag{5}$$

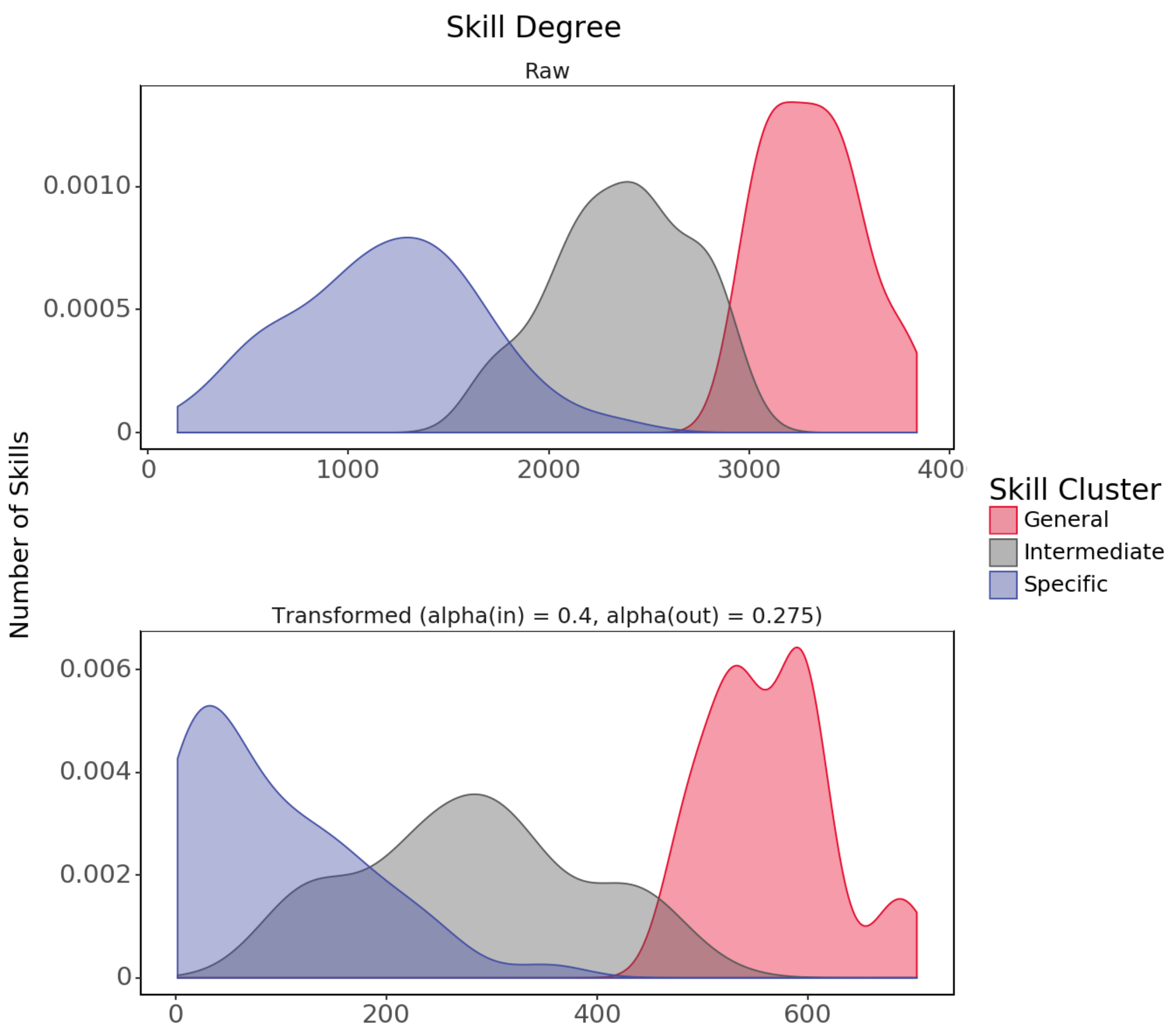

Supplementary Figure 11: Skill Degrees as Validity Check on Choosing Parameters of Obtaining Skill-occupation Network Backbone. The figure compares the degree distribution of skills in each skill group before and after the transformation. Our emphasis is on the distinction between the distribution of three types of skills (their overlap) and their relative position to the raw data. Indeed, the Pearson correlation between skills' strengths (sum of edge weights) and their transformed degree is 0.95. The ranking of skills across these two measures is also preserved (correlation is 0.97).

Where $\sigma^2 = \frac{N(u).N(v)}{\|O\|}\frac{\|O\|-N(v)}{\|O\|}\frac{\|O\|-N(u)}{\|O\|-1}$ and $\mu = \frac{N(u).N(v)}{\|O\|}$, are the standard deviation and mean of a hypergeometric distribution for the expected co-occurrence of skills (that arise the under the null model of a bipartite configuration model that preserves skill degrees [15].) $N(u)$ and $N(v)$ denote the number of occupations that demand skill u and v, respectively and $\|O\|$ denotes the total number of occupations.

We now estimate conditional probabilities $P(u|v)$ and $P(v|u)$ and assign a direction to them. The direction, $u \rightarrow v$, is determined when $P(u|v)$ is *substantially* greater than $P(v|u)$. Once again, we wouldn't consider every $P(u|v)$ that is insignificantly greater (smaller) than $P(v|u)$, but only those that are sufficiently greater (smaller) to be considered as a *dependent* structure. α_{th} sets the minimum difference between two conditional probabilities so that they are considered to have directional dependence. This threshold has to be differentially applied to each skill pair due to the heterogeneous skill node degrees. Therefore, the threshold α_{th} is weighted by $(\frac{k_{max}}{min(k_u,k_v)})$ to be applied to filter $[P(u|v) - P(v|u)] \neq 0$

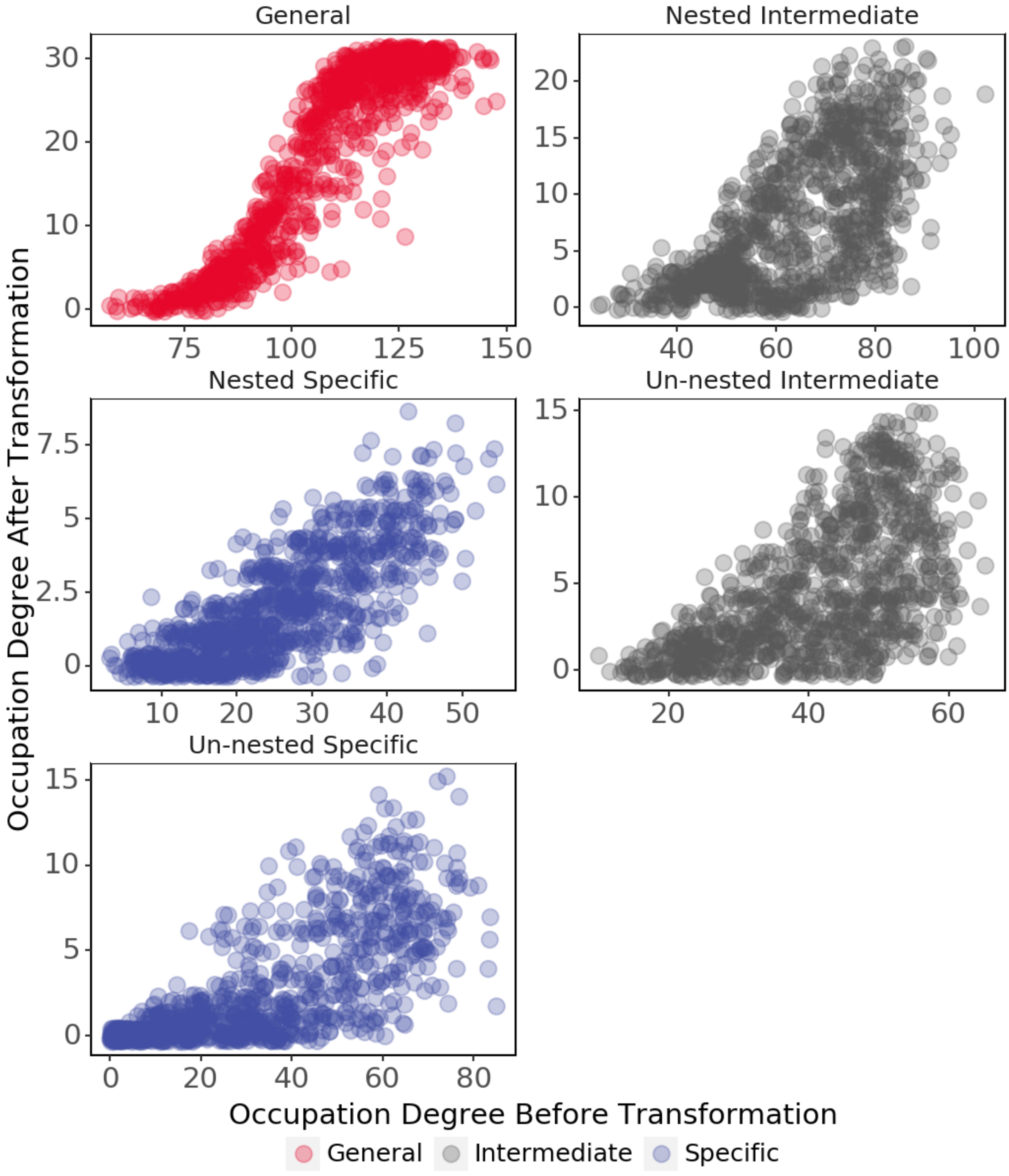

Supplementary Figure 12: Occupational Degrees as Validity Check on Choosing Parameters of Obtaining Skill-occupation Network Backbone. The figures compare the degree distribution of occupations before and after the transformation. Our emphasis is faithfulness across each of the five skill subtypes.

$$|P(u|v) - P(v|u)| > (\frac{k_{max}}{min(k_u, k_v)}) \times \alpha_{th} \quad (6)$$

Where k denotes the number of other skills with ties to the focal skill, and k_{max} denotes the biggest degree observed among skills.

The magnitude of the dependence between u and v, $w_{u \to v}$, follows the parametric function

introduced by [15]:

$$w_{u \to v} = \frac{min(k_u, k_v)}{k_{max}} \left(\frac{N(u,v)}{N(v)} - \frac{N(u,v)}{N(u)} \right) \tag{7}$$

In simple terms, the direction of arrows shows whether by observing skill v in an occupation, it is (more) likely also to observe skill u (than the other way around). The magnitude of dependence, used as weights in the main Fig. 2, is a parameteric function of the difference between the conditional probabilities of observing u and v, and the null model that corresponds to the estimated number of shared occupations between them, given the degrees of u and v. The final network is shown in Fig. 15 and used across all analysis, but for the main Fig. 2 and 7 that depict the parsimonious versions, from a directed acyclic graph (DAG) [15].

Choice of Parameters

There are two parameter choices z_{th} and α_{th}. Here, we present results across different parameters to ensure the robustness of our findings. We choose the first parameter, z_{th}, in such a way that we remove about two-thirds of the edges. Fig. 13 shows the distribution of z-scores for all skill co-appearance edges.

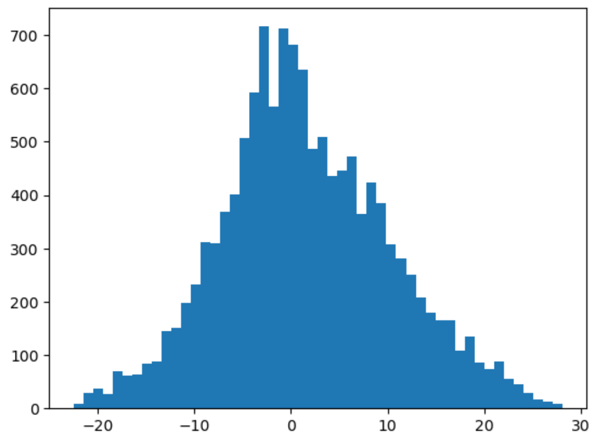

Supplementary Figure 13: Histogram of Z-scores Resulting from Equation 5 for Skill Co-appearances. The x-axis shows the z_{th} values derived from equation 5 on our data, and the y-axis shows the number of co-appearance links falling into a given range of z_{th} values. We focus on z_{th} values between 4 and 6.

Ideally, one chooses α_{th} as strictly as possible to remove insignificant links without the loss of skills. If all edges associated with a given skill are removed as a result of the two steps of the algorithm, the skill is eliminated from the resulting dependency network. However, as our sensitivity analysis shows, retaining too many statistically insignificant links weakens our ability to extract conditional dependencies robustly. Therefore, retaining more statistically significant edges inevitably impose the cost of losing several skills. Fig. 14 offers a sensitivity analysis on the interaction of z_{th} and α_{th}. Ideally, no more than 5% of skills are eliminated, while about 95% of ties between skills were removed as statistically insignificant. The combination $z_{th} = 4.75$ and $\alpha_{th} = 0.05$ is a possible solution used in the main text. At this level, only five skills are eliminated from the network.

A consideration is whether the shape of the skill dependency in Fig. 2 (b and c) is influenced by the choice of parameters. We conduct a robustness check wherein we visualize the resulting networks from the combination of values of z_{th} between 4 and 5.5 and values of α_{th} between 0.01 and 0.1.

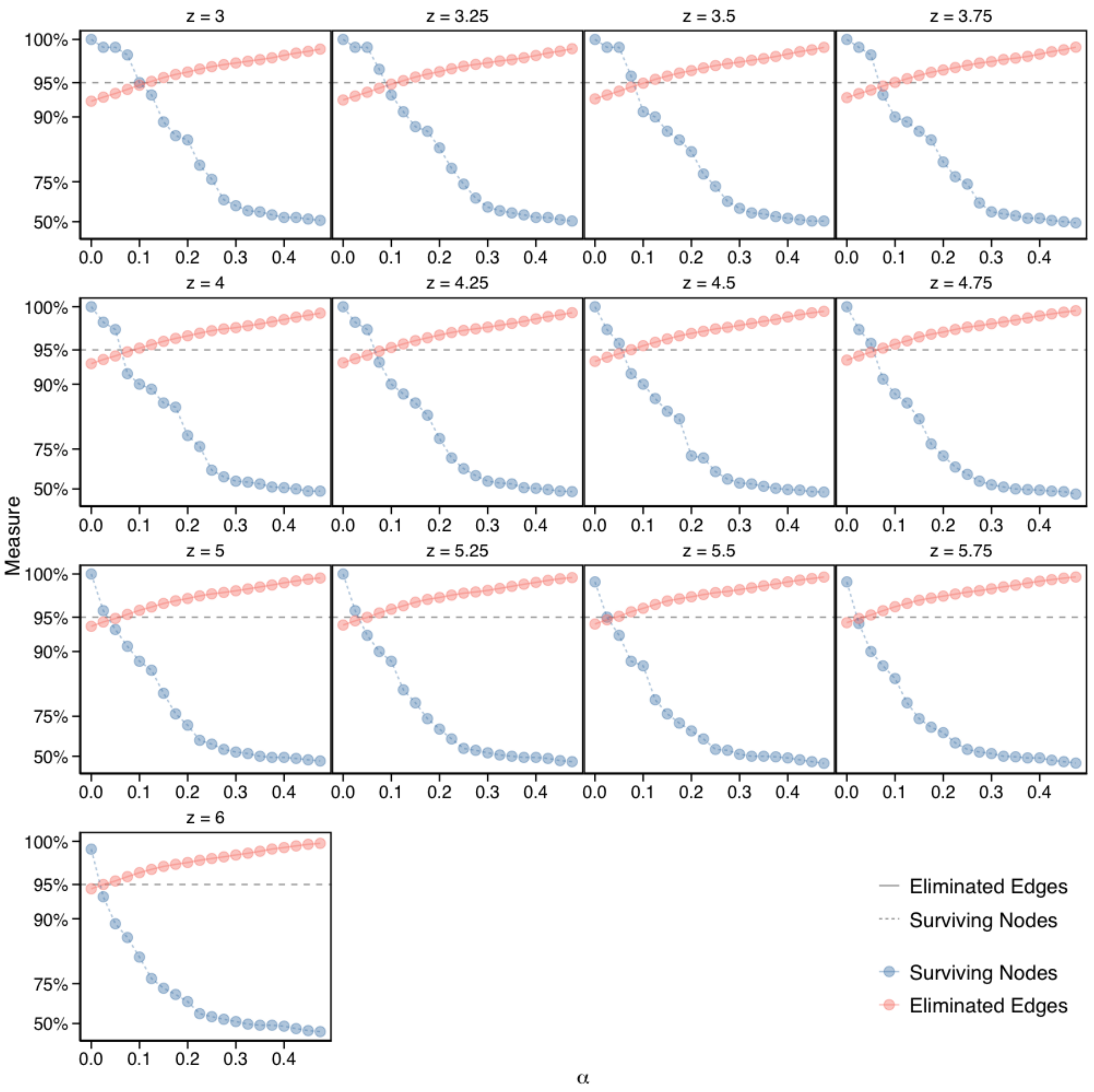

Supplementary Figure 14: Sensitivity Analysis on Parameters Used for Obtaining Significant Skill Dependencies. The x-axis shows the $\alpha_{threshold}$ values. Each panel shows a certain $z_{threshold}$ as in equation 5, and the y-axis shows the rate of node survival or edge elimination. Ideally, no more than 5% of skills are eliminated, while about 95% of ties between skills were removed as statistically insignificant. $z_{threshold} = 4.75$ and $\alpha_{threshold} = 0.05$ is a possible solution used in the main text. The resulting backbone from a number of combinations is offered in the following.

Throughout, a disjointed structure emerges, wherein a set of specialized skills (blue and gray) have closer connections with the general skills (red), than other specialized skills. Even in networks obtained from a lenient α_{th}, the hierarchical structure is visible, and in most, one can distinguish between a more closely knit web of skills that manifest stronger dependence on generals skills (manifest higher connection to red nodes), and a second set of skills, decoupled from the first, which manifest a comparatively shallow dependency web. We withdrew the visualized network for the sake of brevity. These visuals are sharable upon request.

Visualizing the Skill Hierarchy

The main Fig. 2 is the backbone of a network with parameters $z_{th} = 4.75$ and $\alpha_{th} = 0.05$. Fig. 15 shows the full skill network, which contains 115 nodes and 1,796 dependency relationships. The following skills are eliminated from the graph because neither of their dependency relationships passed the statistical significance test: *Installation, Explosive Strength, Sound Localization, Food Production, Public Safety, and Security*. In the backbone network, any direct path is eliminated where there exists an indirect path through dependencies. As a result, the backbone contains only 395 edges, accommodating visualization. Nonetheless, we perform all calculations on the skill network and not its backbone.

For both the skill network and its backbone, we use a layout technique inspired by [16, 17]. We determine the vertical placement of a skill based on its local reaching centrality [16], defined as the number of nodes achievable from the focal node. This highly correlates with a skills' demand, defined as the total level values, across occupations (Pearson correlation 0.89). The horizontal position is proportional to the skill's association with education, calculated as the weighted education of occupations (using levels as weights). We pass normalized values for both the vertical and horizontal axes through a Lambert cylindrical projection to Gephi for visualization. Fig. 16 shows the network in the main Fig. 2 (b) with all nodes labeled and the position of nodes adjusted to accommodate labels.

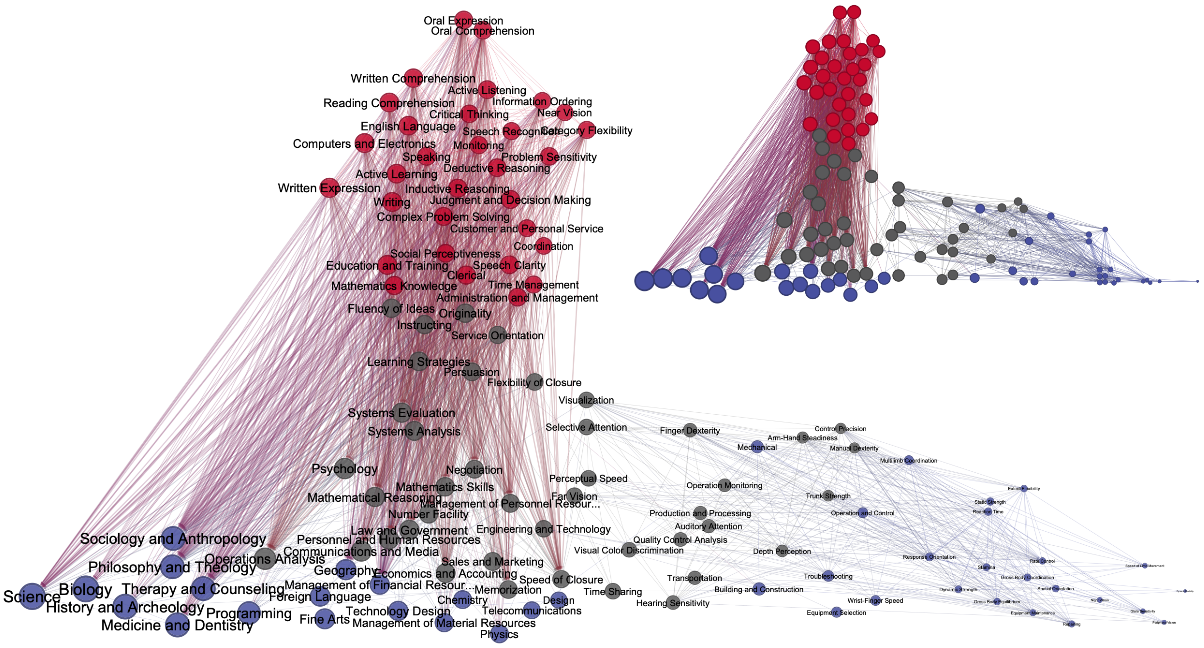

Supplementary Figure 15: Skill Dependency Network. The layout is adjusted to accommodate the labels. The original layout (without overlap) is shown in the top right corner.

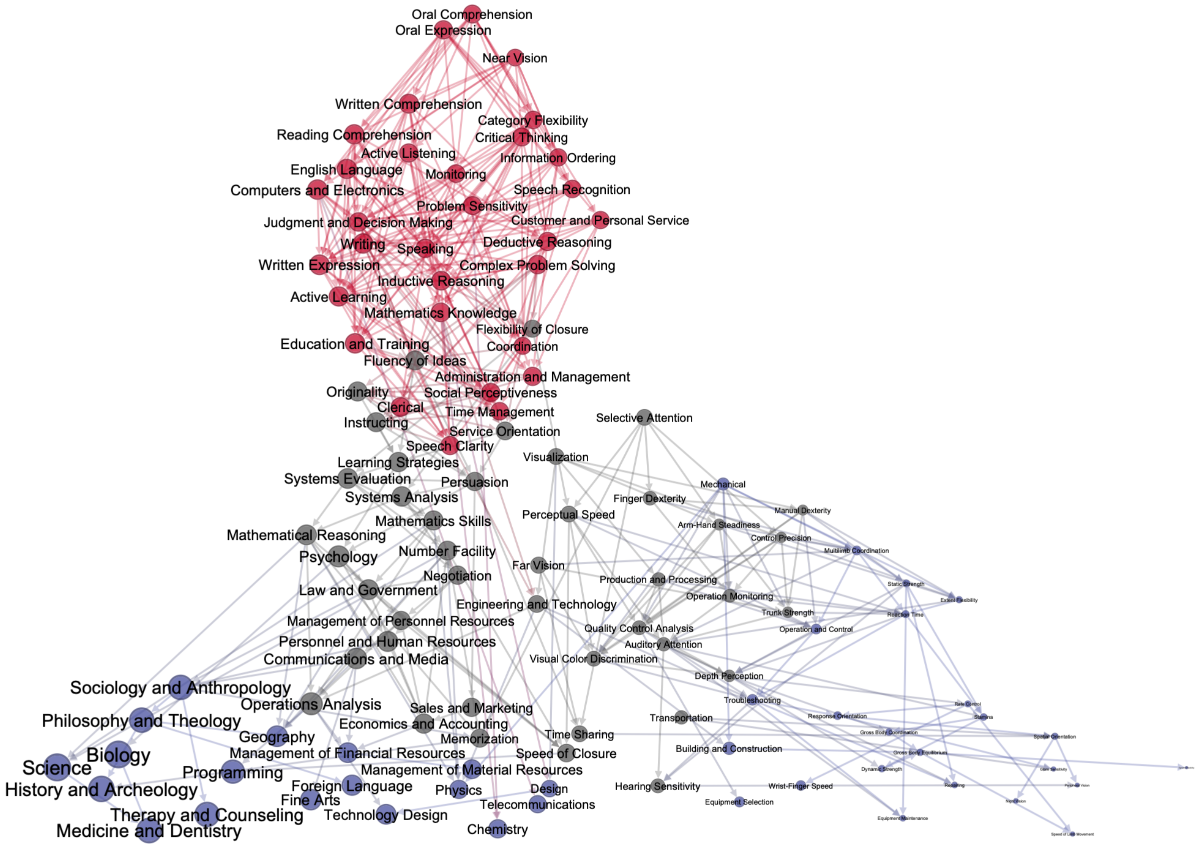

Supplementary Figure 16: Labeled Skill Dependency Backbone. Node positions are adjusted to accommodate labels.

3.3 Linkage to Skill Co-occurrence Networks

Our work builds on a vast literature that conceptualizes the landscape of skills as a co-occurrence network [18–21]. Indeed, without directionally, the hierarchical network is in excellent agreement with such previously constructed skill networks. Here, we follow the approach used in [20] that identifies communities of cognitive versus physical from a pairwise co-occurrence network. We obtain such a network in two steps (using O*NET skill data from 2019):

1. Measuring the "effective use of skill" by occupation based on RCA as follows:

$$RCA(s,j) = \frac{Importance(s,j)/\Sigma_{s' \in S} Importance(s,j')}{\Sigma_{j' \in J} Importance(s',j')/\Sigma_{s' \in S, j' \in J} Importance(s',j')} \tag{8}$$

   where s denotes a given skill, and j a given occupation. S and J denote the population of skills and occupations respectively. An skill-occupation is 'effective'— i.e., $e(i,j) = 1$ if $RCA(s,j)$— and is not— i.e., $e(i,j) = 0$, otherwise.

2. Using $e(i,j)$ values, authors derive pairwise skill "complementarity" proportional to the number of times skills s and s' co-appeared in an occupation as follows:

$$\theta(s,s') = \frac{\Sigma_{j \in J} e(s,j).e(s',j)}{max\big(\Sigma_{j \in J} e(s,j), \Sigma_{j \in J} e(s',j)\big)} \tag{9}$$

There are two key messages. First, we explain that the cluster of *General* skills resides at the center of such a skill co-occurrence network— in fact, the ordering of skill specificity based on our skill clusters is predictive of how far the skills lie towards the fringes of the skill co-occurrence network. Second, the dichotomy of cognitive versus non-cognitive skills has tight connections with the disjointed structures we found and called nested and un-nested skills, respectively.

Fig. 17 shows a network representation of skills based on the pairwise ”complementarity” values manifests the bi-modal structure reported by [20]. There are several departure points, however. First, we restrict our workplace skills to the so-called *knowledge, abilities, and skills*, disregarding *work activities*, while the latter is commonly used in co-occurrence networks constructed using O*NET. Our rationale for not including work activities is that they are job descriptions (i.e., generalized forms of job tasks that are specific to jobs). In contrast, abilities, knowledge, and skills are characteristics of workers' expertise, which are our primary concern.

Second, the community on the right is an ensemble of ”cognitive” skills, while the left group corresponds to mostly ”physical” skills, which are consistent with our nested and un-nested skills, respectively. However, several skills, such as *Physics, Design, and Chemistry* seem out of place at the bottom end of the left community, and are classified by our approach as nested skills— which appear consistent with their wage and educational associations. The key advantage of our method is that we can predict numerous implications of skills based solely on the informationed embedded in the occupation-skill networks, without the need for knowing the content of the skill, as labeled by cognitive or physical skills. Our skill hierarchy, in effect, offers an explanation for why certain skills, known to be cognitive, are more valuable, based on the investments necessary to satify their dense and nested web of dependencies.

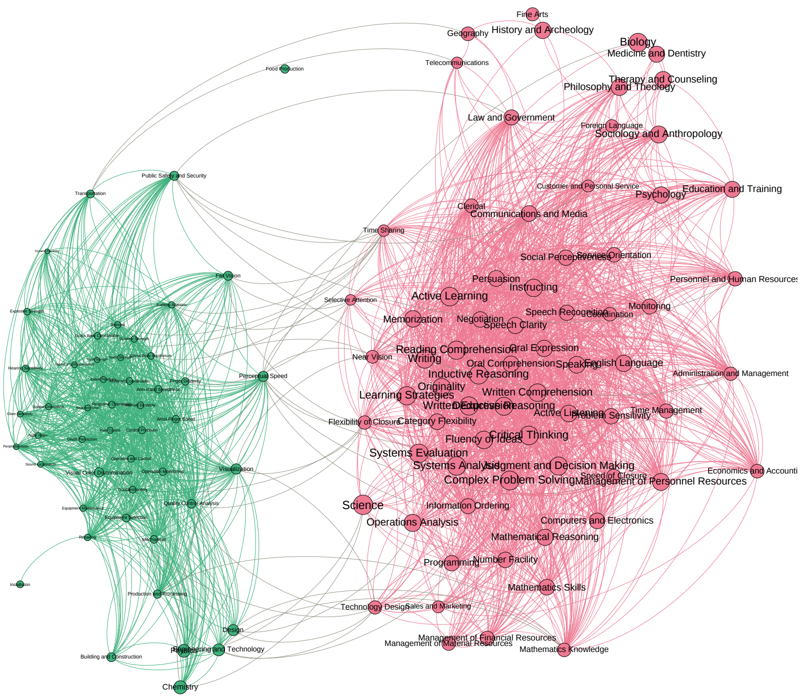

Supplementary Figure 17: Network of Pairwise Skill ”Complementarity.” In this figure, edges denote skill complementarity relationship if they passed the threshold of $\theta(s,s') > 0.5$— authors used a threshold of $\theta(s,s') > 0.6$ which leads to several isolated nodes in 2019 data. We assign an average education value to each skill based on the educational requirements of occupations. A skill's associated education level is only impacted by occupations that use the skill 'effectively'— i.e., $\forall j : RCA(s,j)$, nodes are colored based on their modularity communities.

3.4 Skill Hierarchy Captures Career Progress (Specialization)

A key advantage of integrating the conceptual distinction between general and niche skills with a structural network approach to studying skills is that the aggregation of pairwise skill interdependencies reveals pathways of progress (what has come to be known as "specialization"). However, the structure of our skill hierarchy implies that progress entails co-development in certain niche skills and the prerequisites, often more general skills.

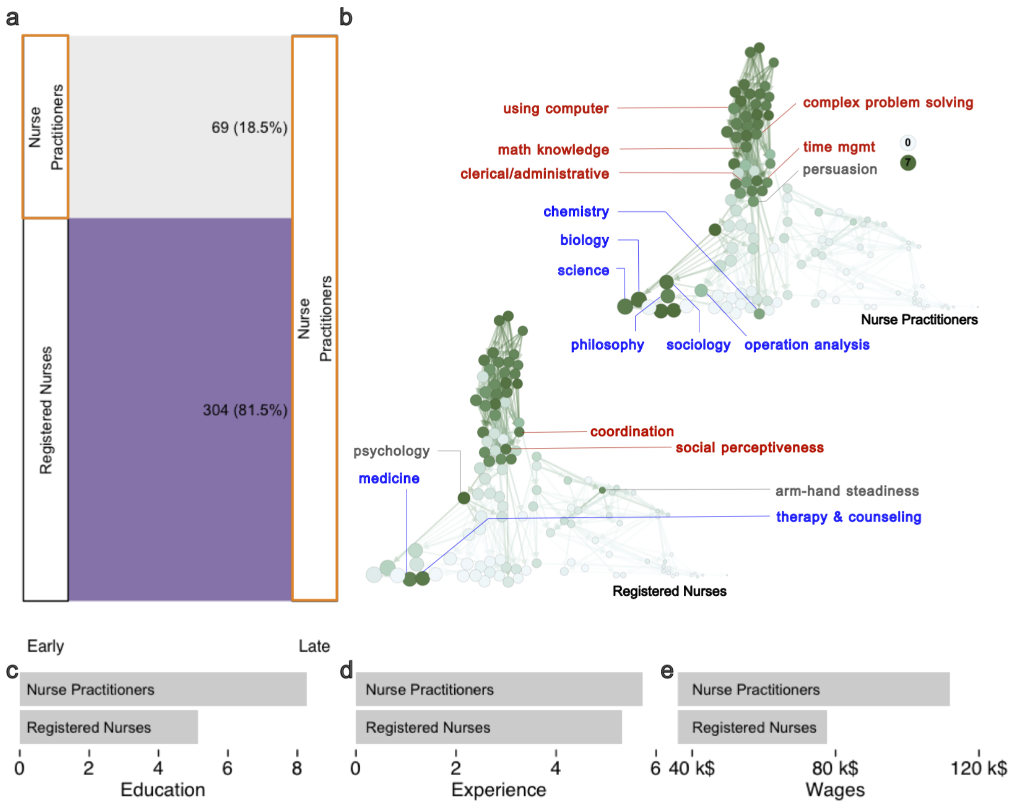

Supplementary Figure 18: Transition between Registered Nurses (RNs) and Nurse Practitioners (NPs). (a) uses resume data from Burning Glass Technology to capture the transition statistics between RNs and NPs. We restrict the analysis to individuals with at least five listed occupations in their resume and define their early career occupations as the most appeared occupation in the first three jobs, similarly late career occupations as the most appeared in the fourth jobs and onward. We disregard individuals whose early and late careers are neither RN nor NP. Including these individuals would not change the result but significantly complicate the exposition. One expects that higher wages for NPs would attract RNs **(e)**. Most NPs were RNs early on. However, only a subset of RNs progresses to NP jobs, suggesting barriers to entry, summarized in higher experience and educational requirements **(c-d)**. **(b)** captures the skill requirements of RNs and NPs, highlighting the advantage of integrating the conceptual distinction between general and niche skills with a structural network approach to studying skills in revealing pathways of progress (also known as "specialization"). Our skill hierarchy structure also implies that progress entails co-development in certain niche skills and the prerequisite, often more general skills.

Here, we explore a case study of such progress based on the skill requirement differentials of registered nurses (RNs) versus nurse practitioners (NPs). Compared to RNs, NPs prescribe medicine and diagnostic tests and command higher wages (Fig. 18 e). Without any cost, someone equipped with the skills of an RN would ideally prefer to work as a nurse practitioner to benefit from higher payoffs. However, as Fig. 18 (a) shows, only a subset of individuals who are RNs early on in their careers (i.e., for whom RN appears most in their first three jobs listed in their Burning Glass Technology resume) manage the switch to the better-paid NP jobs later in their careers (i.e., NP appears most after their third jobs listed in their Burning Glass

Technology resume). The fact that most NPs were initially RNs (81.5%) corroborates our interpretation of the path from RN to NP as one that entails career progress. The transition statistics captured in Fig. 18 (a) are also consistent with the higher experience and the more extended training needed for nurse practitioners to develop the necessary skills (Fig. 18 c-d).

The correlation between education and wages observed at the cross-section of RNs and NPs agrees with the economic theory narrative. However, only a structural approach can reveal the skill development involved in such a transition, highlighting skill growth pathways, seen in Fig. 18 (b). While RNs require high levels of medicine and therapy (niche skills), psychology (intermediate skill), coordination, and social perceptiveness (among many other general skills), the transition into an NP requires further levels of those skills as well as significant development of science, biology, chemistry (among other niche skills), persuasion (intermediate skill), as well as higher knowledge of math, time management, complex problems solving, administrative and computer skills (among other general skills). In contrast, arm-hand steadiness used at high levels by RNs is not as intensely utilized by NPs.

Comparing RNs' with NPs' skills showcases that our approach teases out meaningful progression (or specialization) pathways embedded in the skill requirement of occupations. The co-development of niche and the relevant general skills underpin what we call a nested specialization path. In the following, we offer evidence that the pattern observed in RNs' and NPs' careers emerges across individuals in other occupations.

3.5 Skill Hierarchy Captures Skill Entrapment

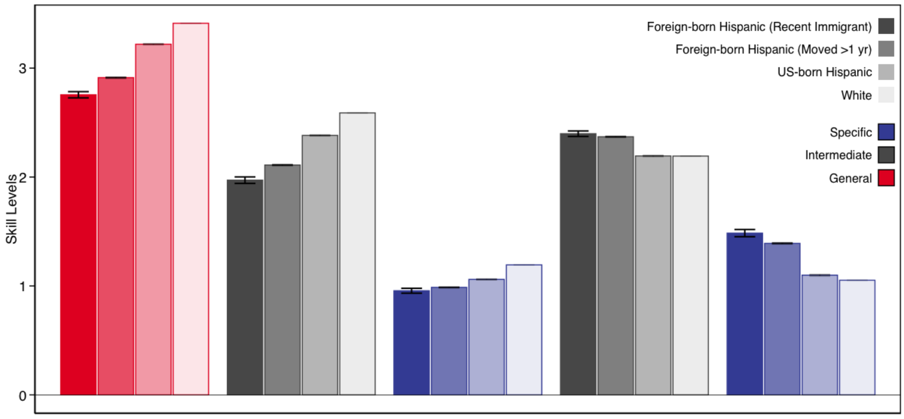

Supplementary Figure 19: Comparison of the Skill Levels of Hispanic Immigrants and White Workers. We distinguish between four groups of workers (i. foreign-born Hispanics who have migrated less than a year to the US from the time of survey ($n = 1,618$), ii. foreign-born Hispanics who have been in the US for more than a year ($n = 106,866$), iii. US-born Hispanics ($n = 76,509$), and iv. the White workers($n = 680,743$)) and map their average skill levels (with 95% CI) for each skill category. Recently migrated foreign-born Hispanics have the least levels of general and nested skills and most un-nested skills.

As the main Fig. 6 and SI Sec. 7 show, Hispanics tend to possess relatively high levels of un-nested skills but are underprivileged in gender nested skills. This unbalance leads to skill entrapments with possibly early rewards and long-term wage penalties, as SI Fig. 31. Our skill hierarchy allows us to explore one possible driver of this skill unbalance for Hispanics.

We suspect language skills are barriers to some hispanic workers, particularly early on in their careers in the US, hampering the acquisition of (language-related) general and (the downstream) nested specific skills, but less so the acquisition of un-nested skills. To test this, we split the sample of individuals from the CPS into four subgroups, ordered based on their

likely level of English proficiency: *Hispanics born outside of the US who immigrated less than a year before the survey, Hispanics born outside the US who have been in the US for more than a year, Hispanics born inside of the US, and White workers*. We map the average skill levels of each of the above subgroups for each skill category in Fig. 19, below. As hypothesized,the foreign-born Hispanics who recently migrated to the US have the lowest levels of general and nested skills and have the highest unnested skills. The suspected ranking of English proficiency of each subgroup is consistent with their ranking in terms of general, nested and unnested skills. Next, we investigate the role of language skills directly Fig. 20.

Our network allows us to directly identify which nested skills more closely depend on language general skills. To do so, we first identify six general skills as "language-related": i. English Language, ii. Oral Expression, iii. Oral Comprehension, iv. Written Expression, v. Written Comprehension, and vi. Speaking. One can quantify the dependence of each nested skill, i, on each of the mentioned language skills, j, by deriving the arrival probability of a random walk starting from the mentioned language general skills, $P_{i,j}^{<arrival>}$. Aggregating these probabilities over the language general skills, we obtain $P_i^{<arrival>} = \Sigma_j P_{i,j}^{<arrival>}$. We flag nested specific skills at the top 25% of skills in terms of their average arrival probability, $P_{i,j}^{<arrival>}$, obtaining the following skills: i. History & Archeology, ii. Management of Material Resources, iii. Management of Financial Resources, iv. Programming, v. Philosophy & Theology. Splitting general and nested skills by their language associations (general skills into Language-related and Non-language skills, and nested skills into Language dependent and Language independent), we obtain the average skill levels of individuals for the previously defined subgroups of workers (Hispanic and White based on their place of birth and time since immigration). In Fig. 20, we show the ratios of skills levels for the different Hispanic subpopulation groups relative to White workers for the Language-related and non-language general skills and Language-dependent.

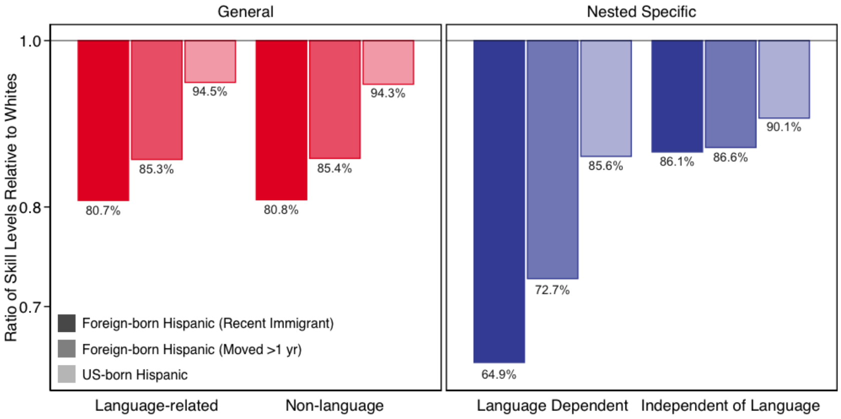

Supplementary Figure 20: Language Barriers Manifest in Lower Levels of Language-related Nested Skills for Hispanics. The figure depicts, for the Language-related and non-language general skills and Language-dependent (defined as the skills in the top 25% arrival probability to the mentioned language skills) and Language-independent nested skills, the ratios of skills levels for the different Hispanic subgroups relative to White workers. The results depict that the language-dependent nested skills vary significantly more across the Language-dependent subset, supporting our suspicion that Hispanic workers, at least in part, suffer from their language skills, which prevents them from acquiring/applying downstream skills.

The results show that the skill gaps between Hispanic subpopulations and White workers mimic the implied language gradient: the less proficient in English a subgroup will be, the larger the gap is to White workers in language-dependent nested skills but not in language-independent specific skills. This supports our hypothesis that the skill gaps for Hispanic workers as a whole are, at least in part, due to language barriers.

4 Skill Categories in Career Trajectories

Main Fig. 4 supplements our inference of the skill structure from O*NET, which relies on cross-sectional data, with longitudinal evidence in line with the notion that one actually acquires or advances general skills when they progress in their career and acquire more specific skills. Here, we provide additional evidence and robustness checks on the analysis of main Fig. 4, based on resume (Burning Glass) data, occupational median age, and skill acquisition reflected in synthetic birth cohorts we created using CPS microdata.

4.1 Resume Data

Unlike O*NET, Burning Glass resume data offers longitudinal observation of skill acquisition and will allow us to conduct a more strict test of our skill structure. We keep track of one's occupations in the resume data, from which longitudinal skill acquisition is inferred.

Preparing Burning Glass Data

The following discussion describes choices made in cleaning the data, revealing robustness to such choices in terms of the direction of the results, although the magnitude may vary slightly. We studied over 20 million resumes from the Burning Glass data, which amounts to over 70 million job moves. For each move, we link the source and destination occupations to skills from O*NET in 2019. Excluding all within-occupation moves— which amount to no skill change— we calculate a skill level change across our skill categories and show the result. Fig. 21 as the distribution of career moves for resumes in the Burning Glass sample— after removing within-occupational career moves.

As can be seen in Fig. 21, A minority of career moves produce extreme values, stretching the skill change distributions' tails. Table 4 shows a few such cases from the data. For instance, the resume with the ID *652855*, serves as a janitor for a short period (4 months) before seemingly claiming a Chief Executive role. Resume with ID *1723696* held overlapping jobs as a Medical Health Technician and a Middle School Teacher. Studying the career moves that correspond to such skill changes, we noticed a significant proportion arise from short job stints and coinciding jobs— some seemingly voluntary part-time commitments.

We removed such jobs from our resume sample. Particularly, we kept jobs if they lasted at least 12 months— we arrived at the threshold after studying the career moves that correspond to the thousand largest absolute skill changes. Furthermore, sorting jobs for each resume based on starting date and end date, we removed any job that had a shorter length and overlapped with another— that is, we remove a job j_r from a resume r, if it had a later or equal start date with another job j'_r, but did not have a later end date. We also removed jobs for which we could not extract the start and end date— we used *Python's dateparser version 1.1.1.* for the extraction. The resulting sample was 9,382,602 career moves and 5,361,751 resumes. Fig. 22 shows the resulting skill change distributions.

The main text analyzes the levels and patterns of skill change across general and (nested and un-nested) specific skills. Fig. 23 shows the net effects, i.e., the average change in levels resulting from job moves across all skill categories, and the correlation between the change in the levels of general skills and changes in the level of other skill categories resulting from job transitions. Fig. 24 supplements main Fig. 4 (i) by providing the changes in all skill category levels resulting from consecutive job transitions.

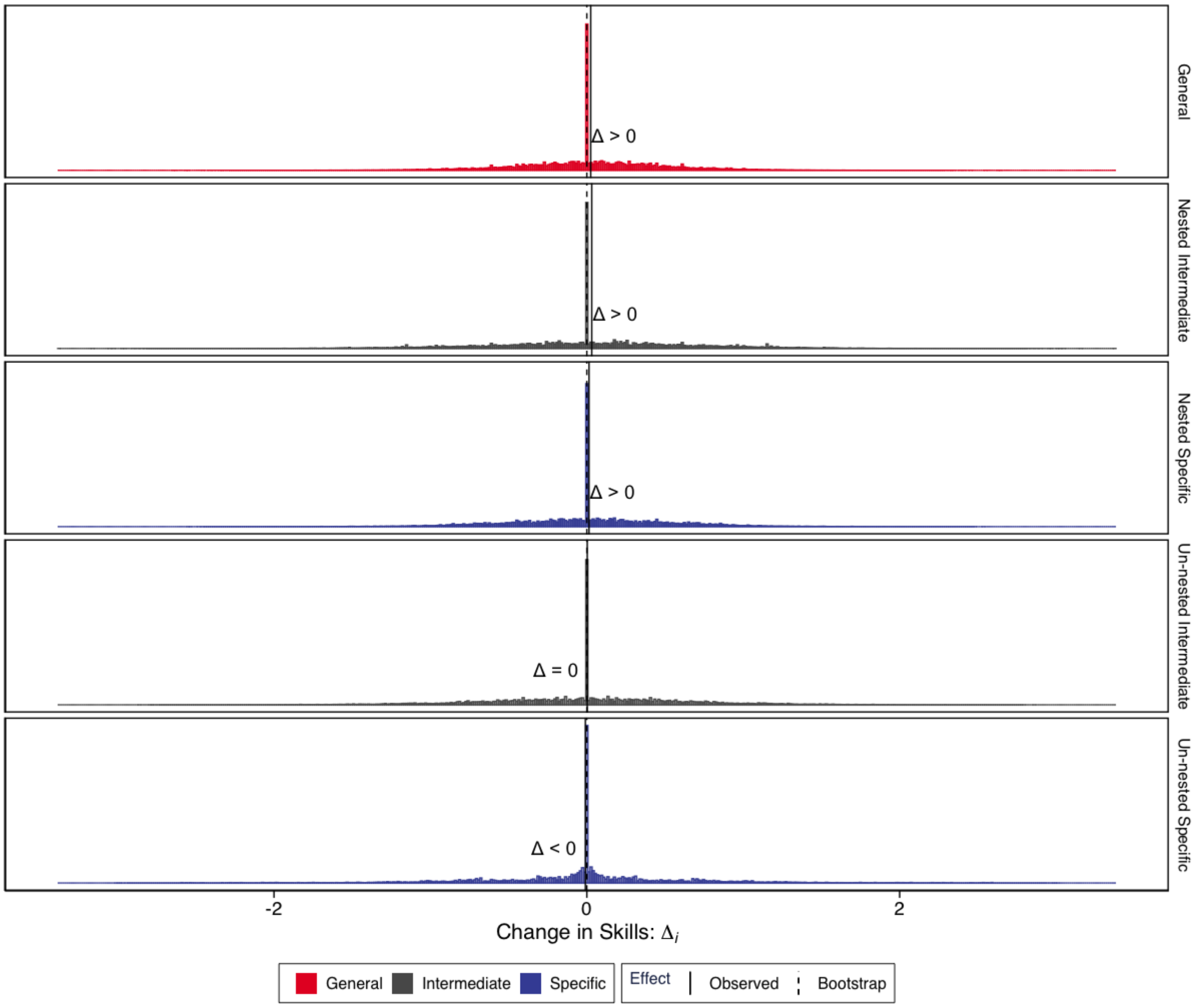

Supplementary Figure 21: Changes in Skill Levels in Individuals' Career Moves. The distribution shows the Burning Glass resume data. A minority of career moves produce extreme values, stretching the skill change distributions' tails due to imperfect data.

Supplementary Table 4: A Select List of Job Sequences in Burning Glass data that yield extreme Skill Changes.

	ID	Start	End	Occupation
1	652855	Oct 2011	Feb 2012	Janitors & Cleaners, Except Maids & Housekeeping Cleaners
2		Jun 2012	Aug 2012	Chief Executives
3	1723696	Sep 1981	Sep 1991	Janitors & Cleaners, Except Maids & Housekeeping Cleaners
4		Sep 1991	Sep 1994	Medical Records & Health Information Technicians
5		Sep 1993	Sep 1993	Middle School Teachers, Except Special & Career or Technical Education
6		Sep 1994	Sep 1999	Lodging Managers
7	18075175	Jun 2007	Jun 2007	Chief Executives
8		Aug 2009	Aug 2009	Janitors & Cleaners, Except Maids & Housekeeping Cleaners
9		Aug 2010	Aug 2010	Cashiers
10		Aug 2011	Aug 2011	Retail Salespersons
11		Dec 2012	Dec 2012	Retail Salespersons
12		Feb 2013	Feb 2013	Cashiers
13	18325881	Jun 2022	Oct 2022	Medical & Health Services Managers
14		Oct 2022	Jan 2022	Medical & Health Services Managers
15		Jan 2022	May 2022	Human Resources Specialists
16		May 2022	Sep 2022	Models

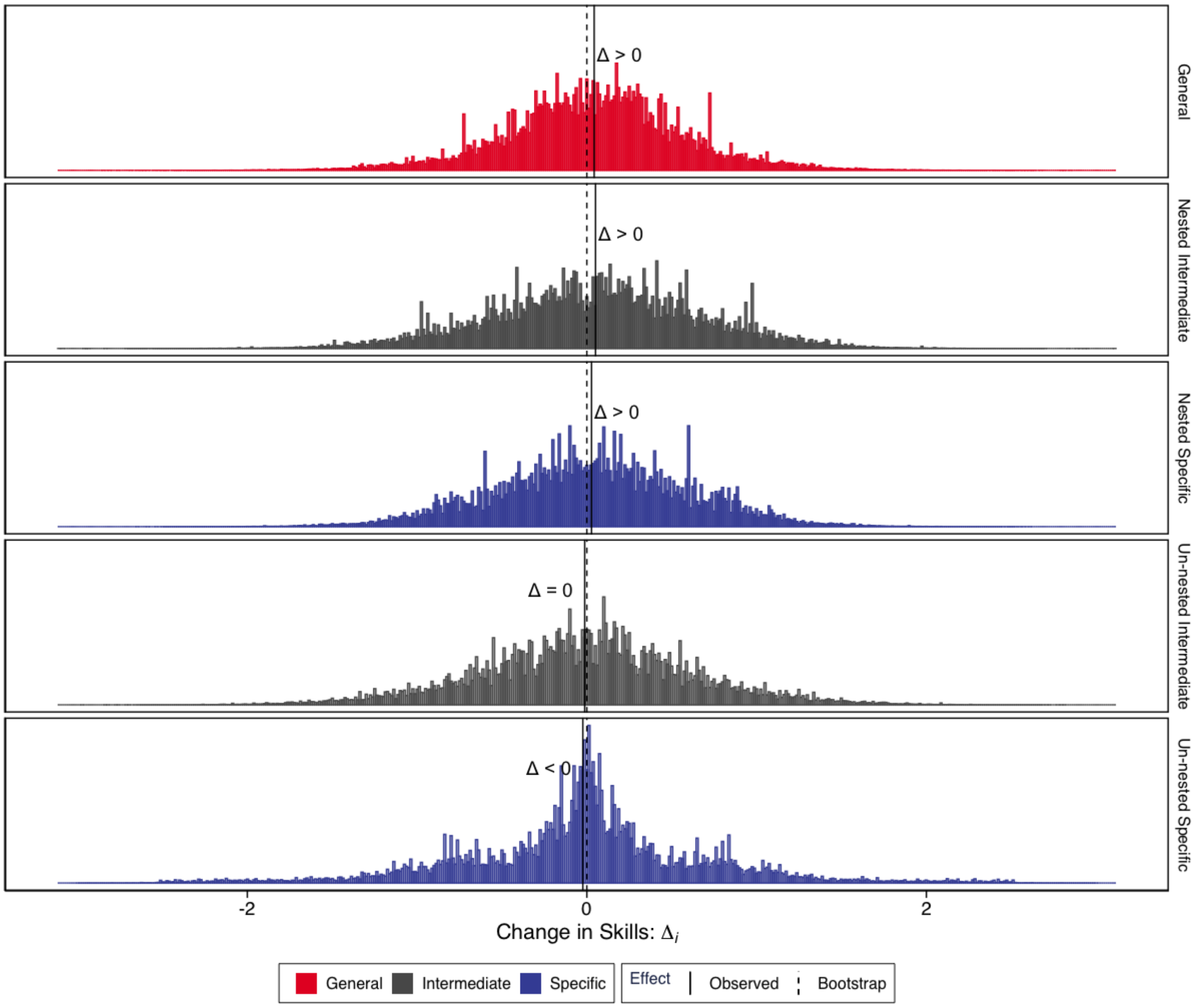

Supplementary Figure 22: Changes in Skill Levels in Individuals' Career Moves. Most career moves amount to small changes in skills. On aggregate, general, nested skills experience increases on aggregate, while un-nested skills record non-positive changes. Nested skill changes closely correlate with changes in general skills. In contrast, there is almost no noticeable relationship between changes in general and un-nested skills. Importantly, randomizing the sequence of job transitions (bootstrap) eradicats the direction of skill acquisition in the observed data.

Expected Skill Change from Random Job Transition

Furthermore, we bootstrapped our resume sample to produce a benchmark and compare it with the skill changes we obtained from observed career moves. For each resume in our sample, we randomly permuted the order of career moves and measured the skill changes again. Fig. 25 shows one such bootstrap. It is visible in Fig. 25 that the randomization eradicated the direction of skill changes we had obtained from the observed career moves— in Fig. 22.

Fig. 26 further shows the distribution of average skill changes for 100 bootstraps. The fact that resulting skill changes from a null model differ significantly from our observed results ensures our results are meaningful signals of individuals' career moves, pointing to the dependencies between (general and nested) skills.

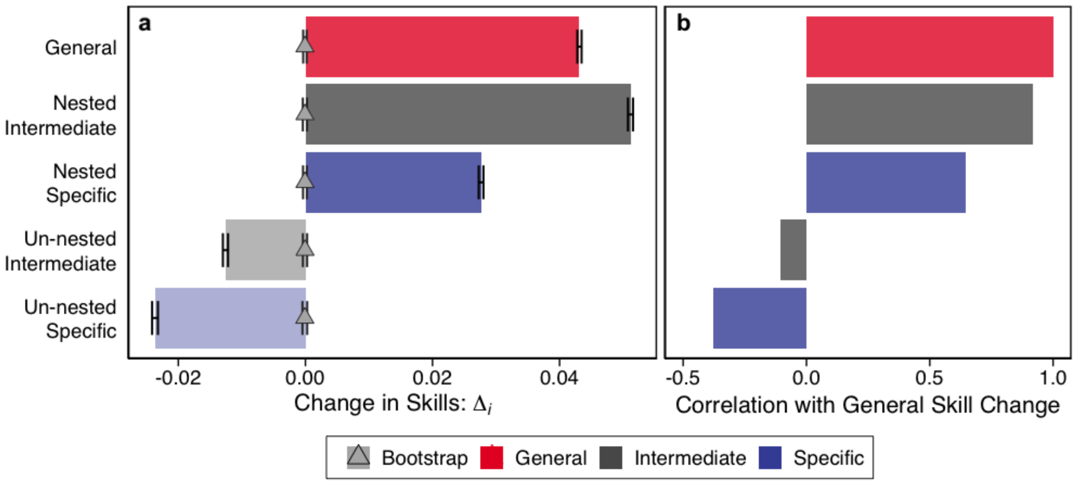

Supplementary Figure 23: Expected Changes of Skill Levels for Each Career Moves. For each career move ($n = 1,493,142$), we linked the source and destination occupations to skills from O*NET in 2019. We calculate a skill level change across our five skill subtypes. For each skill sub-type, we measure changes in skill levels, Δ_s, corresponding to each career move as the average of differences between the skill levels of the target and source occupations. **(a)** shows average changes (with 95% CI) in skill levels for skill subtypes. On aggregate, general, nested skills experience increases on aggregate, while un-nested skills record non-positive changes. **(b)** shows the correlation between general skills and each skill category resulting from individuals' career moves. Nested skill changes are closely related to changes in general skills. In contrast, there is almost no noticeable relationship between changes in general and un-nested skills.

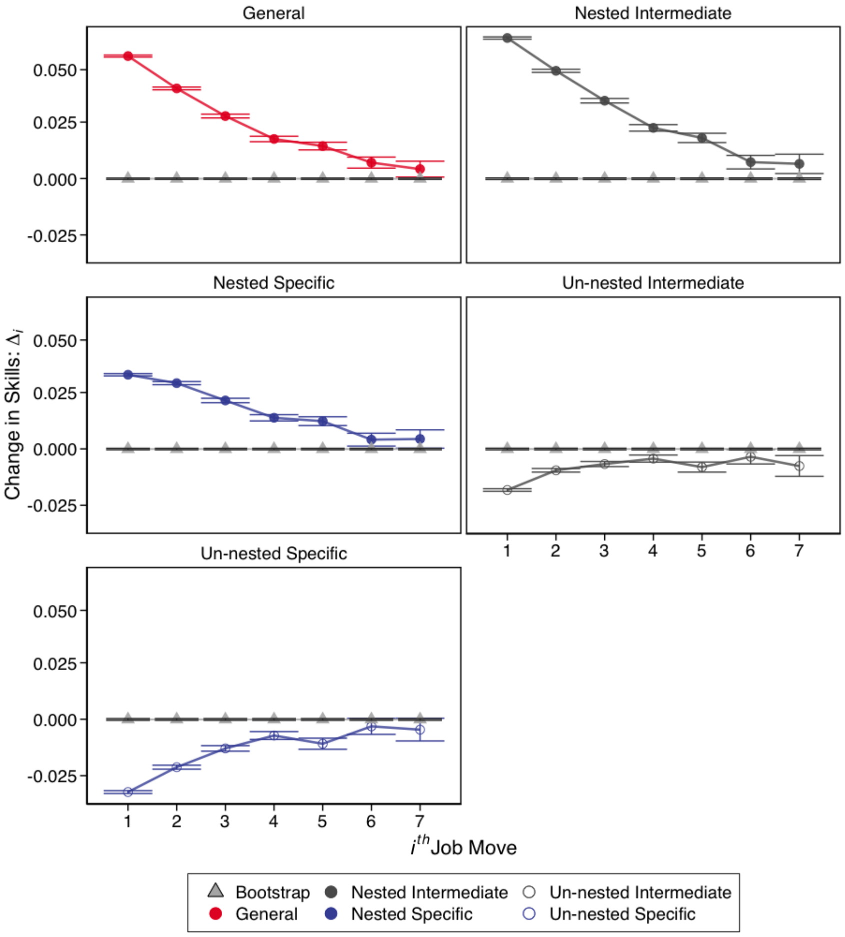

Supplementary Figure 24: Average Changes in all skill category levels in consecutive job transitions. Error bars show 95% CI ($n = 1,493,142$).

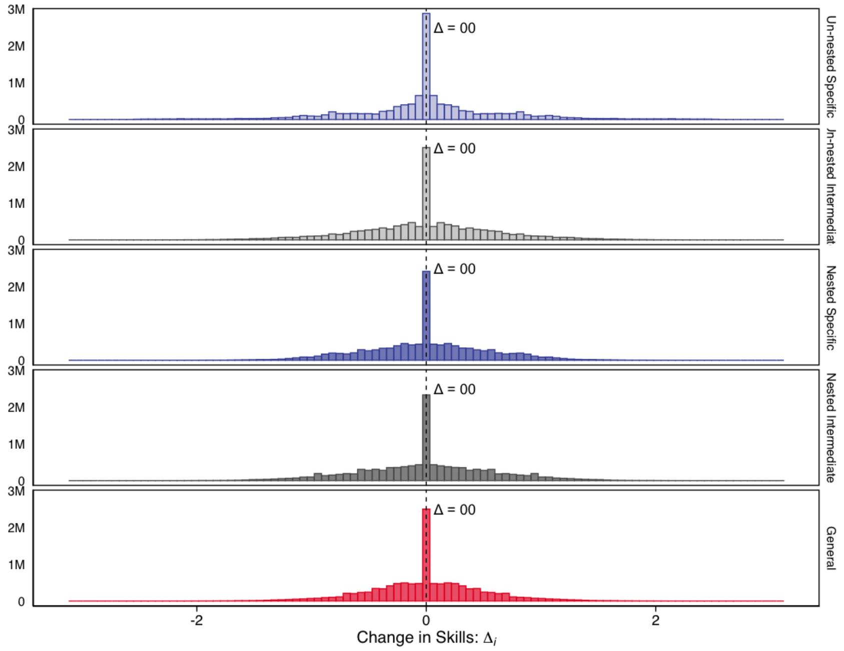

Supplementary Figure 25: Changes in Skill Levels in *Bootstrapped* Individuals' Career Moves. The distribution of changes in skill levels visibly differs from what we obtain from the observed career moves.

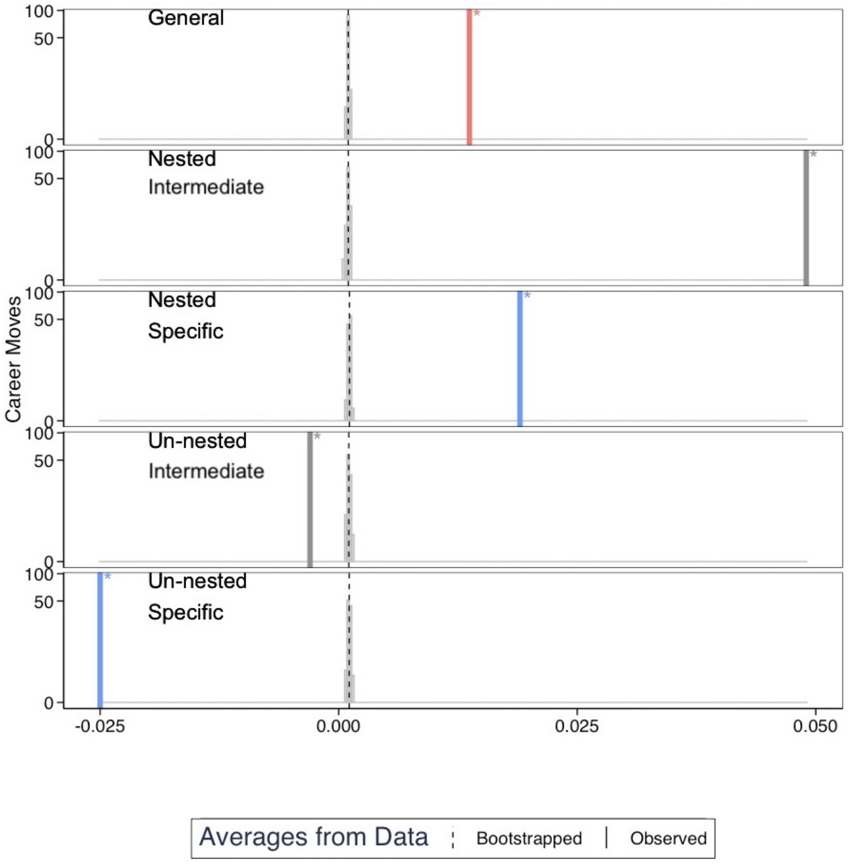

Supplementary Figure 26: Distribution Skill Changes from Bootstrapped Career Moves.

4.2 Occupational Median Age

Fig. 27 shows the trends of average skill levels and the average levels of the top 5 skills in each category against occupations' median age. This analysis supplements the main Fig. 4 (a-c).

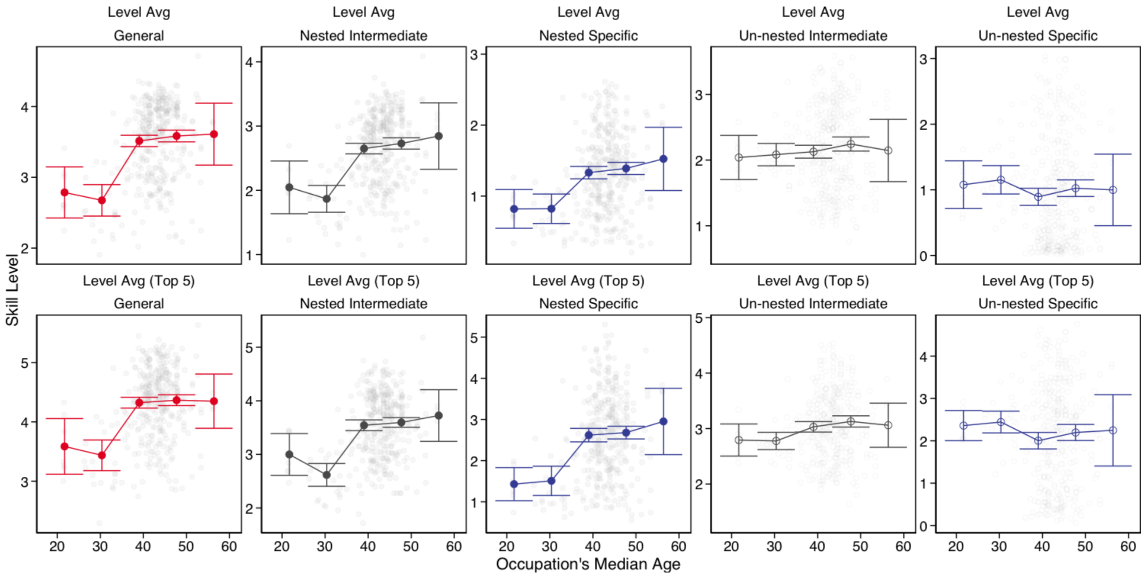

Supplementary Figure 27: Median Age of Workers in Occupation ($n = 542$)and Changes in Skill Categories.

4.3 Individuals' Age and Skills

Fig. 28 shows the trends of average skill levels and the average levels of the top 5 skills in each category as individuals age, accounting for the year effect. This analysis supplements the main Fig. 4 (d-f) by controlling for varying annual economic situations. The top 5 skills are determined based on the highest levels of skills in each category and are inferred for the individual based on their occupation. The results are consistent with the main figure.

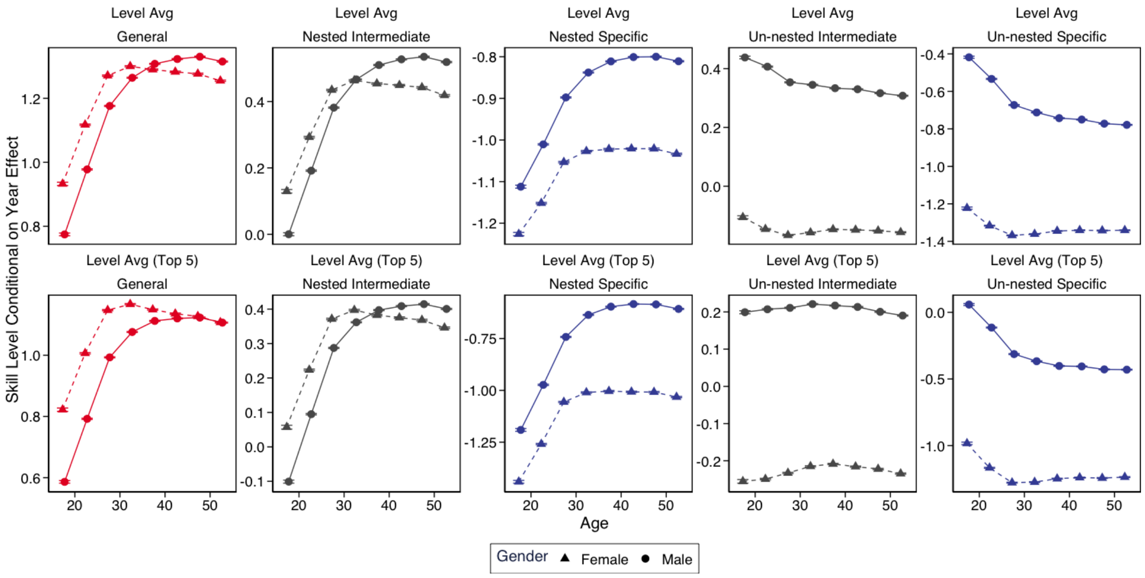

Supplementary Figure 28: Individuals' Skill Acquisition and Age ($n = 1,493,142$).

As robustness checks, we also show that the skill development observed in the main Fig. 4 continues long after education (Fig. 29) and also emerges for individuals without a college education (Fig. 30).

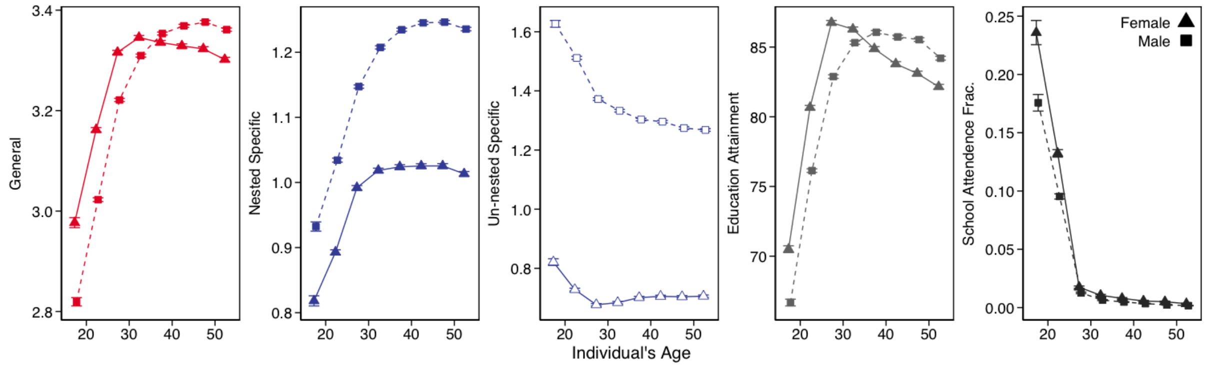

Supplementary Figure 29: Evolution of skill, age and education. To measure education, we have used educational attainment and the fraction of individuals who attend school as functions of age, both taken from the Current Population Survey (CPS). The education attainment variable ranges from 2 (i.e., no schooling) to 125 (i.e., doctorate degree). To obtain the fraction of the sample attending school, we utilized the information in the CPS variable SCHOOLCOL that documents attending high school (1 or 2) or college/university (3 or 4) or not attending school (5). We transformed the information so that if an individual attends school (1,2,3 or 4), it receives a value of 1, and if not attending, it has a value of 0. Even though by the age of 30, education plateaus and school attendance drops significantly, skill growth continues, manifesting the presence of other mechanisms for skill accumulation apart from education. Error bars show 95% CI ($n = 1,493,142$).

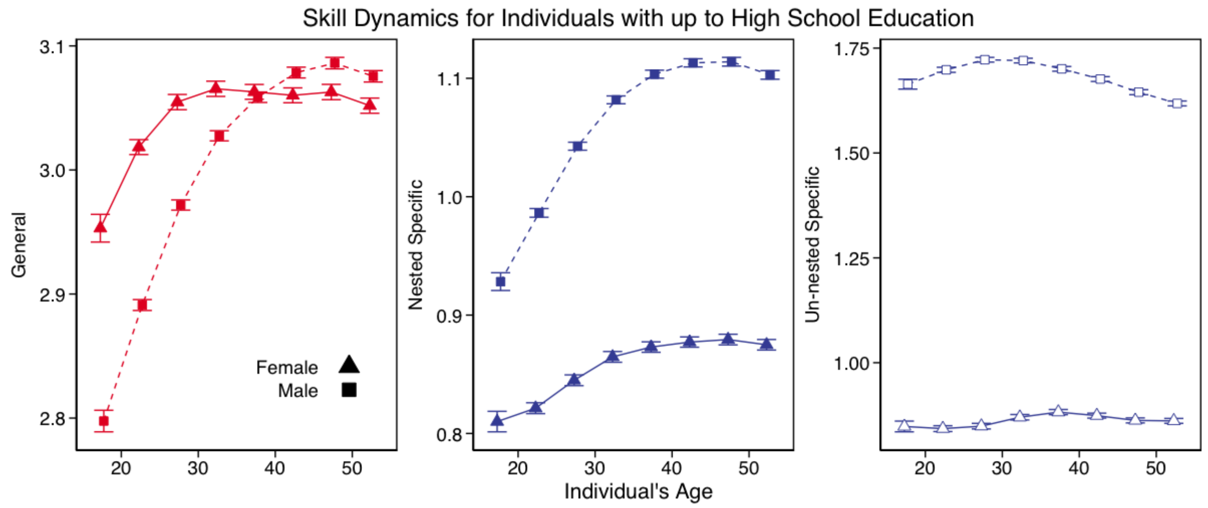

Supplementary Figure 30: Skill Acquisition and Age for Individuals with no College Education. The figure replicated the skill-age analysis (Fig. 4) for the subset of individuals who have obtained no more than a high school diploma (values of less than or equal to 073 on the CPS education attainment variable.) The patterns resemble the skill accumulation across the population, even though the levels of general and nested skills are lower compared to the population-level estimates. Error bars show 95% CI ($n = 715,318$).

5 Skill Investment and Payoffs

5.1 Investment and Payoffs of Skill Subtypes

Figure 31 shows "wage curves" that depict wages as a function of age for individuals in the most nested and the most un-nested occupations. The figures capture entrapment due to un-nested skills. To obtain wage curves, we averaged over the levels of nested and un-nested skills of each occupation in our sample. We picked occupations at the top 20% of the nested skills as the most nested, and occupations at the top 20% of the un-nested skills as the most un-nested. Matching these occupations to the individuals in the CPS, we can obtain estimates of wages for individuals in these occupations at different ages. To avoid conflating long-run economic factors, we show the wage-age curves for four distinct periods of 5-years: 1983-1987, 1993-1997, 2003-2007, 2013-2017. In three of the four periods, un-nested jobs have an early wage lead, which quickly evaporates with age. The pattern is consistent with the notion that learning is steeper in occupations with more complex tasks [22, 23]. To arrive at a complete picture, one would need to account for the higher cost of education associated with nested occupations. Hence, the wage offsets observed in the figure may occur later in individuals' lives in terms of real earnings once the cost of education is accounted for.

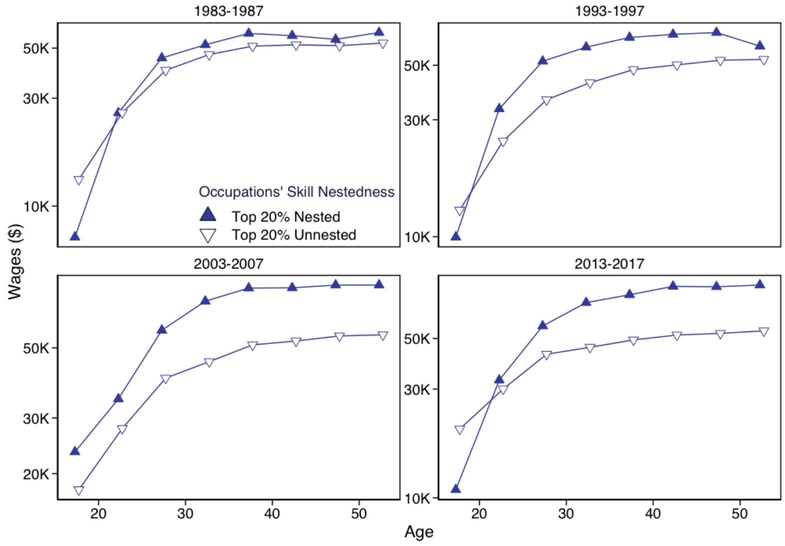

Supplementary Figure 31: Wage Curves for Occupations with Primarily Nested vs. Primarily Un-nested Skills. We average over the levels of nested and un-nested skills of each occupation in our sample and pick occupations at the top 20% of the nested skills as the most nested and occupations at the top 20% of the un-nested skills as the most un-nested. Matching these occupations to the individuals in the CPS, we can obtain estimates of wages for individuals in these occupations at different ages. To avoid conflating long-run economic factors, we show the wage-age curves for four 5-year periods: 1983-1987, 1993-1997, 2003-2007, 2013-2017. Un-nested jobs have an early wage lead which quickly evaporates with age.

Figures 32-34 capture a similar analysis to the main Fig. 5 for all skill subtypes, separating the relationship between skills and occupation educational requirement, occupation workplace experience, and wages, respectively. In each figure, the upper panel depicts the bivariate relationship between each nested or un-nested and intermediate or specific skill subset and a corresponding work measure (educational requirement, workplace experience, and wages). The inset shows the relationship between general skills. The lower panels control for general skills when regressing the work measure on the corresponding skill subset. The residualized form shows the partial association between the skill subtype and work measure. For nested skills,

the relationship with education weakens but for experience and wages it almost disappears, consistent with the main text and our intuition that general skills derive a large part of the signal. For un-nested skills, the predominantly negative relationships reverse to modest positive, consistent with the intuition that cetris paribus, un-nested skills behave as if human capital. Hence, they require training, accumulate experience, and contribute to wages, albeit modestly.

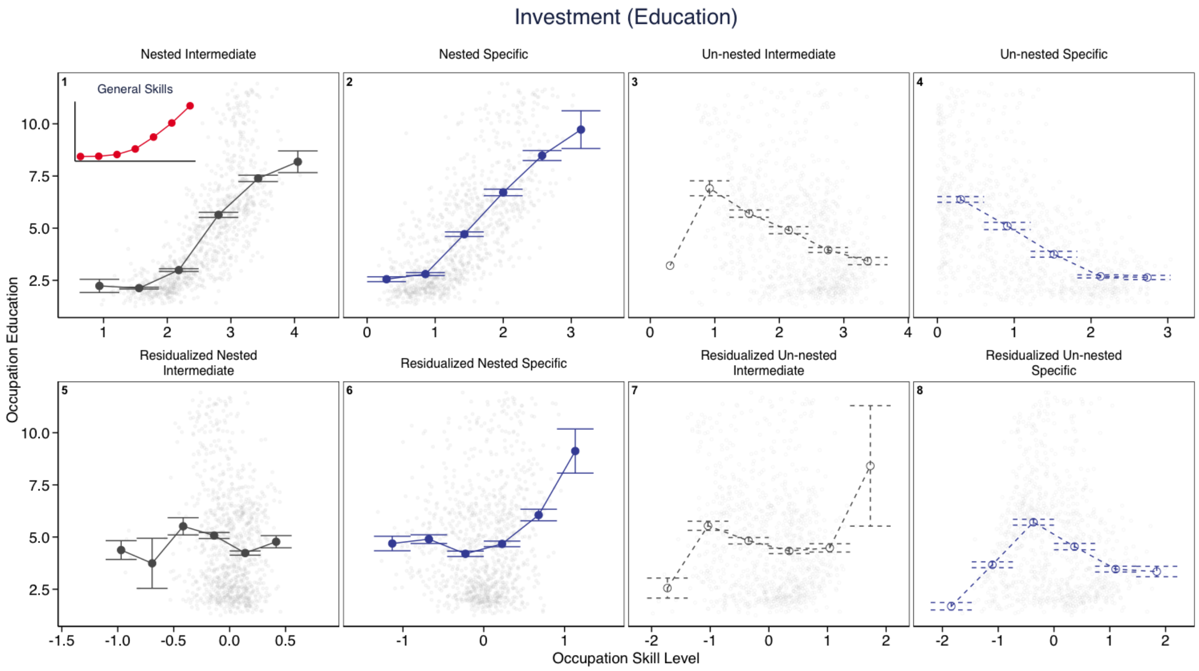

Supplementary Figure 32: Occupations' Educational Requirement against Skill Subtypes. Error bars show 95% CI ($n = 969$).

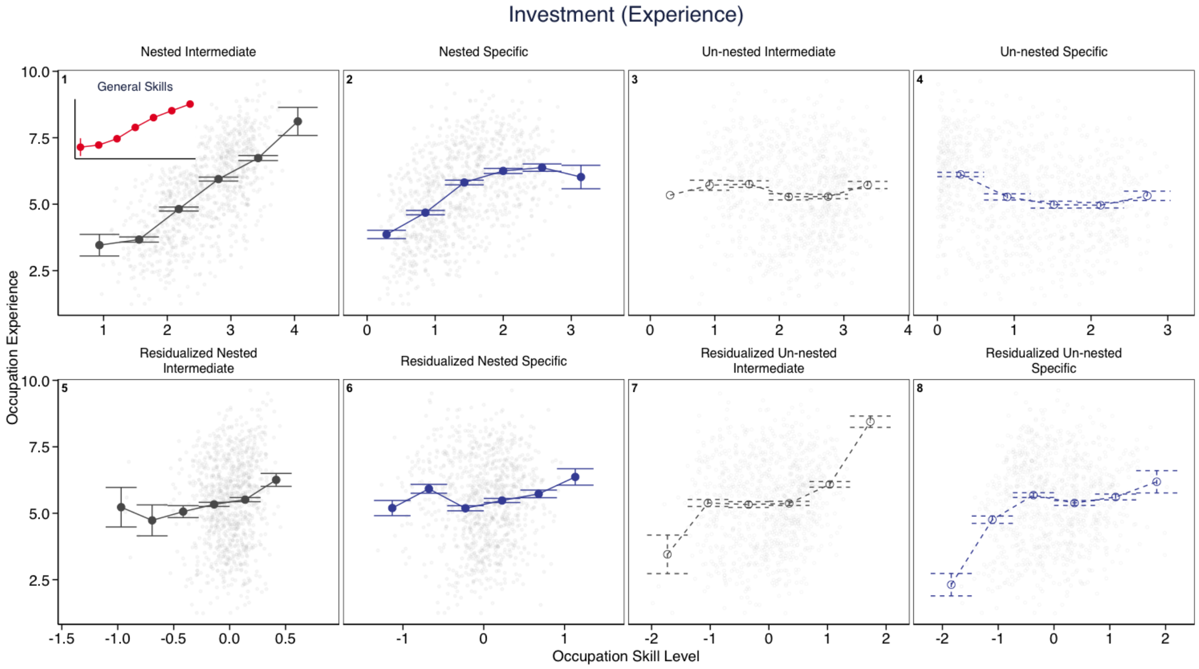

Supplementary Figure 33: Occupations' Workplace Experience against Skill Subtypes. Error bars show 95% CI ($n = 969$).

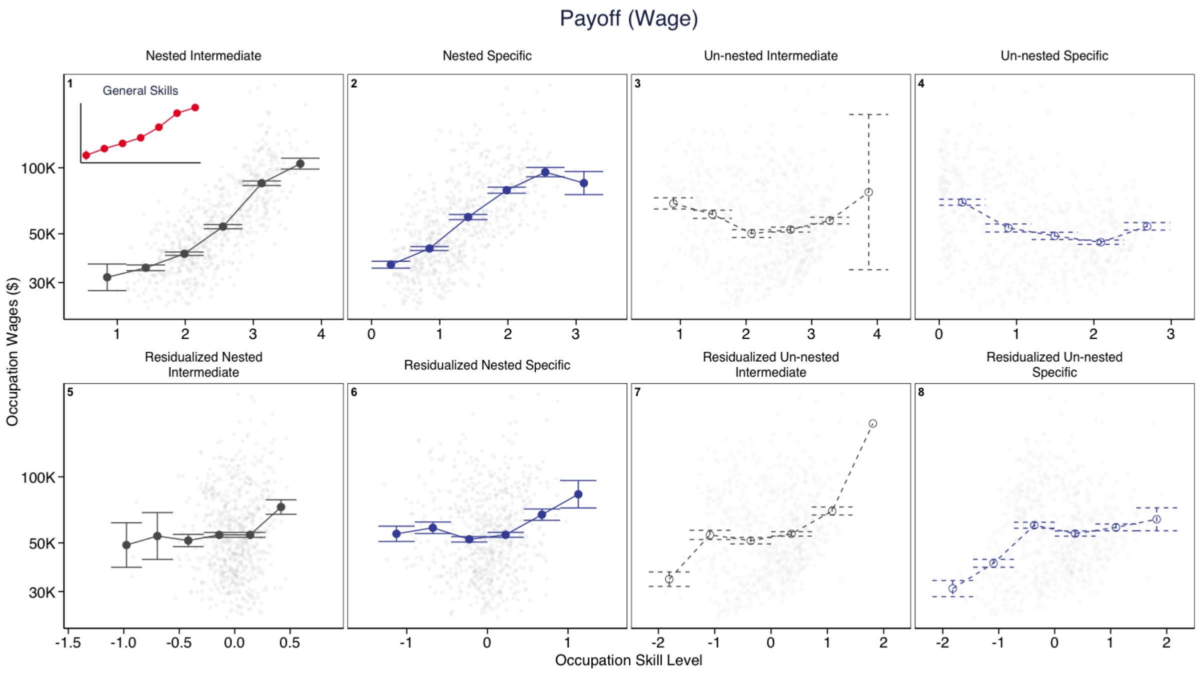

Supplementary Figure 34: Occupational Wages against Skill Subtypes. Error bars show 95% CI ($n = 774$).

Figs. 35-37 repeat the above analyses with the minor difference that the skill level is calculated not as the average of all skills that belong to a subtype, but as the average level of each occupations' top 5 skills in each skill category. The nature of the relationships is robust to this change— while slopes vary modestly.

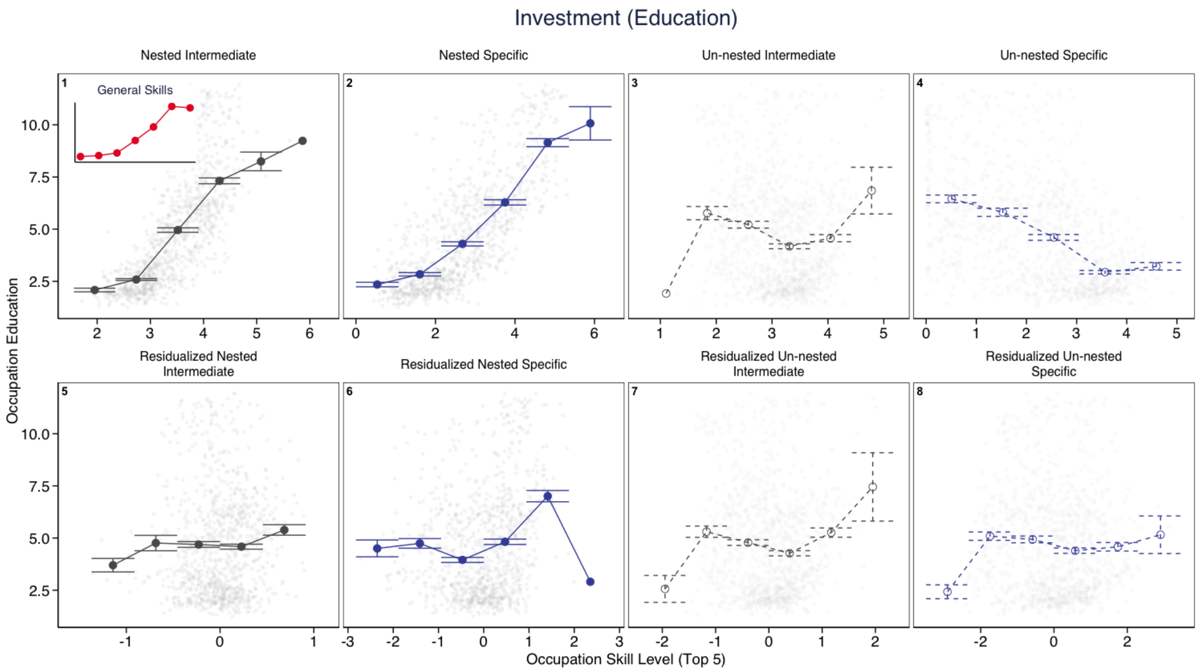

Supplementary Figure 35: Educational Requirement against Occupation's Top 5 Skills in Subtypes. Error bars show 95% CI ($n = 969$).

Table 5 supplements previous figures by comparing the partial effect of nested and un-nested categories for each skill group on wages. It also introduces conventional control vari-

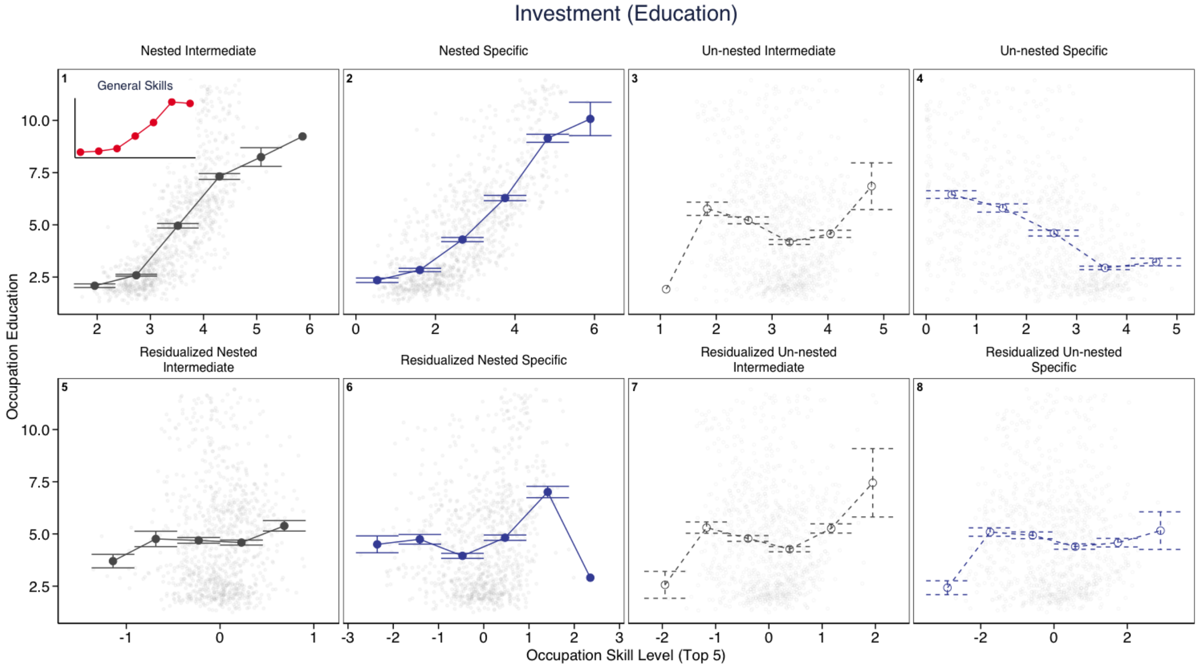

Supplementary Figure 36: Workplace Experience against Occupation's Top 5 Skills in Subtypes Error bars show 95% CI ($n = 969$).

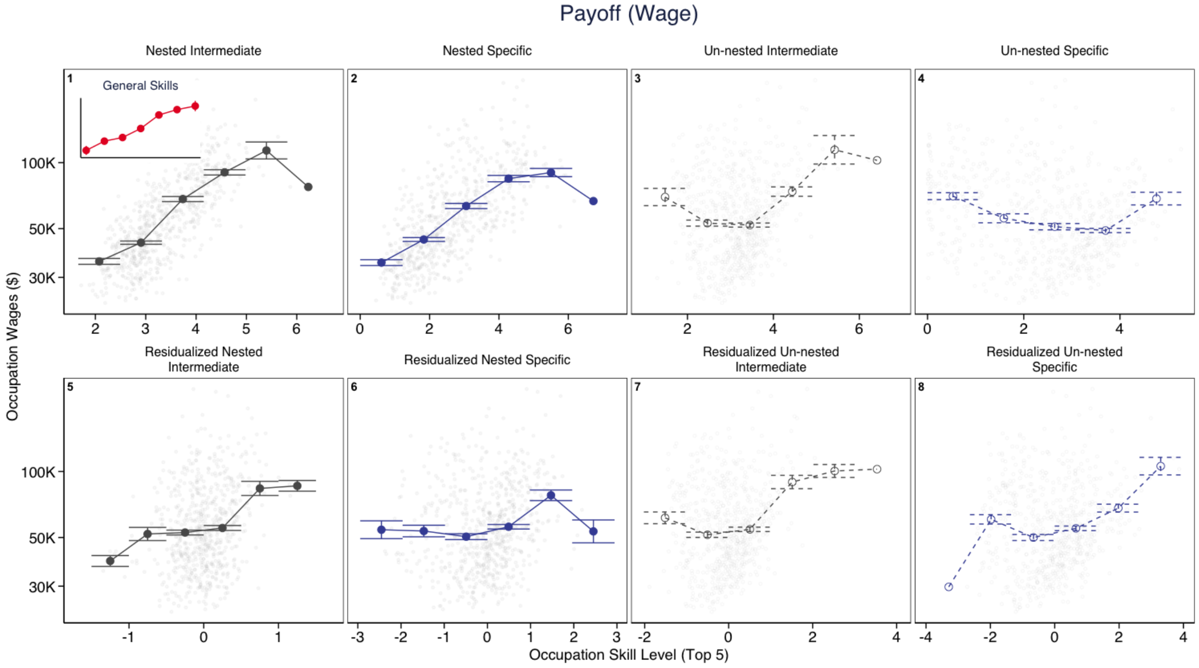

Supplementary Figure 37: Wages and Occupation's Top 5 Skills in Subtypes. Error bars show 95% CI ($n = 774$)

ables of human capital, such as education, experience, and training. The slopes are consistent with previous results and are robust (both statistically and in magnitude) to adding human capital controls. Note that we do not run a regression including all subtypes because of the biases introduced by adding pre-treatment variables— general skills are prerequisites to nested skills.

Supplementary Table 5: Wage Regression on Skill Endowment. OLS is used to estimate each regression.

	Dependent variable:							
	Log(Mean Annual Wage)							
	(1)	(2)	(3)	(4)	(5)	(6)	(7)	(8)
General	0.251			0.197	0.135			
	$p < 0.001$			$p < 0.001$	$p < 0.001$			
	(0.237,0.264)			(0.148,0.245)	(0.109,0.161)			
$Intermediate^{Nested}$		0.261		0.090		0.128		
		$p < 0.001$		$p < 0.001$		$p < 0.001$		
		(0.247,0.276)		(0.045,0.135)		(0.106,0.150)		
$Intermediate^{Unnested}$		−0.003		0.018		0.025		
		$p = 0.642$		$p = 0.211$		$p = 0.001$		
		(-0.016,0.010)		(-0.010,0.047)		(0.010,0.039)		
$Specific^{Nested}$			0.199	−0.009			0.042	
			$p < 0.001$	$p = 0.487$			$p < 0.001$	
			(0.182,0.215)	(-0.033,0.016)			(0.020,0.064)	
$Specific^{Unnested}$			−0.051	0.017			0.003	
			$p < 0.001$	$p = 0.168$			$p = 0.660$	
			(-0.063,-0.039)	(-0.007,0.042)			(-0.011,0.017)	
Education					0.024	0.033	0.042	0.049
					$p < 0.001$	$p < 0.001$	$p < 0.001$	$p < 0.001$
					(0.018,0.030)	(0.027,0.038)	(0.036,0.048)	(0.045,0.052)
Experience					0.014	0.013	0.023	0.023
					$p < 0.001$	$p < 0.001$	$p < 0.001$	$p < 0.001$
					(0.008,0.021)	(0.007,0.020)	(0.016,0.030)	(0.017,0.030)
Training					0.038	0.027	0.042	0.048
					$p < 0.001$	$p < 0.001$	$p < 0.001$	$p < 0.001$
					(0.029,0.048)	(0.017,0.037)	(0.031,0.052)	(0.039,0.058)
Constant	3.909	4.109	4.550	3.814	3.969	4.058	4.223	4.227
	$p < 0.001$	$p < 0.001$	$p < 0.001$	$p < 0.001$	$p < 0.001$	$p < 0.001$	$p < 0.001$	$p < 0.001$
	(3.861,3.956)	(4.058,4.160)	(4.520,4.581)	(3.716,3.912)	(3.908,4.031)	(4.011,4.104)	(4.182,4.264)	(4.190,4.265)
Observations	789	789	789	789	789	789	789	789
R^2	0.622	0.623	0.470	0.653	0.686	0.705	0.653	0.645
Adjusted R^2	0.622	0.622	0.469	0.651	0.684	0.703	0.651	0.643
Residual Std. Error	0.119 (df = 787)	0.119 (df = 786)	0.142 (df = 786)	0.115 (df = 783)	0.109 (df = 784)	0.106 (df = 783)	0.115 (df = 783)	0.116 (df = 785)
F Statistic	1,297.040*** (df = 1; 787)	649.424*** (df = 2; 786)	348.362*** (df = 2; 786)	294.629*** (df = 5; 783)	428.084*** (df = 4; 784)	374.552*** (df = 5; 783)	294.761*** (df = 5; 783)	475.021*** (df = 3; 785)

Note: OLS regressions are shown, with 95-percentile CI in parentheses. R^2, coefficient of determination, and adjusted R^2 is normalized for the models' number of variables. The P value is derived from a two-sided t-test.

Supplementary Table 6: Averages and Confidence Intervals on Trend Lines of Fig. 5 (a-b).

	Skills	Bins	Wage Averages	Wage 95% CI	Education	Education 95% CI
1	General Skills	1	0.65	(0.64, 0.65)	1.76	(1.48, 2.05)
2		2	0.66	(0.66, 0.66)	2.12	(2.06, 2.19)
3		3	0.67	(0.67, 0.67)	3.15	(3.03, 3.27)
4		4	0.69	(0.69, 0.69)	6.00	(5.82, 6.18)
5		5	0.70	(0.7, 0.7)	9.43	(9.05, 9.81)
6	Nested Specific Skills	1	0.66	(0.66, 0.66)	2.59	(2.46, 2.72)
7		2	0.67	(0.67, 0.67)	2.98	(2.9, 3.05)
8		3	0.68	(0.68, 0.68)	4.95	(4.84, 5.06)
9		4	0.69	(0.69, 0.69)	6.96	(6.81, 7.12)
10		5	0.70	(0.7, 0.7)	9.05	(8.75, 9.35)
11	Un-nested Specific Skills	1	0.68	(0.68, 0.69)	6.38	(6.24, 6.51)
12		2	0.67	(0.67, 0.68)	5.11	(4.93, 5.28)
13		3	0.67	(0.67, 0.67)	3.75	(3.61, 3.89)
14		4	0.67	(0.67, 0.67)	2.69	(2.62, 2.76)
15		5	0.68	(0.67, 0.68)	2.64	(2.54, 2.74)

Note: These are binned averages and confidence intervals. As no inference is made, no p-value is available.

Supplementary Table 7: Full Statistics on Regressions of Fig. 5. OLS is used to estimate each regression.

	Dependent variable:							
	Education			Log(Mean Annual Wage)				
	(1)	(2)	(3)	(4)	(5)	(6)	(7)	(8)
'Nested Specific Skills'	3.145			0.879	0.217			0.045
	$p < 0.001$			$p < 0.001$	$p < 0.001$			$p = 0.0004$
	(2.961,3.329)			(0.659,1.099)	(0.198,0.236)			(0.021,0.069)
'Un-nested Specific Skills'		−1.850	−0.505			−0.076	0.048	
		$p < 0.001$	$p < 0.001$			$p < 0.001$	$p < 0.001$	
		(-2.014,-1.685)	(-0.631,-0.379)			(-0.093,-0.059)	(0.035,0.061)	
General Skills			3.209	2.914			0.283	0.213
			$p < 0.001$	$p < 0.001$			$p < 0.001$	$p < 0.001$
			(3.043,3.376)	(2.697,3.131)			(0.266,0.301)	(0.191,0.236)
Constant	0.343	6.753	−5.747	−6.512	4.459	4.831	3.739	3.970
	$p = 0.016$	$p < 0.001$	$p < 0.001$	$p < 0.001$	$p < 0.001$	$p < 0.001$	$p < 0.001$	$p < 0.001$
	(0.065,0.620)	(6.527,6.980)	(-6.413,-5.081)	(-7.064,-5.959)	(4.431,4.486)	(4.807,4.856)	(3.669,3.808)	(3.913,4.027)
Observations	967	967	967	967	663	663	663	663
R^2	0.537	0.335	0.731	0.731	0.431	0.103	0.642	0.622
Adjusted R^2	0.537	0.334	0.730	0.730	0.430	0.102	0.641	0.621
Residual Std. Error	1.754 (df = 965)	2.104 (df = 965)	1.339 (df = 964)	1.339 (df = 964)	0.146 (df = 661)	0.184 (df = 661)	0.116 (df = 660)	0.119 (df = 660)
F Statistic	1,121.307*** (df = 1; 965)	485.073*** (df = 1; 965)	1,309.288*** (df = 2; 964)	1,308.798*** (df = 2; 964)	500.640*** (df = 1; 661)	76.188*** (df = 1; 661)	592.406*** (df = 2; 660)	543.638*** (df = 2; 660)

Note: OLS regressions are shown, with 95-percentile CI in parentheses. R^2, coefficient of determination, and adjusted R^2 is normalized for the models' number of variables. The P value is derived from a two-sided t-test.

5.2 Automation Risk and Skills

Given the broad interest in understanding human capital and automation risk, we plot occupations' automation risk index [1] against their average levels in each skill category in Fig. 38.

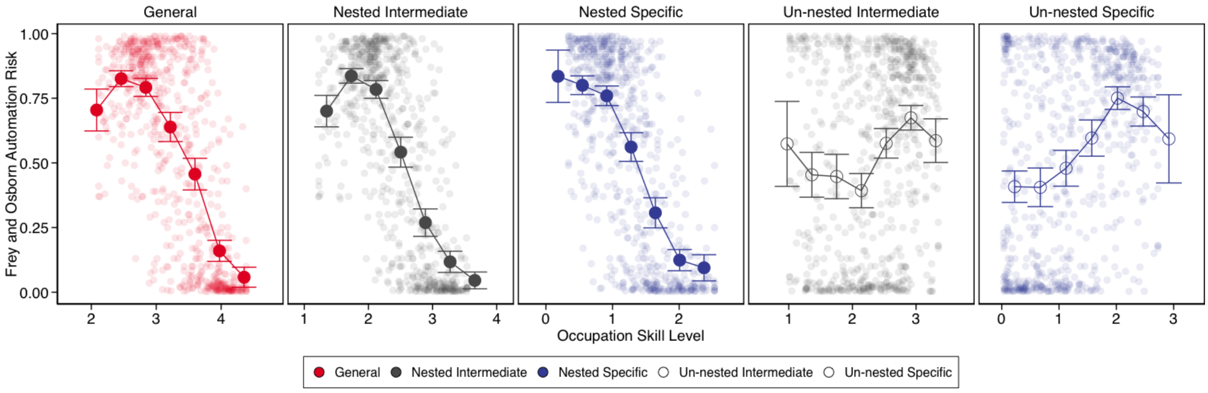

Supplementary Figure 38: Occupations' Automation Risk Index against their Average Levels in each Skill Category. Error bars show 95% CI ($n = 684$).

5.3 Skill Payoffs for Different Occupations

Fig. 39 relates returns to skills for each major occupational group— 1-digit SOC. The key pattern is that all occupational groups, despite varying in their skill endowments, benefit from higher levels of nested skills. However, un-nested skills only improve wages of Professional occupations and Skilled traders.

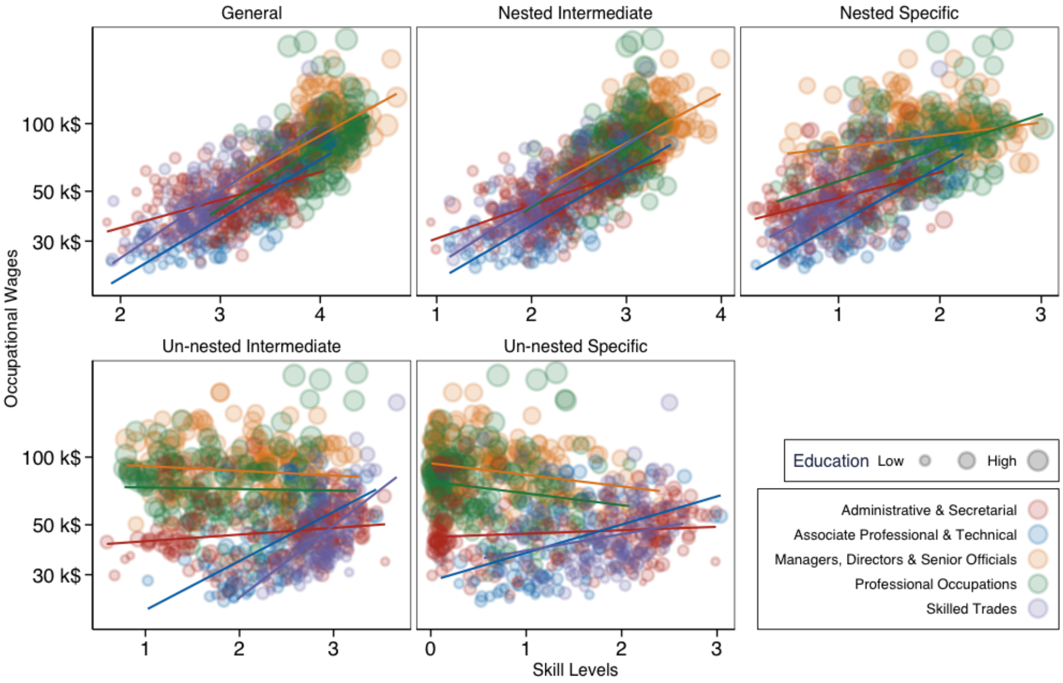

Supplementary Figure 39: Wage Returns to Different Types of Skill Endowment for each Major Occupational Group. Each point corresponds to an occupation. The setup supplements the main Fig. 5, highlighting the benefits of higher levels of nested skills. However, un-nested skills only improve wages of Professional occupations and Skilled traders. This in itself underpins multi-dimensionality skills.

Interestingly, managerial occupations command high general skills. Section 9.1 of the supplementary document examines (and finds evidence against) the possibility that the returns to general skills are largely a managerial phenomenon.

5.4 Skill Investment and Payoffs in 2005

In Fig. 40, we repeat our analysis of investment and payoffs to skills (main Fig. 5) for 2005, finding results consistent with the growing importance of general skills. The figures show lower associated education and payoffs to general skills than the main figure (5).

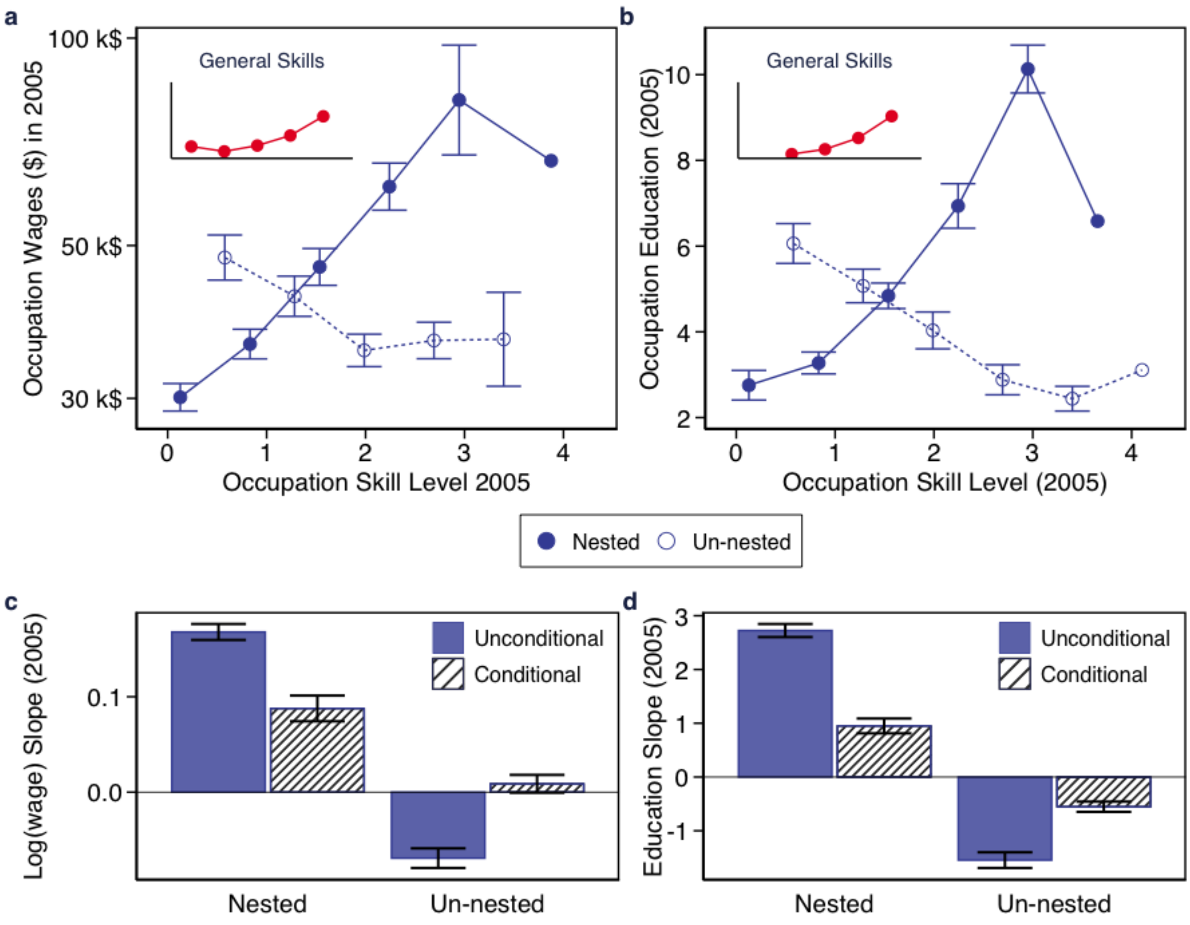

Supplementary Figure 40: Investment and Payoffs of Different Specific Skills in 2005. The figure repeats the analysis of investment and payoffs to skills in the main Fig. 5 for 2005 ($n_{(c)} = 707$ and $n_{(d)} = 480$), follows the presentation of the main Fig. 5, and finds results consistent with the growing importance of general skills. Error bars in a and b report 95% CI, and regression standard errors in c and d.

6 Skills' Geographic Distribution

An in-depth analysis of how skills interface with urban growth is beyond the scope of this work, we provide a brief descriptive analysis, here. Overall, urban areas are more endowed with general skills. In contrast, rural areas are less likely to carry general skills. This is consistent with the concentration of more innovative and complex economic activity [24, 25] and the concentration of managerial and administrative occupations in larger cities. We test and find support for the hypothesis that skills, in particular concentration of general skills, explain away part of the urban wage premiums. Upon grouping cities by manufacturing employment relative to the national average, we find that cities highly specialized in manufacturing tend to exhibit lower levels of nested specialization but higher levels of unnested specializations (Fig. 47). This shows that cities indeed specialize in distinct directions. Interestingly, skill patterns shift in a non-linear fashion across cities with increasing concentrations of manufacturing employment. Both a strong dependence on and a complete absence of manufacturing correlate with adverse skill bases, i.e., skill bases dominated by unnested skills and a lower prevalence of general and nested skills. Conversely, skills that typically command high wage premiums are overrepresented in cities with intermediate levels of manufacturing activity.

6.1 Counties' Skill Endowments

Using the occupational employment for Metropolitan and nonmetropolitan areas[1] published by the Bureau of Labor Statistics (BLS), one can map the geographical distribution of skills. BLS uses Core-based Statistical Areas (CSAs) as geographic units, which are more coarse than the county level. US counties follow the Federal Information Processing System (FIPS) taxonomy. To obtain employment at the level of FIPS and map skill information onto US counties, we used a crosswalk also provided by BLS [2]. We aggregate occupation skills at the level of *general, nested intermediate and specific, and un-nested intermediate and specific*. Taking an average for each US county using the county employment of occupations as weights, we derive a regional measure of skill endowment for each skill sub-type.

Overall, our analysis (Figs. 41 through 45) show a clear concentration of general skills in densely populated urban areas, reflecting the diverse and complex economic activities found in these locales [24–31]. Large cities tend to have higher levels of general and nested skills (also seen in Fig. 46). For instance, New York and Washington D.C. harbor significant financial and state employment. Moreover, even in states with comparatively rural structures, such as Indiana, Iowa, Nebraska, and Kansans, state capitals, where the local state is likely to reside, command a higher level of general skills— than their neighboring counties. A secondary driver of the abundance of general skills in urban areas is the specialization needed for accomplishing complex economic tasks. For instance, Boston, Seattle, and San Francisco (the latter not shown on the map) are tech hubs and command a strong stock of general (, and as seen in Fig. 43, specific) skills[3]. However, the starkest disparities between smaller and larger cities are seen in the

[1] `https://www.bls.gov/oes/`

[2] `https://www.bls.gov/oes/current/msa_def.htm`

[3] Finer-grained insights can also be obtained from these maps. For instance, the most extreme concentration of general skills (or lack thereof) is observed in less populated cities that are specialized in a certain industry. The significant proportion of the focal industries' workers relative to the total employment highlights the skills used by those workers. The most extreme concentrations of general skills (or lack thereof) are observed in less populated cities that are specialized in a certain industry. The significant proportion of the focal industries' workers relative to the total employment highlights the skills used by those workers. The five most and least endowed counties with general skills are shown on the map— as italicized text. For instance, St. Mary County (Maryland) is an

Supplementary Table 8: General Skills Explain Urban Wage Premiums. OLS is used to estimate each model.

	Dependent var.: Log(Wage)			
	OLS			
	(1)	(2)	(3)	(4)
Population > 1M	0.082	0.054	0.056	0.059
	$p < 0.001$	$p < 0.001$	$p < 0.001$	$p < 0.001$
	(0.080,0.084)	(0.053,0.056)	(0.054,0.058)	(0.057,0.060)
General Skills		0.269		0.281
		$p < 0.001$		$p < 0.001$
		(0.268,0.270)		(0.278,0.283)
Nested Specific Skills			0.248	0.007
			$p < 0.001$	$p < 0.001$
			(0.246,0.250)	(0.005,0.010)
Un-nested Specific Skills			−0.073	0.026
			$p < 0.001$	$p < 0.001$
			(-0.074,-0.072)	(0.025,0.027)
Constant	4.671	3.787	4.471	3.709
	$p < 0.001$	$p < 0.001$	$p < 0.001$	$p < 0.001$
	(4.670,4.673)	(3.783,3.792)	(4.469,4.474)	(3.702,3.717)
Observations	635,554	635,554	635,554	635,554
R^2	0.012	0.200	0.141	0.203
Adjusted R^2	0.012	0.200	0.141	0.203
Residual Std. Error	0.368 (df = 635552)	0.331 (df = 635551)	0.343 (df = 635550)	0.331 (df = 635549)
F Statistic	7,845.032*** (df = 1; 635552)	79,439.180*** (df = 2; 635551)	34,872.520*** (df = 3; 635550)	40,451.410*** (df = 4; 635549)

Note: OLS regressions are shown, with 95-percentile CI in parentheses. R^2, coefficient of determination, and adjusted R^2 is normalized for the models' number of variables. The P value is derived from a two-sided t-test.

prevalence of unnested skills, which are significantly less common in cities with over a million inhabitants, a known threshold for cities transitioning towards innovative economic specializations [24]. While most workers with nested skills need general s kills, the concentration of managerial and other supporting roles also needs high levels of general skills. Hence, examine and find evidence consistent with the hypothesis that the accumulation of general skills indeed explains part of the value generated in large cities (Tab. 8).

6.2 Skills and Population

We divide cities into four mutually exclusive groups by population (below 10 thousand, below 50 thousand, below 1 million, and more than a million inhabitants) based on 2010 Census population estimates. Skill endowment for each city group is taken as the average of counties, and 95% confidence intervals are shown. Fig. 46 shows for cities of different size the levels of all skill categories.

We also test the hypothesis that the accumulation of general skills indeed explains part of the value generated in cities [29, 32, 33]. we test that hypothesis directly by utilizing the CBSA size (CBSASZ) variable from CPS microdata, which carries information about the size of the metropolitan area in which the surveyed individual resides (since 2004). The values range

air force and aerospace hub with companies such as Lockheed Martin and Boeing, and military naval air station Patuxent River among the top employers. Another example is Chatham and its neighboring counties, Durham (hosting Duke University), Orange (hosting the University of Carolina at Chapel-Hill), and Person, which have fostered one of the fastest growing tech sectors in the US, earning the nickname of *Research Triangle*. Other notable concentration points of general skills are Limestone and Madison (Alabama), hosting numerous aerospace and automobile manufacturing facilities, and Washtenaw county (Michigan) hosting the University of Michigan Ann Arbor and its off-sprung businesses. In contrast, Madera (California), and its neighboring counties, Tulare, Kings, and Monterey)Highlands (Florida), Yuma (Arizona), Hall (Georgia), and Kalawao (Hawaii) are primarily designated agricultural areas, accruing unnested skills.

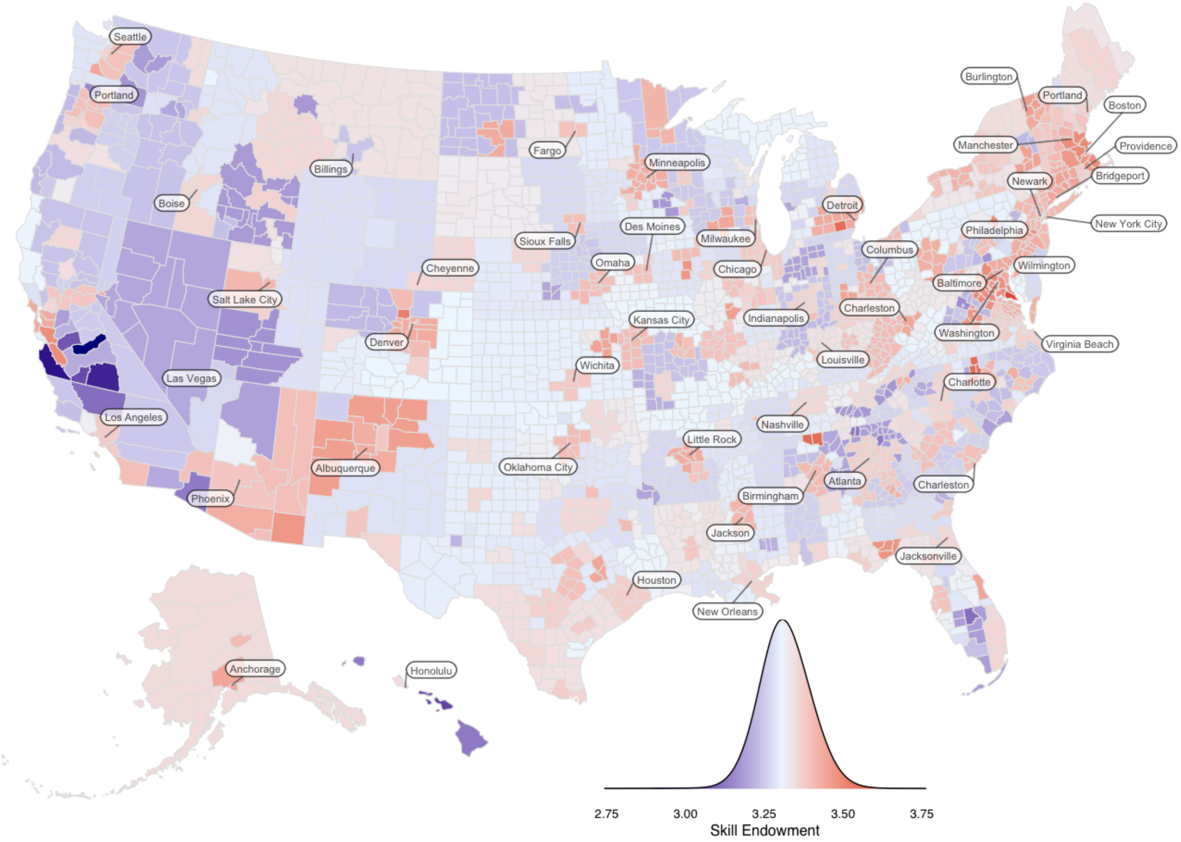

Supplementary Figure 41: Geographic Distribution of Average General Skills Weighted by Employment. The bottom histogram shows the distribution of the corresponding stock of skills across the US economy. The y-axis shows the number of unique FIPS with the respective skill level. Overall, urban areas are more endowed with general skills— seen in red. In contrast, rural areas are less likely to carry general skills— seen in blue. This is consistent with the concentration of more innovative and complex economic activity [24, 25] and the concentration of managerial and administrative occupations in cities. We use R package *usmap* (0.7.1) for the visualization.

from 0: areas of $< 100,000$ inhabitants that do not meet the threshold of a metropolitan area to 6: over 5 million inhabitants. We transform these brackets to cities below and above 1M population [24].

In the model (1) of Tab. 8, we first regressed the log wage reported by individuals to CPS on the size of the metropolitan area in which they reside, obtaining partial correlations that signify the urban wage premiums (the baseline is areas of $< 1M$ inhabitants.) In the second model of the table, we add general skills of individuals (which we obtain from matching to O*NET the occupation associated with each individual in the CPS microdata). That means that large cities tend to have more people in occupations with general skills. This bias toward more general-skill intensive activities explains over one-third of the urban wage premiums [31–34]. Adding nested and un-nested specific skills first without and then with general skills in models 3 and 4, respectively, have similar effects.

6.3 Skills and Manufacturing Industries

We divide cities into four mutually exclusive groups based on the intensity of their manufacturing industries. We use US Census County Business Patterns from 2019 that report industry employment for metropolitan areas to quantify manufacturing presence. At the 2-digit naics

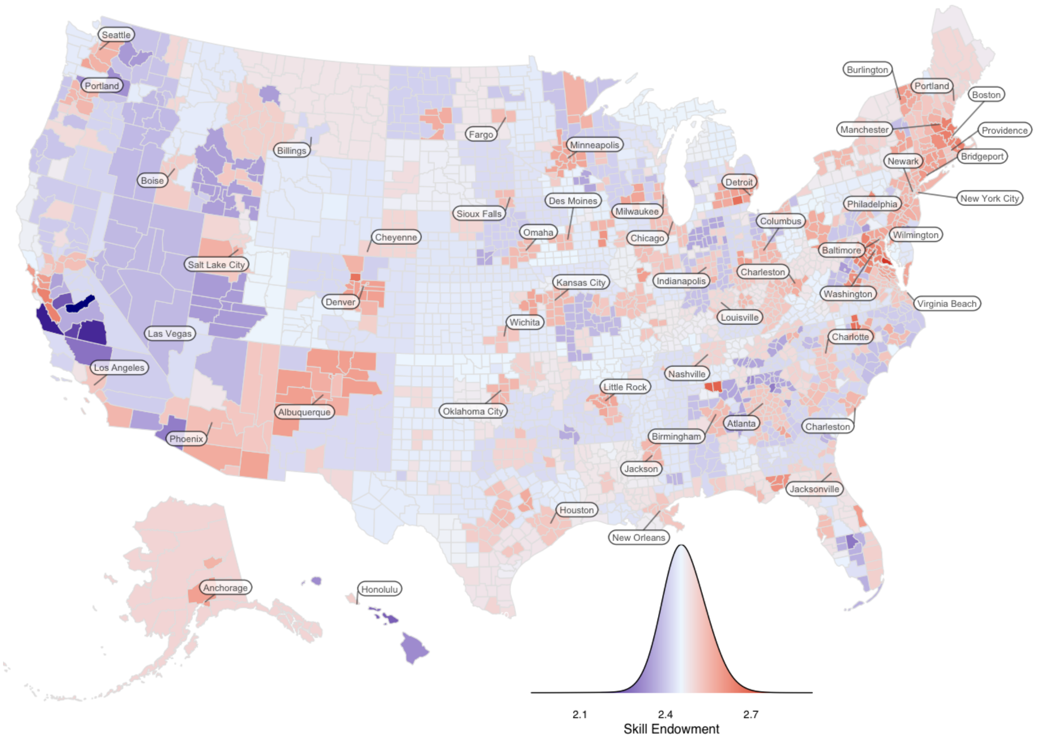

Supplementary Figure 42: Geographic Distribution of Average Nested Intermediate Skills Weighted by Employment. The bottom histogram shows the distribution of the corresponding stock of skills across the US economy. The y-axis shows the number of unique FIPS with the respective skill level. Based on R package *usmap*.

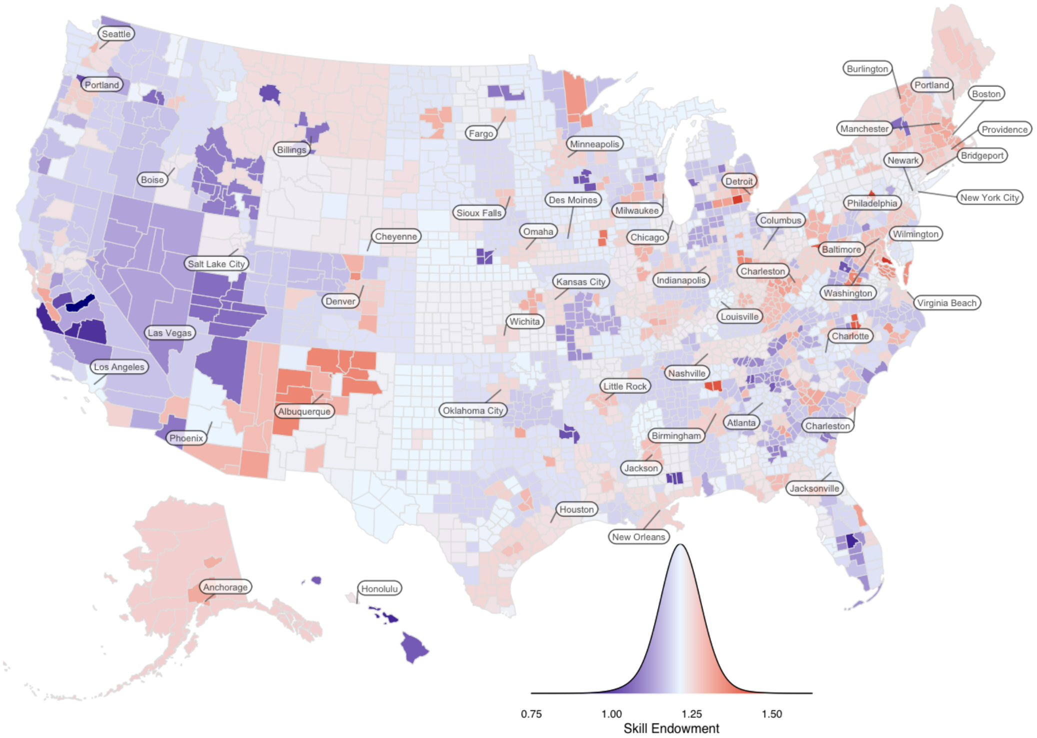

Supplementary Figure 43: Geographic Distribution of Average Nested Specific Skills Weighted by Employment. The bottom histogram shows the distribution of the corresponding stock of skills across the US economy. The y-axis shows the number of unique FIPS with the respective skill level. Based on R package *usmap*.

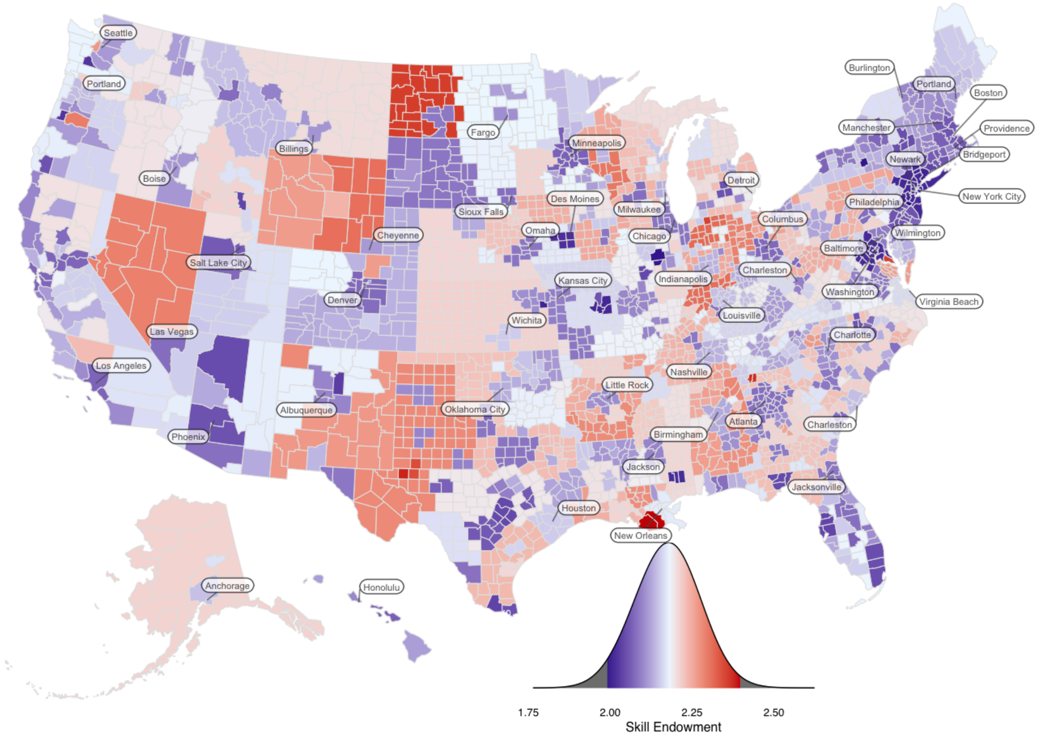

Supplementary Figure 44: Geographic Distribution of Average Un-nested Intermediate Skills Weighted by Employment. The bottom histogram shows the distribution of the corresponding stock of skills across the US economy. The y-axis shows the number of unique FIPS with the respective skill level. Based on R package *usmap*.

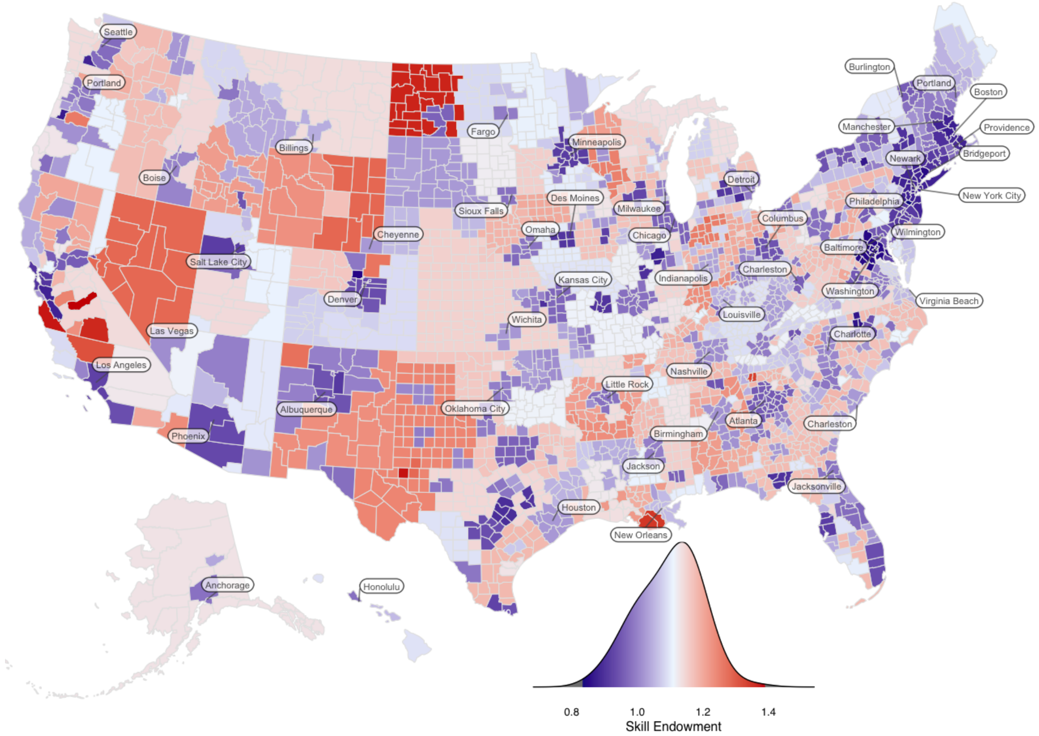

Supplementary Figure 45: Geographic Distribution of Average Un-nested Specific Skills Weighted by Employment. The bottom histogram shows the distribution of the corresponding stock of skills across the US economy. The y-axis shows the number of unique FIPS with the respective skill level. Based on R package *usmap*.

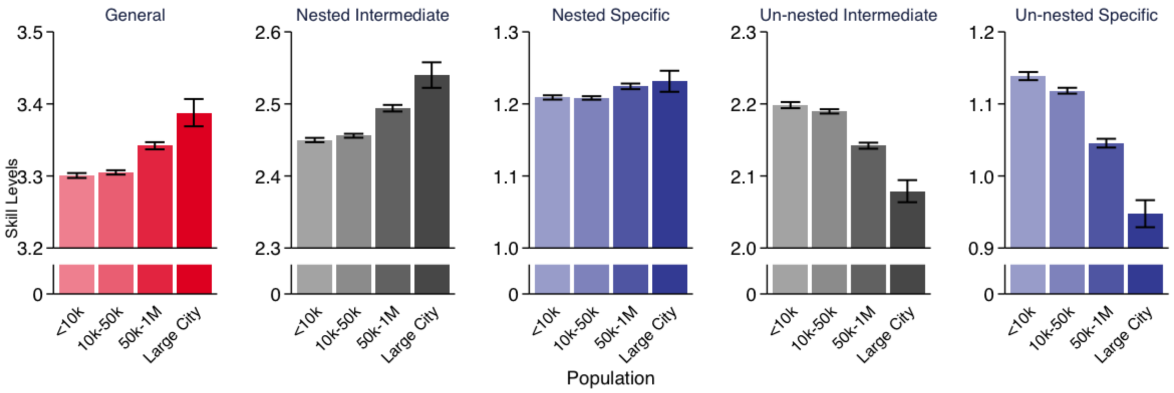

Supplementary Figure 46: Population Size and Skills The figure shows for cities of different size the levels of all our skill categories, highlighting the statements for nested and un-nested specific skills also hold for the corresponding intermediate skills. Error bars show 95% CI ($n = 3,142$).

codes, we take 31-33 as manufacturing industries and calculate the location quotient of manufacturing employment (the ratio of manufacturing employment from the metro area total employment over the nationwide ratio). Matching metro areas to counties, we designate counties with no manufacturing employment to group "None", and group the rest based on quotient 33% and 66% quantiles of the measure into bottom, middle, and top. Fig. 47 shows for cities of different manufacturing concentrations the levels of all skill categories.

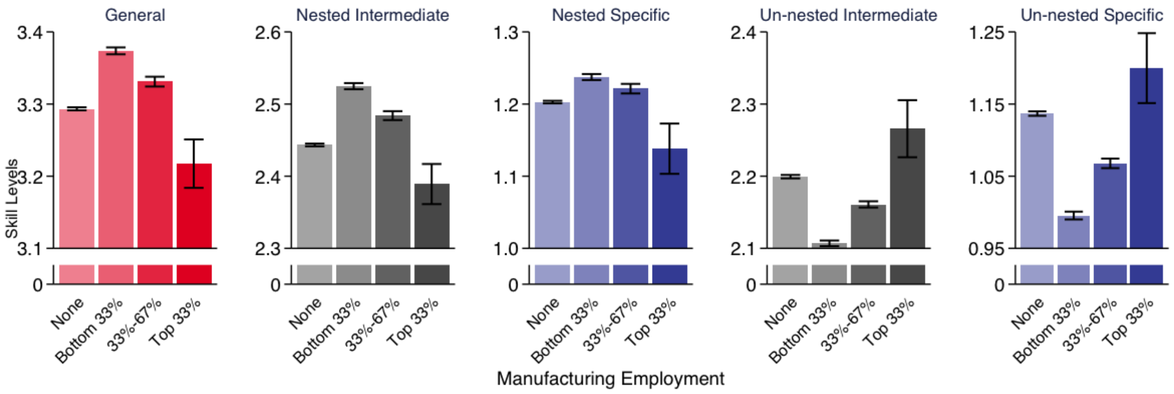

Supplementary Figure 47: Intensity of Manufacturing Industries and Skills The figure shows for cities of different manufacturing concentrations the levels of all skill categories, highlighting the statements for nested and un-nested specific skills hold also for the corresponding intermediate skills. Error bars show 95% CI ($n = 1,137$).

7 Skills' Demographic Distribution

Using the CPS household data between 1980 and 2022, we derive the skill endowment across racial (White, Black, Hispanic/Latinx, and White) and gender (Female and Male) groups in each skill category. Restricting to full-time workers employed at the time of the survey, who are between 18 and 55, we apply the mentioned features to examine the prevalence of skills among individuals of different gender and racial groups. Individuals' skills are infered based on their coded occupations in the CPS data by linking it to the occupational skill requirement in O*NET. The two datasets, however, use different occupational taxonomies. As a result, one needs to map CPS and O*NET occupations. We use a crosswalk offered by BLS[4], which maps a CPS occupation to 542 out of 968 occupations in O*NET 8-digit SOC codes. Note that CPS offers various racial categories. We use Whites, Blacks, and Asians, which constitute the bulk of the sample. CPS data also contains a separate (from race) variable for identifying Hispanic individuals. We create a fourth racial category for Hispanics and associate any individual of Hispanic background with that category. Next, we calculate the endowment of each skill category for each of the resulting four demographic categories and (binary) gender groups.

Figure 48 replicates the main Fig. 6, adding the information on intermediate skills and annual wage. We omit the weekly wage results for brevity.

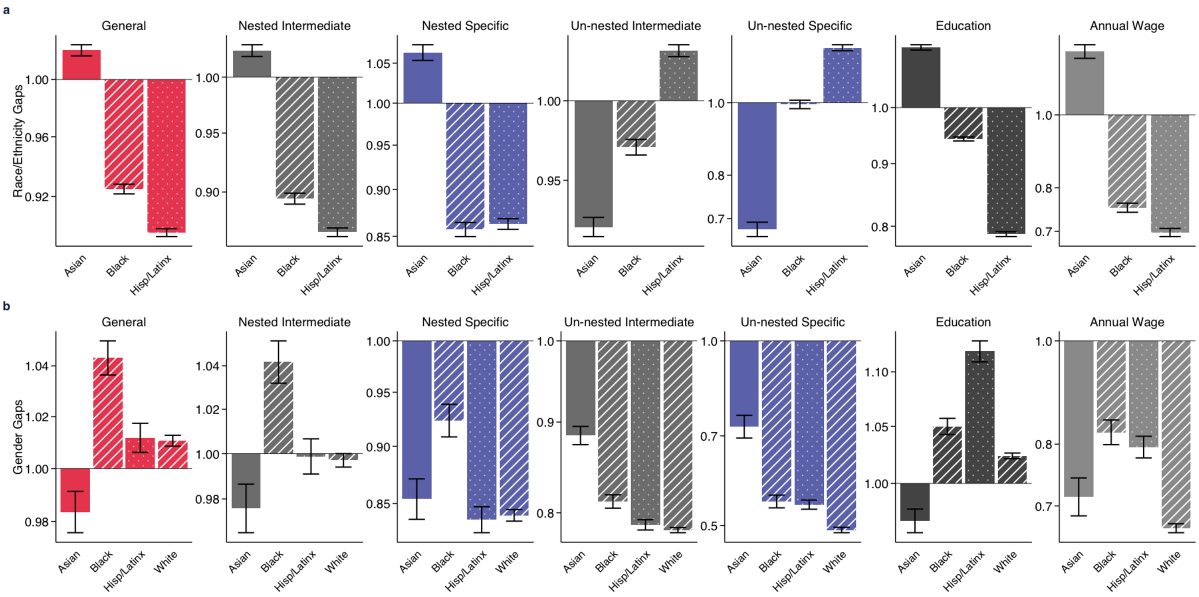

Supplementary Figure 48: Skill Disparity in Demographic Distribution of race/ethnicity and gender adding the information on intermediate skills and annual wage ($n_{Asian} = 86,055$, $n_{Black} = 180,304$, $n_{Hisp.} = 291,493$, $n_{White} = 1,375,617$, $n^{Fem}_{Asian} = 34,586$, $n^{Fem}_{Black} = 89,230$, $n^{Fem}_{Hisp.} = 105,371$, and $n^{Fem}_{White} = 518,688$). Error bars show 95% CI.

As a robustness check, we used a different measurement of skills for demographics and found similar results, following Tong et al. [35]. They group occupations of different skill levels by corresponding workers' dominant gender and race/ethnicity and calculate skill endowment across occupations from the same group. In determining occupations' "dominant" demographic characteristics, we link an occupation to a racial/gender group if it is 1.5 times or more likely to be employed in the focal occupation than its fraction in the sample. We then aggregated skill endowments across racial and gender categories and show the results in Fig. 49. The results are consistent with our main Fig. 6 and SI Fig. 48.

[4] https://www.census.gov/topics/employment/industry-occupation/guidance/code-lists.html

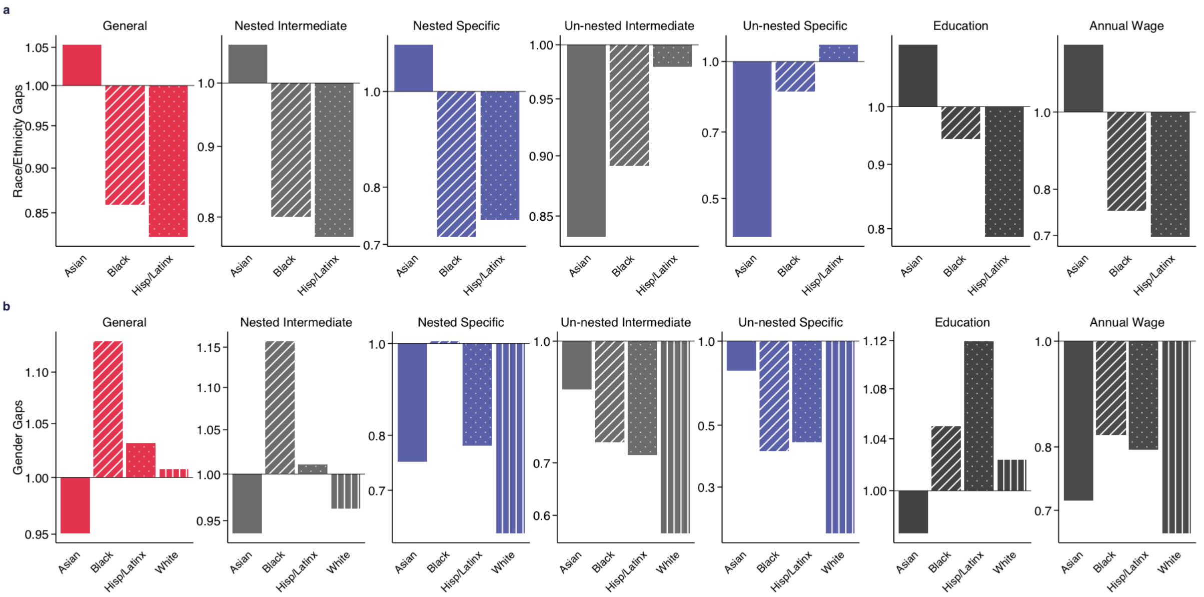

Supplementary Figure 49: Skill Disparity in Demographic Distribution of race/ethnicity and gender with an alternative aggregation. Similar to our main Fig. 6, we use CPS micro data, however, follow the aggregation of [35]. The results are consistent with our main figure ($n_{Asian} = 86,055$, $n_{Black} = 180,304$, $n_{Hisp.} = 291,493$, $n_{White} = 1,375,617$, $n^{Fem}_{Asian} = 34,586$, $n^{Fem}_{Black} = 89,230$, $n^{Fem}_{Hisp.} = 105,371$, and $n^{Fem}_{White} = 518,688$). Error bars show 95% CI.

7.1 Parenthood and the Diverging Skills of Male and Female Workers

An intriguing pattern in the main Figs. 4 (d-e) is the diverging general and nested skills of men and women around the age of 30, when one expects some individuals to become parents. Utilizing the number of children in the surveyed households recorded by CPS microdoa, we split our birth cohort sample into individuals *with* and *without* children. We replicate the analysis of the main Fig. 4 (d-f) by tracking the skills manifested in the occupational compositions of birth cohorts as they age, splitting individuals based on their binary gender (Male: lower panels; Female: upper panels of the below figure) and their binary parental status (with child: square; without child: triangle in the below figure) at the time of the survey. Fig. 50 shows the result of aggregating skills for each subgroup. Each column shows the levels of a certain category of skills, while the rows show the results for a gender. The solid line (and triangles) show the pattern for people without children, while the dashed line (and squares) show the pattern for individuals with children.

There is a pronounced gap in the general and nested skills between women with and without children. Please note that the later convergence is likely to arise from the fact that at higher ages, the "without" subgroup will mix families who never had any children with families whose children have already left the household. In the latter families, caregivers may have been disadvantaged in their early careers, leading to lower skill levels at higher ages. Contrary to the negative correlation with general and nested skills, women with children appear to sort into jobs that require higher un-nested skills (the SI Sec. 7.2 offers partial evidence of female job sorting). Interestingly, men with children tend to do better. Especially men in jobs that require general and nested skills tend to be more intriguing and for longer periods compared to their counterparts without children. The latter pattern for men may arise from sample selection effects or from the fact that the cost of raising children incentivizes acquiring skills that lead to better-paid careers. Synthetic birth cohorts are not ideal data for this purpose, as they do not allow for tracking individuals over time. However, it is reasonable to believe this approach

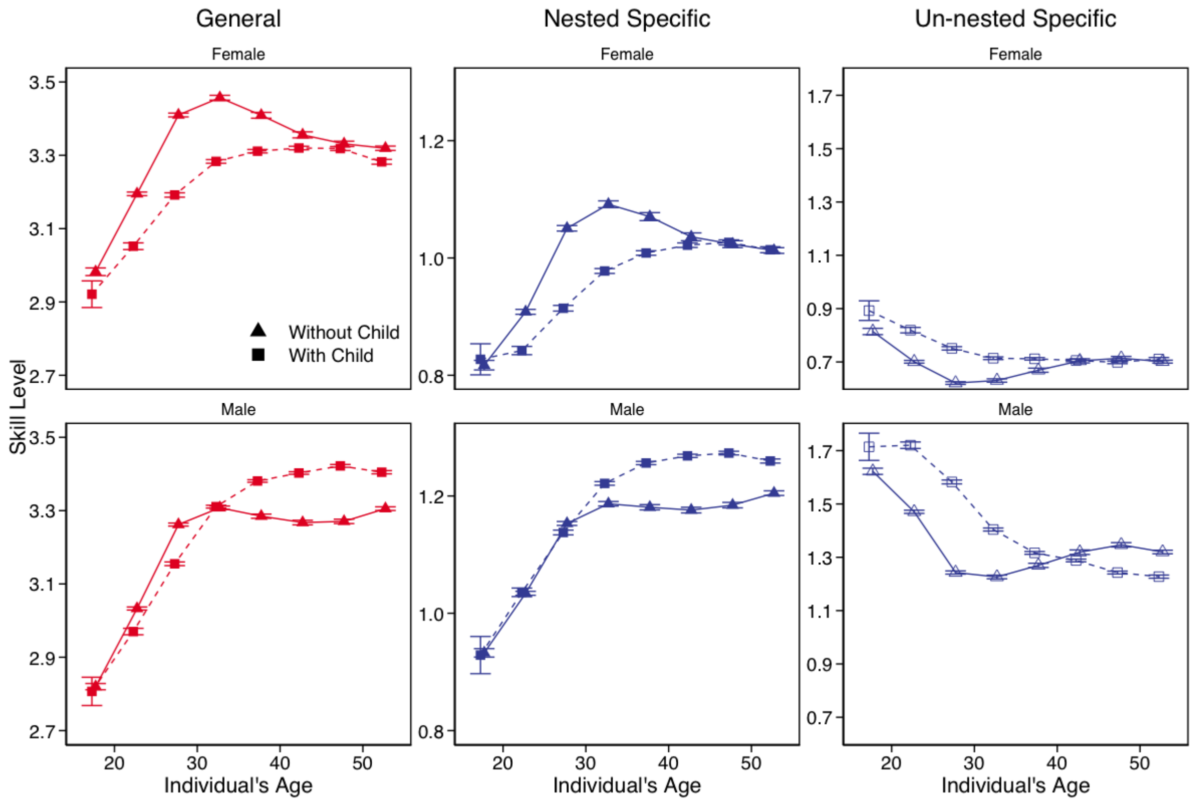

Supplementary Figure 50: Parenthood and the Diverging Skills of Male and Female Workers. We track the skills manifested in the occupational compositions of birth cohorts as they age, splitting individuals ($n = 1,493,142$) based on their binary gender (Male/Female) and whether they lived with children at the time of the survey, obtaining the below figure. Each column shows the levels of a certain category of skills, while the rows show the results for men and women. The solid line (and triangles) show the pattern for people without children, while the dashed line (and squares) show the pattern for individuals with children, highlighting the drop in general and nested skills of mothers. Error bars show 95% CI.

offers unbiased estimates of the population behavior.

7.2 Gender and Jobs Sorting

Another intriguing pattern in the main Fig. 4 (d-f) is the diverging patterns of skill development between men and women, wherein women exhibit high levels of general skills, surpassing their male counterparts at certain ages but do not manifest the high levels of nested skills observed for male workers of the same age. Lower levels of nested skills for women are also seen in the first column of the regression Tab. 9 that predicts the gender of workers based on their general and nested skills in our CPS sample (Female = 1): general skills are associated with greater, but nested skills with smaller shares of women in an occupation.

One explanation for this pattern [36–38] is that women may avoid jobs with irregular or long working schedules. This implies that despite their high levels of general skills and education, women may avoid jobs that require nested skills because of the working conditions of such jobs. To examine that hypothesis, we examined whether adding descriptors of work schedule to the same regression diminishes the correlation between skills and the gender of the worker, as reported in column 1 of Tab. 9.

To implement this test, we matched individuals in the Current Population Survey (CPS) aged 18 to 55 who were in the workforce between 1980 and 2022 to the following information

Supplementary Table 9: Regression analysis of the correlation between gender, skills, and irregular and long work schedule. The first column offers a baseline model that predicts the gender (Female = 1) of the worker based on general and nested skills, showing a negative correlation with nested skills. Adding descriptors of irregular and long schedules in the second model explains away part of the predictive power of nested skills for workers' gender. As such, part of the reason why women manifest high level s of general skills but comparatively low levels of nested skills is that jobs that require the latter categories of skills likely impose long and irregular work conditions, which have been found to deter female workers. OLS is used to estimate each regression.

	Dependent Var.: Gender Dummy (Female = 1)	
	OLS	
	(1)	(2)
General Skills	0.203	0.150
	$p < 0.001$	$p < 0.001$
	(0.201,0.204)	(0.148,0.153)
Nested Skills	−0.357	−0.258
	$p < 0.001$	$p < 0.001$
	(-0.359,-0.355)	(-0.261,-0.256)
Irregular Schedule		−0.338
		$p < 0.001$
		(-0.342,-0.334)
Long Hours Dummy (>50 weekly)		−0.176
		$p < 0.001$
		(-0.178,-0.174)
Constant	0.097	0.629
	$p < 0.001$	$p < 0.001$
	(0.092,0.101)	(0.620,0.638)
Observations	1,493,142	1,096,362
R^2	0.072	0.108
Adjusted R^2	0.072	0.108
Residual Std. Error	0.463 (df = 1493139)	0.455 (df = 1096357)
F Statistic	57,942.160*** (df = 2; 1493139)	33,058.290*** (df = 4; 1096357)

Note: OLS regressions are shown, with 95-percentile CI in parentheses. R^2, coefficient of determination, and adjusted R^2 is normalized for the models' number of variables. The P value is derived from a two-sided t-test.

in the O*NET using their reported occupation code: skill information (namely, general and nested specific skills) and occupational work schedule (irregularity). Work schedule irregularity is collected as a part of the O*NET work context record as a categorical variable with three levels of *Regular* (established routine, set schedule), *Irregular* (changes with weather conditions, production demands, or contract duration), and *Seasonal* (only during certain times of the year). This variable is reported for all occupations with weights associated with each category. For example, Chief Executive has the majority of weight in category 1, as it is primarily a job with a regular schedule. A surgeon has more weight, in comparison, on the irregular category. Using the weights, we obtained an aggregated "schedule irregularity" score for each occupation, wherein a value closer to 1 denotes a more regular schedule, and a value closer to 3 denotes a more irregular schedule. Next, to proxy *long working hours*, we follow Cha et al. [39] to use the number of hours worked during the week in the CPS data and form a dummy variable that is one if the worker had worked more than 50 hours a week, and 0 otherwise.

Adding the descriptors of irregular schedules or long hours (in Tab. 9 column 2) indeed diminishes the correlation estimated in the baseline, per the baseline model (column 1). A unit increase in the nested specific skills required by a job, decreases the chances of the worker being female by 36%. Adding schedule descriptors reduces that relation by more than one third, to about 26%.

7.3 Skills and Wage Gaps Have Narrowed Over Years

Figures 51 and 52 below show the temporal dynamics of skill, education, and wage gaps shown as averages in the main Fig. 6. These figures show the gaps have narrowed over years.

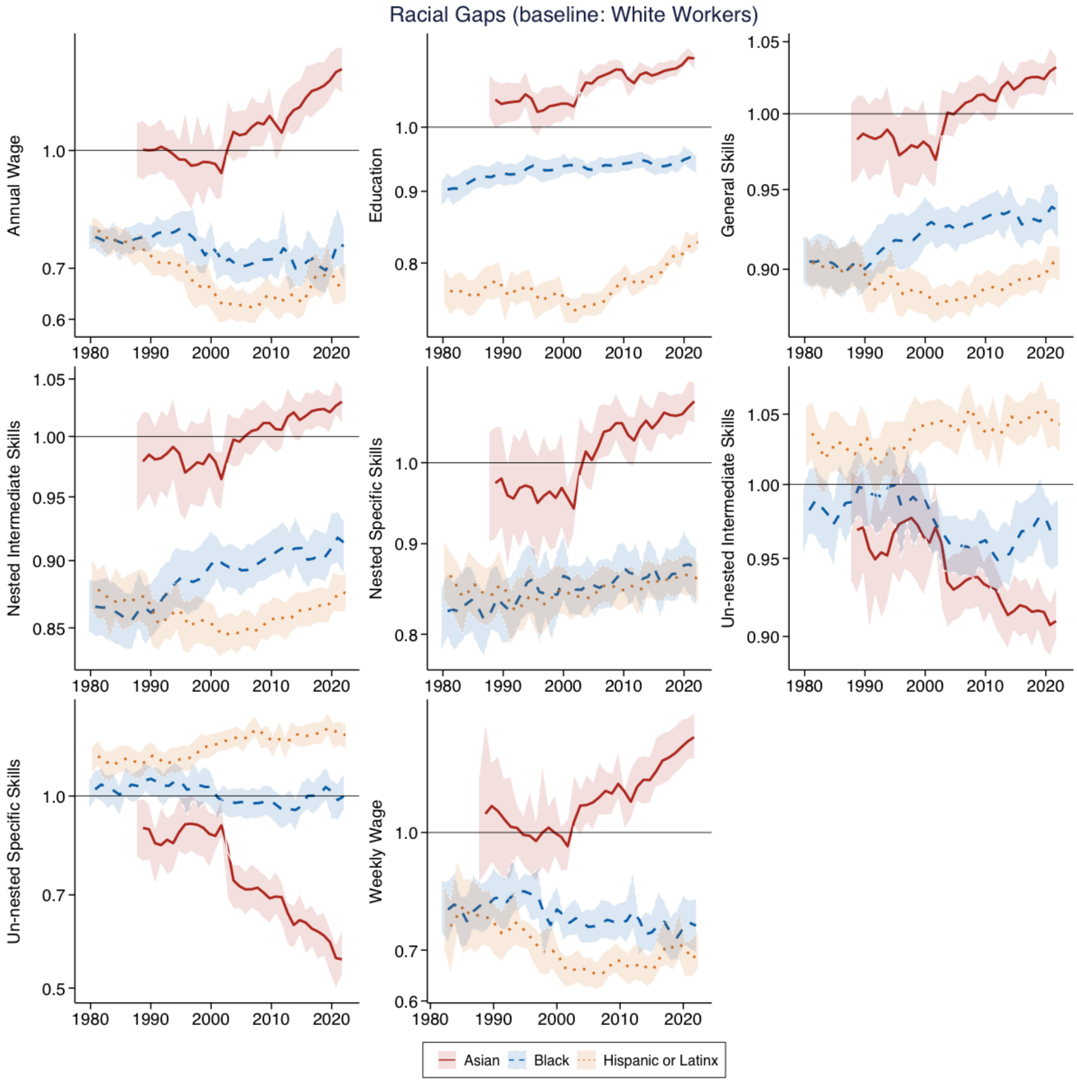

Supplementary Figure 51: Skill and Economic Race/Ethnicity Gaps over Time. Following main Fig. 6, we use White workers as the baseline and for each measure show the average for other demographics as a ratio over the average values of the White workers as the main line. In all cases, The 95% CI are obtained by a random sub-sampling. In each iteration, we take 20% of the subpopulation of interest at a certain year, for instance, Asian male and Asian female workers in 2020, and estimate all corresponding measures. Repeating this sampling and estimation process in 10,000 iterations, we obtain the distribution for each estimate (of the subpopulation of interest in that year) and derive the 95% confidential intervals.

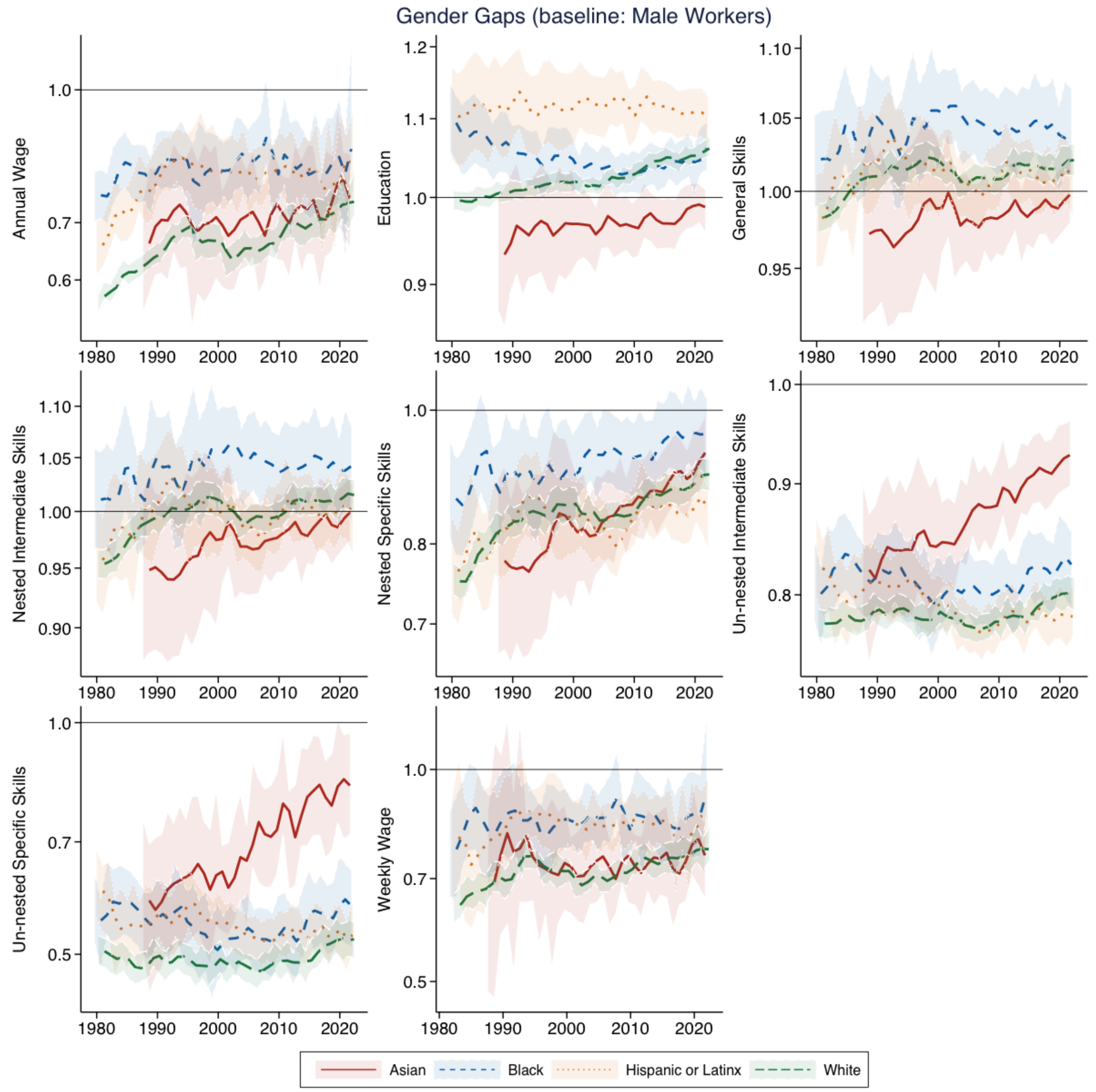

Supplementary Figure 52: Skill and Economic Inequality Across Genders over Time. Following main Fig. 6, we use male workers in each racial group as the benchmark, showing the average value for women over men for each measure and each demographic. In all cases, 95% confidence intervals are created as explained in Fig. 51.

7.4 Gender-Age Divergence of Skills across Demographic Groups

Fig. 53 replicates Fig. 4. However, it teases out time trends in skill acquisition for racial groups. The skill differentials between male and female workers that start around the age 30s (main Fig. 4) manifest across racial and ethnic groups. In most cases, female workers' (general and nested) skill accumulation plateaus in their mid-20s to 30s, while their male counterparts' skill stocks expand (even though slowly) up to their 40s and then plateaus. Section 7.1 addresses the possible role of children in the divergence of skills. Fig. 54 replicate the exercise factoring in annual economic circumstances.

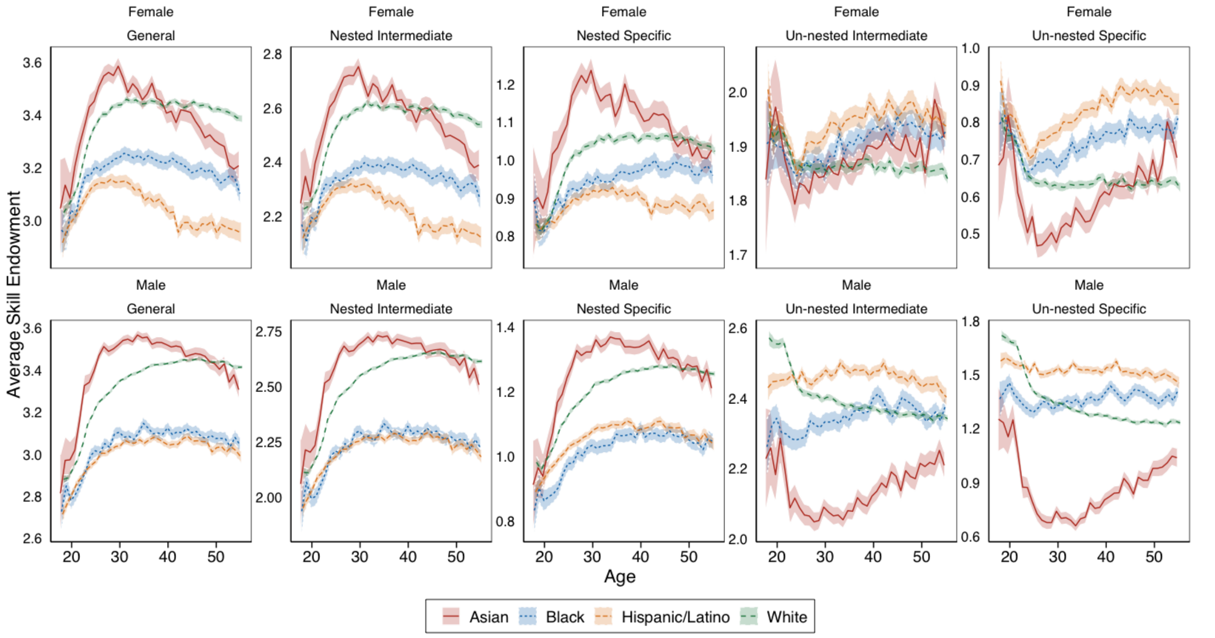

Supplementary Figure 53: Skill Acquisition Differences Across Gender and Race. Using CPS household data between 1980 and 2022, we estimate the distribution of different skills over four racial categories, namely *White, Asian, Black/African-American, Hispanic/Latino*, and for men and women, showing the average skill endowment as a line. We infer individuals' skills from the skill requirement of their detailed occupation according to O*NET 2019 and calculate a skill endowment for a given race and gender in each skill subtype. We aggregate skill endowments for racial and gender groups over age, allowing us to estimate the usage of workplace skills for these subgroups as they age. Shaded areas show 95% confidence intervals.

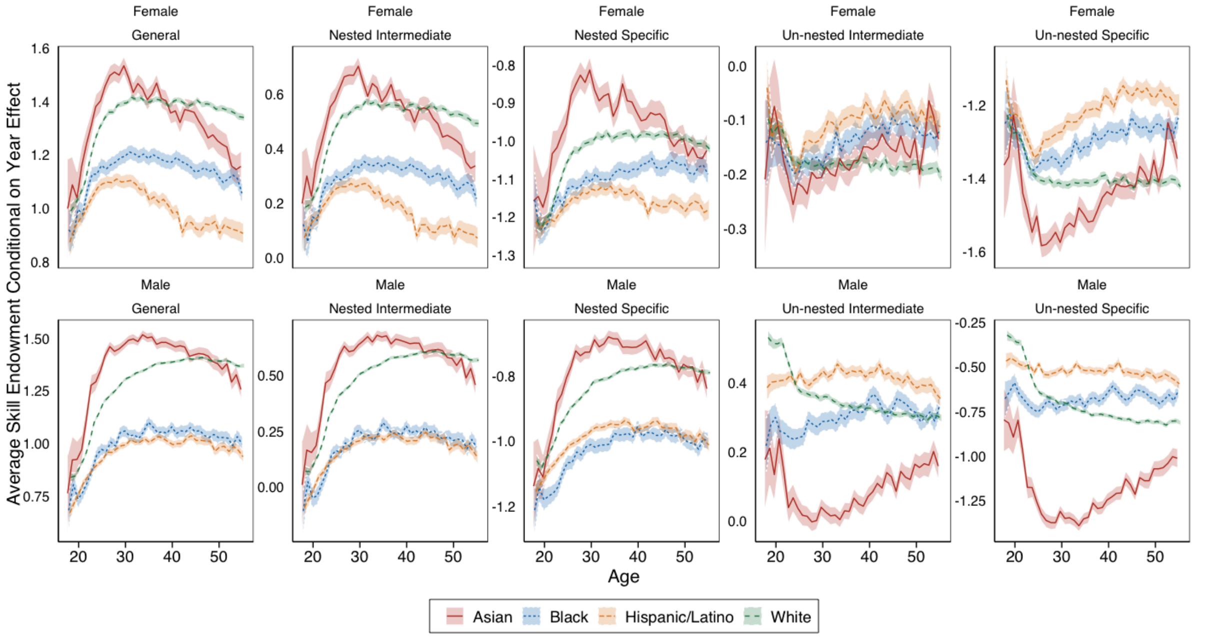

Supplementary Figure 54: Skill Acquisition Differences Across Gender and Race Conditional on Year Effects. The setup follows Fig. 53 with the minor difference that skill levels are first residualized by a year effect. The results are almost identical.

8 Historical Patterns of Skill Change for Occupations

How have occupational skills (Level and Importance) changed over time? Do they manifest our theorized co-evolution of skills at different rungs of the skill hierarchy? In other words, can we observe our inferred conditional dependence and independence on the level and importance of skills?

To answer these questions, we compared the level and importance of occupational skills reported by O*NET in 2019 and 2005. When comparing levels and importance of skills across the two years, we use a crosswalk, explained in the supplementary section 8.3, to account for the changes in the taxonomy between 2005 and 2019 [40]. We further capture changes in the skill structure by comparing the skill structure of 2019 to a past snapshot of O*NET from 2005. We produced the mentioned 2005 skill structure using the same methodology and parameters as used for the 2019 skill dependency network.

8.1 Changes in the Skill Levels

Figure 55 shows the changes in the level of each skill across occupations between 2005 and 2019. For each occupation and skill, we subtracted the level O*NET reports in 2019 from the level in 2005. For each skill, we show the density plot of occupations based on their corresponding level change. The white line for each skill denotes the median, and the dashed line corresponds to no change.

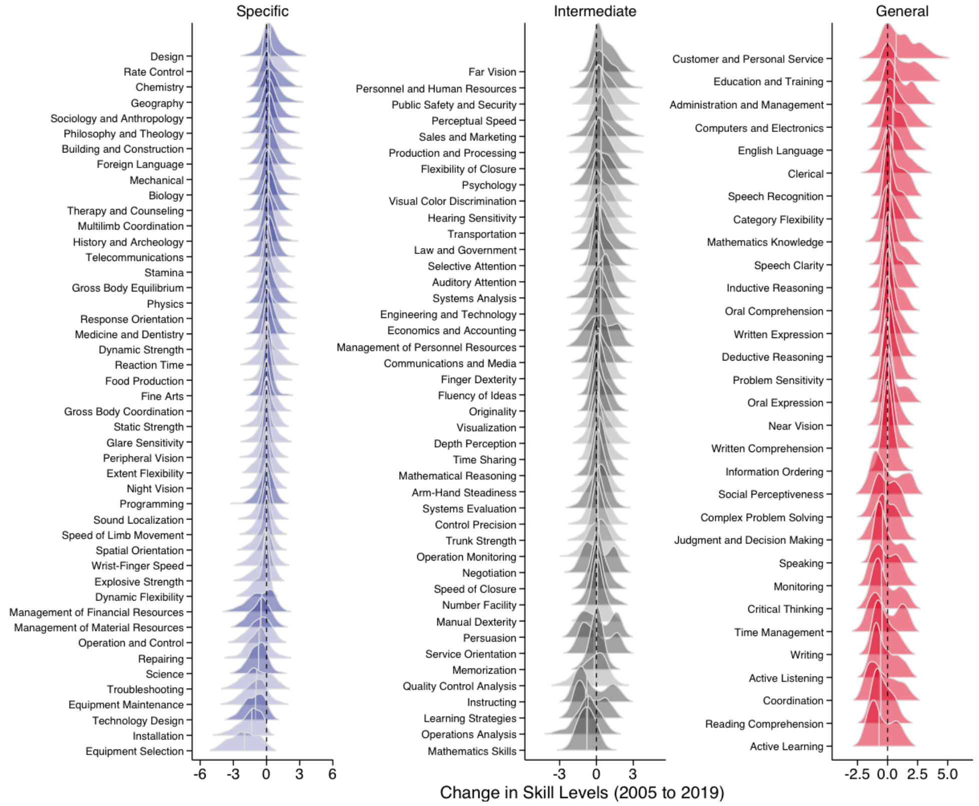

Supplementary Figure 55: Changes in the Level of Each Skill across Occupations between 2005 and 2019 as reported by O*NET. The distributions capture the changes of skill Levels across all occupations. The white line shows the median change, while the dashed black line is fixed at 0 as a possible benchmark (of no change.)

8.2 Changes in the Skill Dependency Network

Fig. 56 shows the backbone of the skill dependency network based on 2005, as appears in Fig. 6 with skill labels attached.

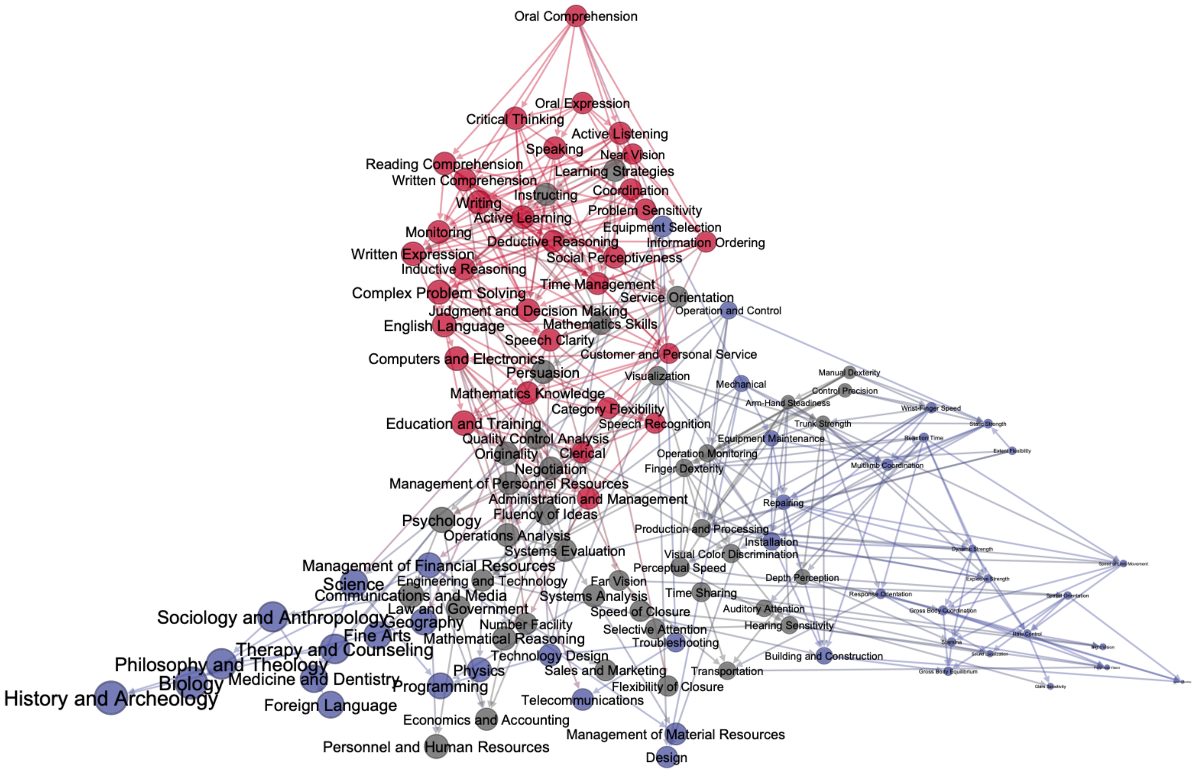

Supplementary Figure 56: Labeled Backbone of Skill Dependency for the year 2005.

In Fig. 57, we unpack the changes that manifest in distinct backbones of skill dependency networks in 2019 compared with 2005 (as shown in Fig. 6). We compare the dependency ties between all skills that are present in both 2005 and 2019 networks (Installation, Explosive strength, Sound localization, Food production, Public safety, and security in 2019 and Memorization, Food production, Chemistry, and Public safety and security in 2005 are eliminated due to a lack of statistically significant ties.) We distinguish between three types of ties: 1) *New edges*: dependency ties that were statistically insignificant in 2005 and became significant in 2019 (shown in green); 2) *Constant edges*: dependency ties that were statistically significant in both 2005 and 2019 (shown in black); 3) *Lost edges*: dependency ties that lost statistical significance in 2019 while being significant in 2005 (shown in orange). In Fig. 57, we used the layout of our main Fig. 2 (b), adjusting the distance between nodes slightly to visualize edges better. The edges are replaced with the three types described above. The pattern of changes in dependency ties offers insights into the widening gap between the nested and un-nested parts of the skill structure over time. Most new edges (green) are massed in the nested section. Particularly, a noticeable number of edges tie general skills to the most niche nested skills— these dependency ties are not visible in the backbone for better visualization. However, they are taken into account in all calculations. There are virtually no new ties formed between the general and un-nested sections. Few previously existing ties were no longer statistically significant in 2019 (orange). Therefore, the figure highlights the increasing intensity of dependencies in the nested part of the network, while a collapse of dependencies in the un-nested section. These patterns emphasize our findings about the changes in the nature of work.

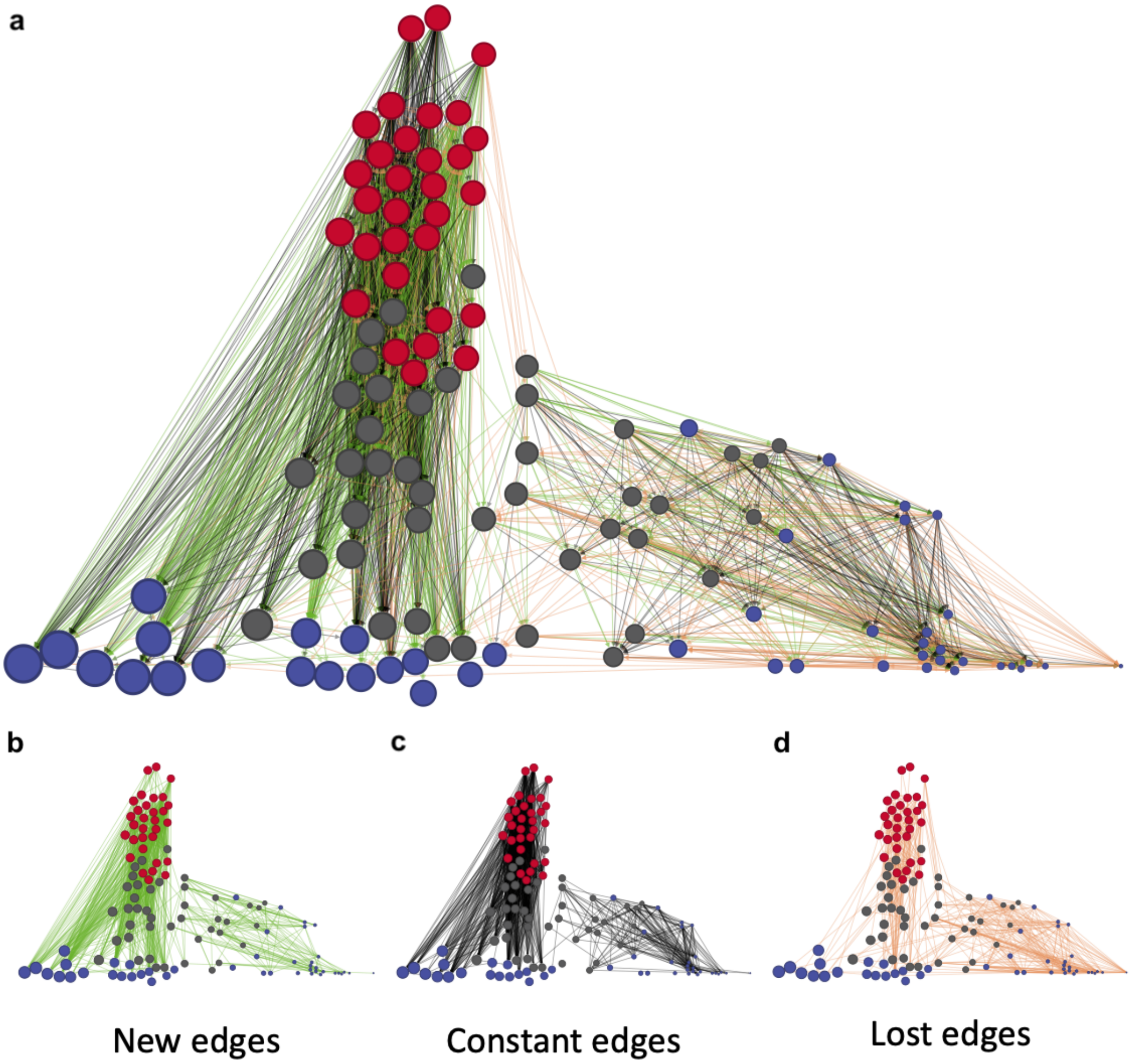

Supplementary Figure 57: Changes in the Skill Dependency Networks between 2005 and 2019. We distinguish between three types of ties: **(b)** *New edges*: dependency ties that were statistically insignificant in 2005 and became significant in 2019 (shown in green); **(c)** *Constant edges*: dependency ties that were statistically significant in both 2005 and 2019 (shown in black); **(d)** *Lost edges*: dependency ties that lost statistical significance in 2019 while being significant in 2005 (shown in orange). **(a)** The combined figure highlights the increasing intensity of dependencies in the nested part of the network, while a collapse of vertical dependencies in the un-nested section.

8.3 Occupation Taxonomy

Taxonomy has changed over time [41]. Our historical analysis comparing 2005 and 2019 data must consider such changes. 2005 O*NET complies to *O*NET SOC 2000*, while 2019 O*NET uses *O*NET SOC 2010*, with two other waves of taxonomy change between (2006 and 2009). Therefore, identically encoded occupations may not be comparable across these two years, and matching them requires a crosswalk.

While O*NET reports[5] crosswalks between each consecutive taxonomy, there is no direct crosswalk between 2005 and 2019. We created such a crosswalk to match occupations in 2005 and 2019 using the consecutive crosswalks mentioned above[6].

[5] `https://www.onetcenter.org/taxonomy.html`

[6] For instance, if occupation X_{2000} in taxonomy 2000 is linked to X_{2006} in taxonomy 2006, and X_{2006} is matched

Our crosswalk matches 968 occupations in 2019 skill data and 941 unique occupations in 2005 skill data. Out of 1,334 records in our crosswalk, 362 correspond to occupations whose SOC codes have changed. Fig. 58 shows the number and percentage of occupations in the skill data we could match across both years. Groups such as Computer and Math, Engineering, Health, Management, and Business have the most number of occupations with skill information added between 2005 and 2019. Therefore, it is important to consider the unbalanced nature of the data, when interpreting analysis on the changes of skills using O*NET.

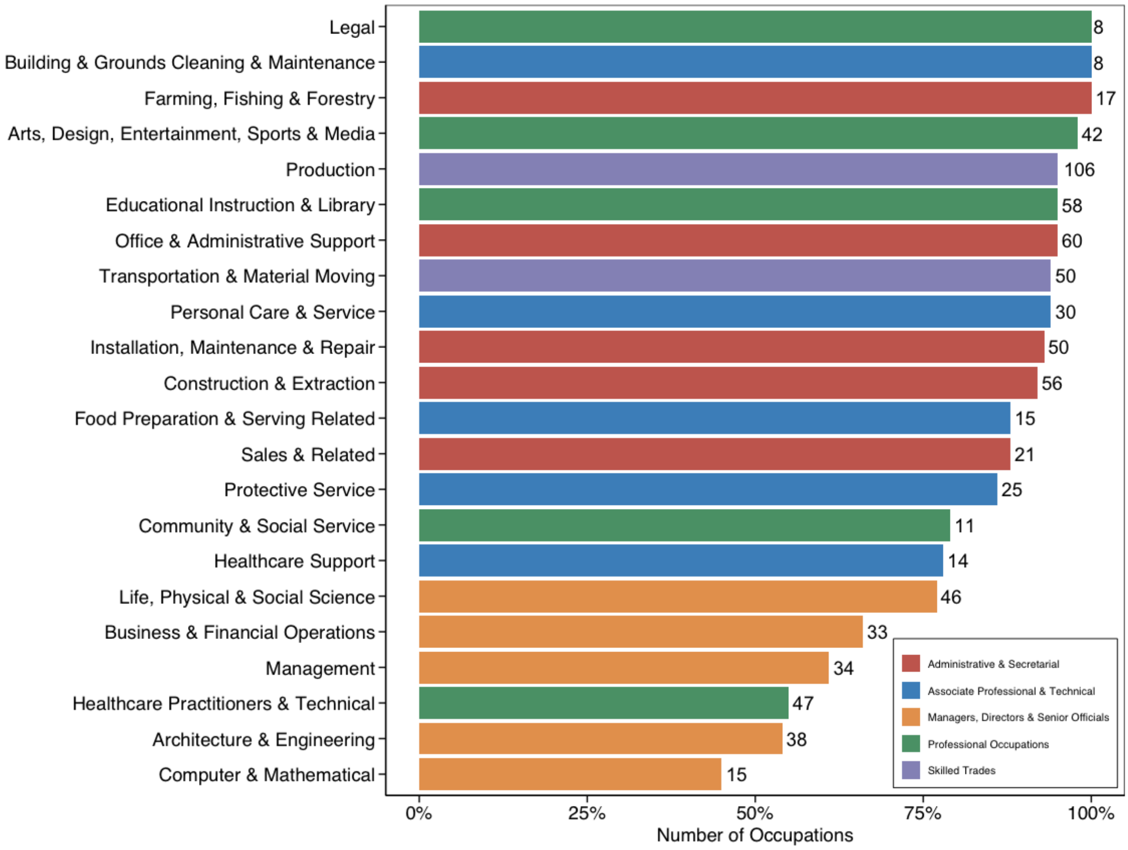

Supplementary Figure 58: Percentage and Number of Occupations from Occupational Groups Matched between 2005 and 2010.

to X_{2009} in taxonomy 2009, and X_{2009} is matched to X_{2010} in taxonomy 2010, our crosswalk will link X_{2000} to X_{2010}.

9 Robustness Checks with Management and Admin Occupations and Social Skills

In this section, we offer supplementary analyses to our main findings and produce pieces of evidence refuting several alternative explanations. We begin by examining whether managerial occupations drive the importance of general skills. Next, we discuss whether the general skills' effect is driven primarily by social skills, whose importance has been a topic of growing emphasis. We continue by offering more detailed analyses of historical changes in skill requirements and skill acquisition with age.

9.1 Role of Management and Administrative Occupations

Here, we test if the importance of general skills in the wage premium (Fig. 5) is driven by management or administrative occupations. To do so, we identify such occupations, exclude them from our analyses at various stages, and examine the resulting changes. Table 10 lists these occupations with their annual wage and educational requirements. We identify those using the Standard Occupational Code (SOC) at the 2-digit level, wherein "11" denotes managerial occupations. In addition, we use descriptive terms for these occupation titles (manager, administrator, and director) to identify relevant occupations further using their titles. In total, we found and omitted a total of 75 occupations out of 968 occupations and collected them, sorted based on average annual wage and required education, in Table 10.

Supplementary Table 10: List of Manager Occupations and their Annual Wage and Education Requirements in our Sample.

	Code	Title	Wage	Education
1	11-1011.00	Chief Executives	$ 170.5K	7.540
2	11-1011.03	Chief Sustainability Officers	$ 170.5K	6.920
3	11-9041.00	Architectural and Engineering Managers	$ 135.8K	6.720
4	11-9041.01	Biofuels/Biodiesel Technology and Product Devel...	$ 135.8K	6.480
5	11-2021.00	Marketing Managers	$ 124.2K	6.680
6	11-3111.00	Compensation and Benefits Managers	$ 124.1K	6.330
7	11-3021.00	Computer and Information Systems Managers	$ 123.8K	5.550
8	11-2022.00	Sales Managers	$ 122.3K	6.040
9	11-9121.00	Natural Sciences Managers	$ 122.1K	8.130
10	11-9121.02	Water Resource Specialists	$ 122.1K	6.860
11	11-9121.01	Clinical Research Coordinators	$ 122.1K	6.060
12	11-3031.01	Treasurers and Controllers	$ 117.7K	7.070
13	11-3031.02	Financial Managers, Branch or Department	$ 117.7K	5.440
14	11-2011.00	Advertising and Promotions Managers	$ 116.3K	5.210
15	11-3061.00	Purchasing Managers	$ 114.3K	6.150
16	11-3131.00	Training and Development Managers	$ 112K	6.630
17	11-3051.01	Quality Control Systems Managers	$ 109.6K	6.030
18	11-3051.04	Biomass Power Plant Managers	$ 109.6K	5.290
19	11-3051.03	Biofuels Production Managers	$ 109.6K	4.980
20	11-3051.00	Industrial Production Managers	$ 109.6K	4.920
21	11-3051.06	Hydroelectric Production Managers	$ 109.6K	4.170
22	11-3051.02	Geothermal Production Managers	$ 109.6K	4.060
23	11-3121.00	Human Resources Managers	$ 109.4K	6.300
24	11-9033.00	Education Administrators, Postsecondary	$ 106.1K	9.250
25	11-9111.00	Medical and Health Services Managers	$ 104.8K	6.080
26	11-1021.00	General and Operations Managers	$ 103.8K	4.920
27	11-9021.00	Construction Managers	$ 95.7K	5.680
28	11-3071.03	Logistics Managers	$ 94.1K	6.170

Supplementary Table 10: List of Manager Occupations and their Annual Wage and Education Requirements in our Sample.

	Code	Title	Wage	Education
29	11-3071.02	Storage and Distribution Managers	$ 94.1K	4.620
30	11-3071.01	Transportation Managers	$ 94.1K	4.370
31	11-9032.00	Education Administrators, Elementary and Second...	$ 91.6K	7.820
32	27-1011.00	Art Directors	$ 88.7K	6.250
33	11-9039.01	Distance Learning Coordinators	$ 84.6K	7.550
34	11-9039.02	Fitness and Wellness Coordinators	$ 84.6K	6.580
35	15-2041.02	Clinical Data Managers	$ 83.7K	6.100
36	11-9071.00	Gaming Managers	$ 81.6K	3.680
37	11-9161.00	Emergency Management Directors	$ 79.8K	6.120
38	11-9131.00	Postmasters and Mail Superintendents	$ 77K	3.030
39	13-1011.00	Agents and Business Managers of Artists, Perfor...	$ 76.7K	5.230
40	11-9013.01	Nursery and Greenhouse Managers	$ 76.6K	4.960
41	11-9013.03	Aquacultural Managers	$ 76.6K	4.420
42	11-9013.02	Farm and Ranch Managers	$ 76.6K	3.880
43	19-1031.02	Range Managers	$ 67.3K	5.950
44	11-9151.00	Social and Community Service Managers	$ 66.8K	6.310
45	47-1011.03	Solar Energy Installation Managers	$ 66.1K	3.680
46	25-9031.00	Instructional Coordinators	$ 64.6K	7.700
47	27-2012.04	Talent Directors	$ 62.9K	6.140
48	27-2012.05	Technical Directors/Managers	$ 62.9K	5.770
49	27-2012.03	Program Directors	$ 62.9K	5.080
50	27-2012.02	Directors- Stage, Motion Pictures, Television, ...	$ 62.9K	4.570
51	11-9081.00	Lodging Managers	$ 60.9K	4.890
52	11-9141.00	Property, Real Estate, and Community Associatio...	$ 60.2K	5.040
53	11-9051.00	Food Service Managers	$ 56.7K	2.540
54	39-4031.00	Morticians, Undertakers, and Funeral Directors	$ 56.5K	4.810
55	27-2041.01	Music Directors	$ 56.2K	8.210
56	11-9031.00	Education Administrators, Preschool and Childca...	$ 49.2K	4.140
57	21-2021.00	Directors, Religious Activities and Education	$ 48.9K	6
58	11-9199.03	Investment Fund Managers	-	7.410
59	11-9199.01	Regulatory Affairs Managers	-	6.500
60	15-1141.00	Database Administrators	-	6.440
61	11-9199.04	Supply Chain Managers	-	6.430
62	11-9199.07	Security Managers	-	6.150
63	11-9199.11	Brownfield Redevelopment Specialists and Site M...	-	6.120
64	11-2031.00	Public Relations and Fundraising Managers	-	6.100
65	11-9199.10	Wind Energy Project Managers	-	6.090
66	11-9199.09	Wind Energy Operations Managers	-	5.860
67	15-1199.09	Information Technology Project Managers	-	5.860
68	11-9061.00	Funeral Service Managers	-	5.710
69	11-9199.02	Compliance Managers	-	5.650
70	15-1142.00	Network and Computer Systems Administrators	-	5.580
71	15-1199.03	Web Administrators	-	5.350
72	11-9199.08	Loss Prevention Managers	-	4.950
73	39-1021.01	Spa Managers	-	4.220
74	11-3011.00	Administrative Services Managers	-	3.960
75	53-1021.01	Recycling Coordinators	-	3.890

In conclusion, our findings of skill clusters, skills wage premiums, and educational requirements are robust to the presence/absence of managerial occupations. In Figs 59 through 61 we use k-mean clustering to group skills into profiles without considering managerial occupations. The results complement the supplementary section 1, establishing the robustness of our skill profiles. Fig. 63 shows that excluding managerial occupations does not diminish the acquisition

of general and nested skills over time by analyzing occupational median age, skill composition of synthetic birth cohorts based on CPS microdata, and analysis of our resume sample. Fig. 62 examines wage premiums and educational requirements of occupations in general and specific skills while excluding the information on managerial occupations in the analysis. We find these occupations do not drive the wage premiums, and educational requirements persist. These findings supplement the results in Fig. 5.

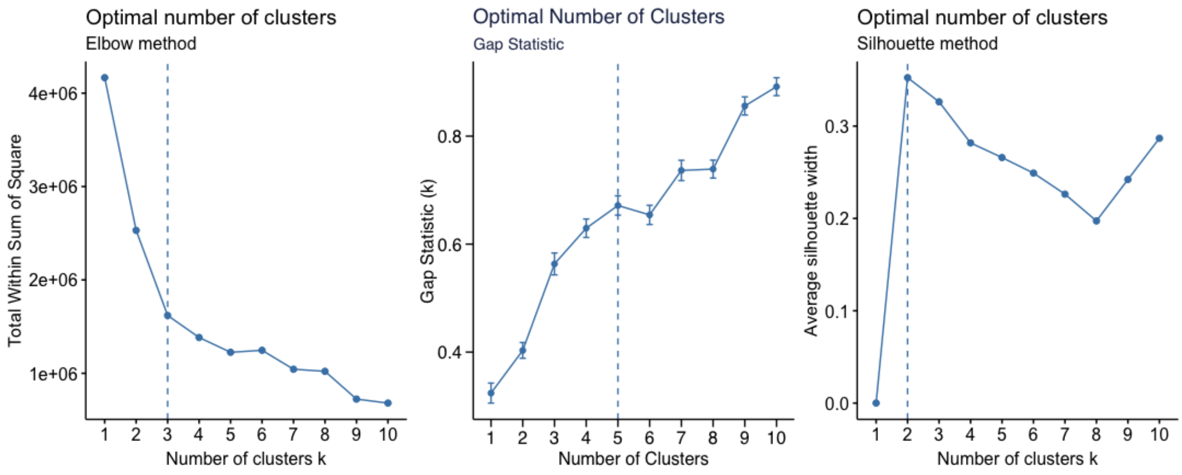

Supplementary Figure 59: Determining k for k-mean Clustering at the Absence of Managerial Occupations. Reproduction of Fig. 1 without management and administrative occupations. We use the Elbow method, Gap statistic ($n_{MonteCarloiterations} = 250$, standard error as error bars), and Silhouette analysis to test the optimal k.

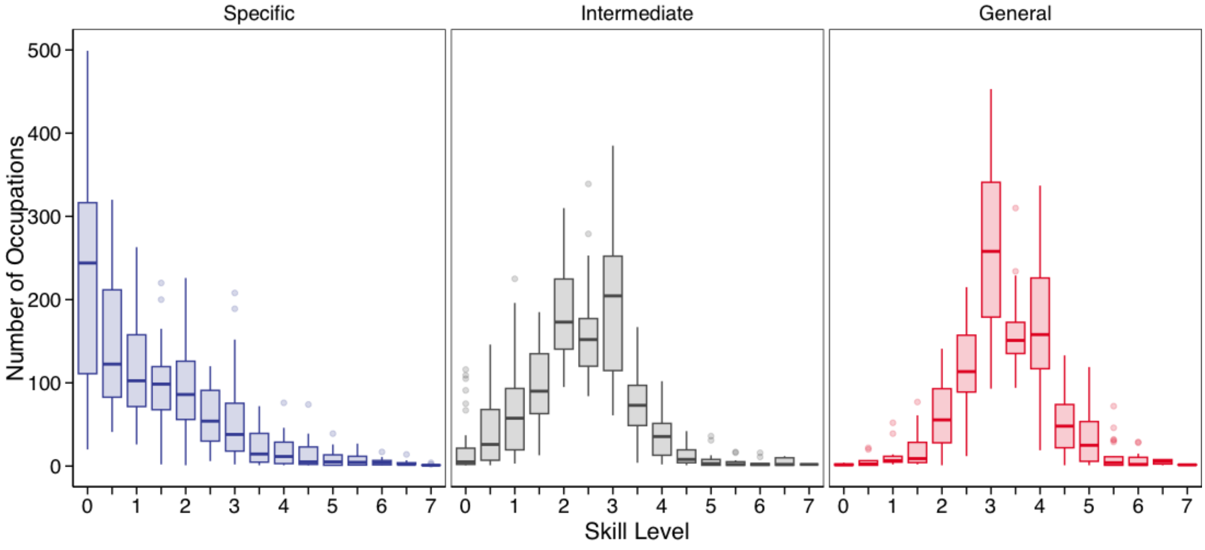

Supplementary Figure 60: Grouping Skills at the Absence of Managerial Occupations. Reproduction of Fig. 1 without management and administration occupations. The number of occupations requiring skills of a group at different levels are shown in box plots (minima and maxima are the smallest and largest datapoint within the interquartile range, upper and lower bounds of each box show the first and third quartles, and the central line is the median.) ($n_{General} = 34$, $n_{Intermediate} = 40$, $n_{Specific} = 46$).

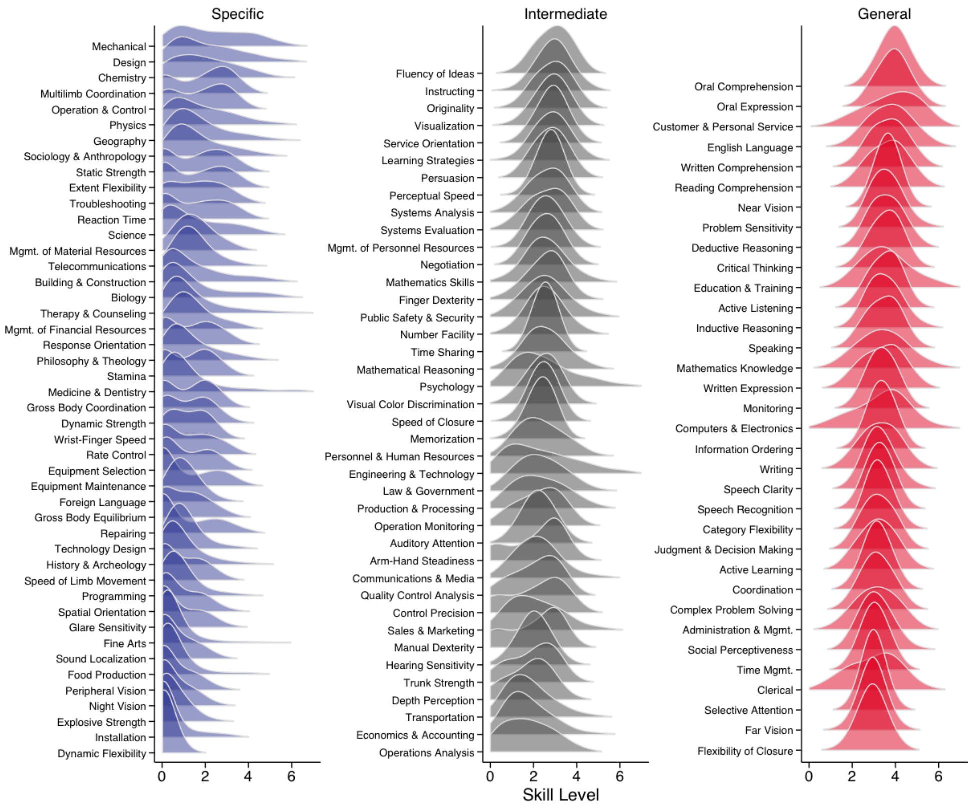

Supplementary Figure 61: Detailed Assignment of Skills among Clusters. Reproduction of Fig. 2 without management and administration occupations ($n_{occ} = 893$). Error bars in a and b report 95% CI, and regression standard errors in c and d.

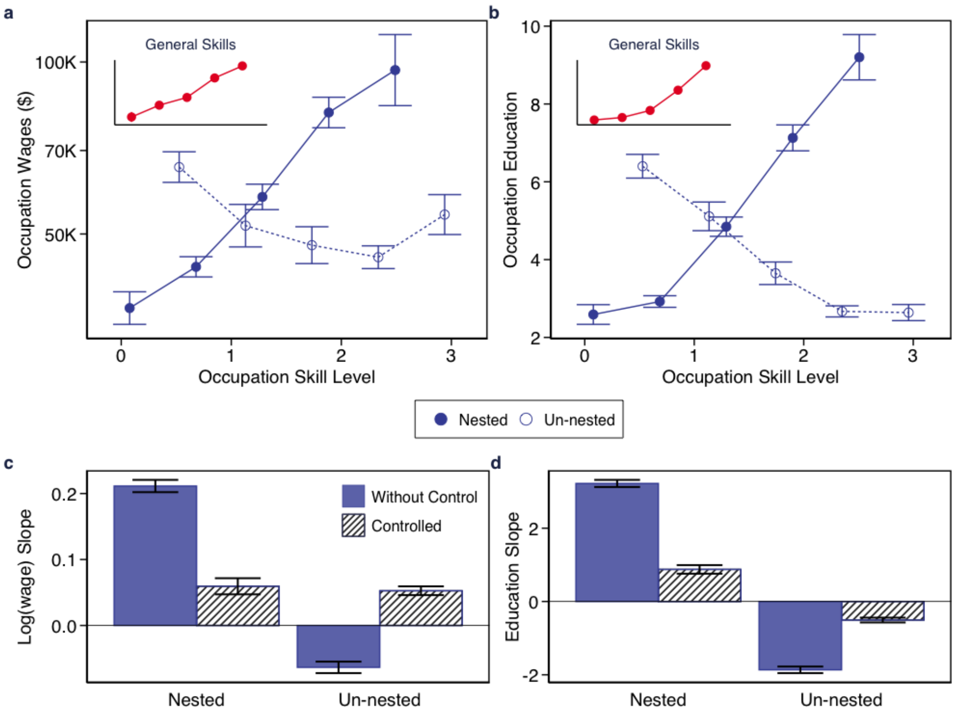

Supplementary Figure 62: Reproduction of Fig. 5 in the main text without management and administration occupations. Managerial occupations do not drive the wage premiums ($n = 726$) and the educational requirement ($n = 893$).

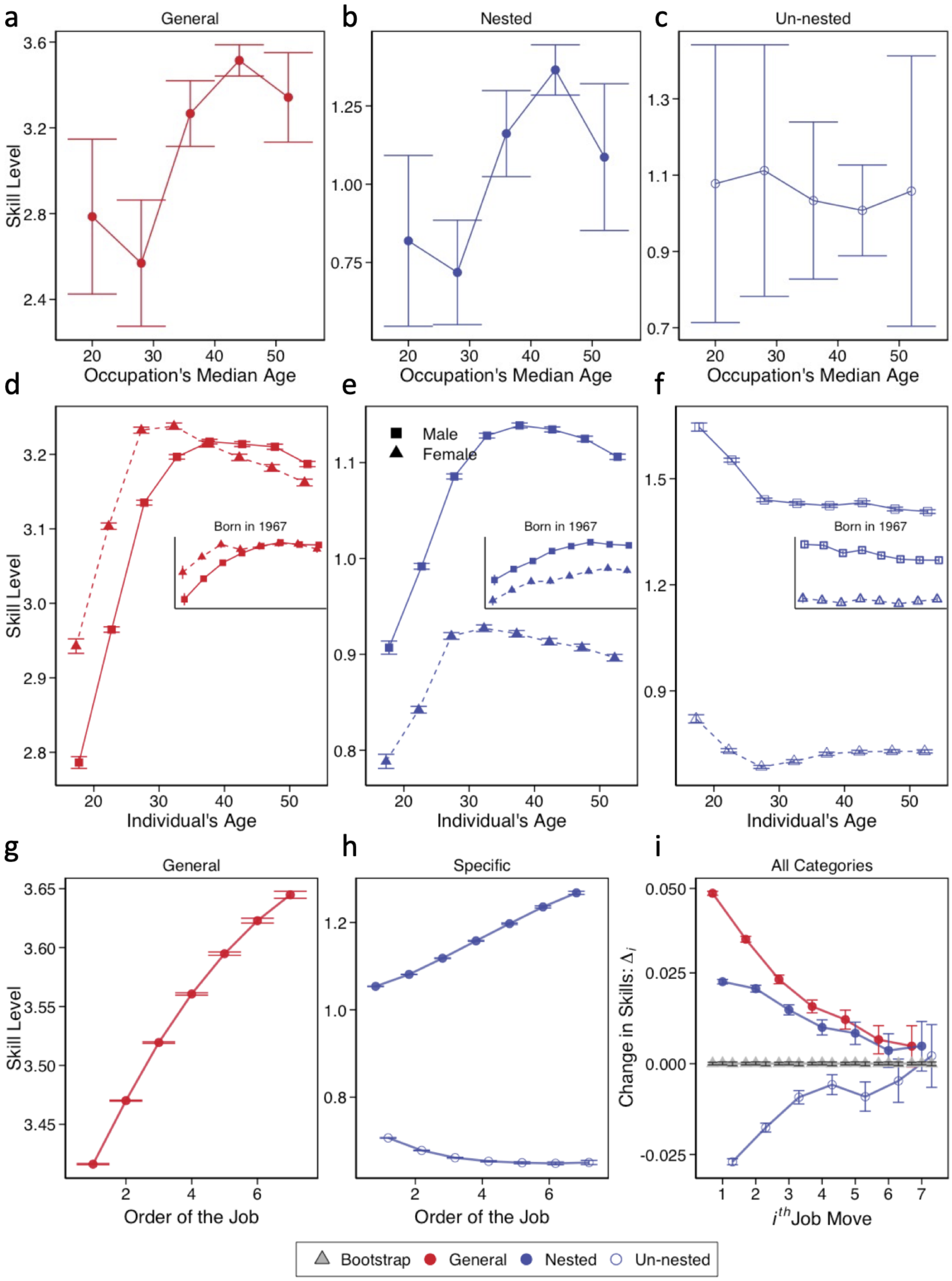

Supplementary Figure 63: Reproduction of Fig. 3 without Management and Administration occupations. The findings are consistent, suggesting managerial occupations are not the primary drivers of increases in general or nested skills over time. **(a-c)** replicate the analysis of main Fig. 4 (a-c) on occupations' median age in the absence fo managerial occupations. **(d-f)** follows analysis Fig. 4 (d-f) of synthetic birth cohorts identified in CPS microdata except for excluding observations on individuals who held managerial occupations. **(g-i** follows the analysis of Fig. 4 (g-i) on resume data except for excluding observations on individuals who held managerial occupations.

9.2 Role of Social Skills

Given the growing importance of social skills [42], we examine the extent to which they shape the role we observed for general skills in our work. There are six *social skills* in O*NET taxonomy. They are *Social perceptiveness*, the skill of being aware of other's reactions and understanding why they react as they do; *Coordination*, the skill to adjust actions in relation to others' actions; *Persuasion*, the skill to persuade others to change their minds or behavior; *Negotiation*, bringing others together and trying to reconcile differences; *Instructing*, the skill to teach others how to do something; and *Service orientation*, actively looking for ways to help people.

In Fig. 64-(a), we annotated these social skills in our skill hierarchy of the main text as well as the hierarchy of 2005 data (insets). We find these skills fall within our categories of general (Coordination and Social perceptiveness) and nested skill categories (Instructing, Service orientation, Persuasion, and Negotiation); and these skills are more demanded in 2019 than in 2005 as shown in Fig. 64-(b).

The average levels at which occupations in 2019 use negotiation, persuasion, social perceptiveness, and service orientation skills surpassed their levels in 2005. However, social skills' position in the skill dependency network has moderately moved away from the most broadly used skills. This means their comparative role has specialized moderately despite their absolute demand rise. Fig. 64 (c) shows the changes in the level of each skill category resulting from omitting social skills. As these skills belong to general and nested intermediate categories, scores across other categories do not change. However, changes in the affected subgroups are also minimal, leading to a 0.998 correlation before and after social skills omission. This offers evidence that social skills do not influence the significance of general skills. We repeated analyses of wage premiums (Fig. 5 in the main text) on categories excluding the aforementioned six social skills, ad our findings are intact as shown in Fig. 64 (d-e).

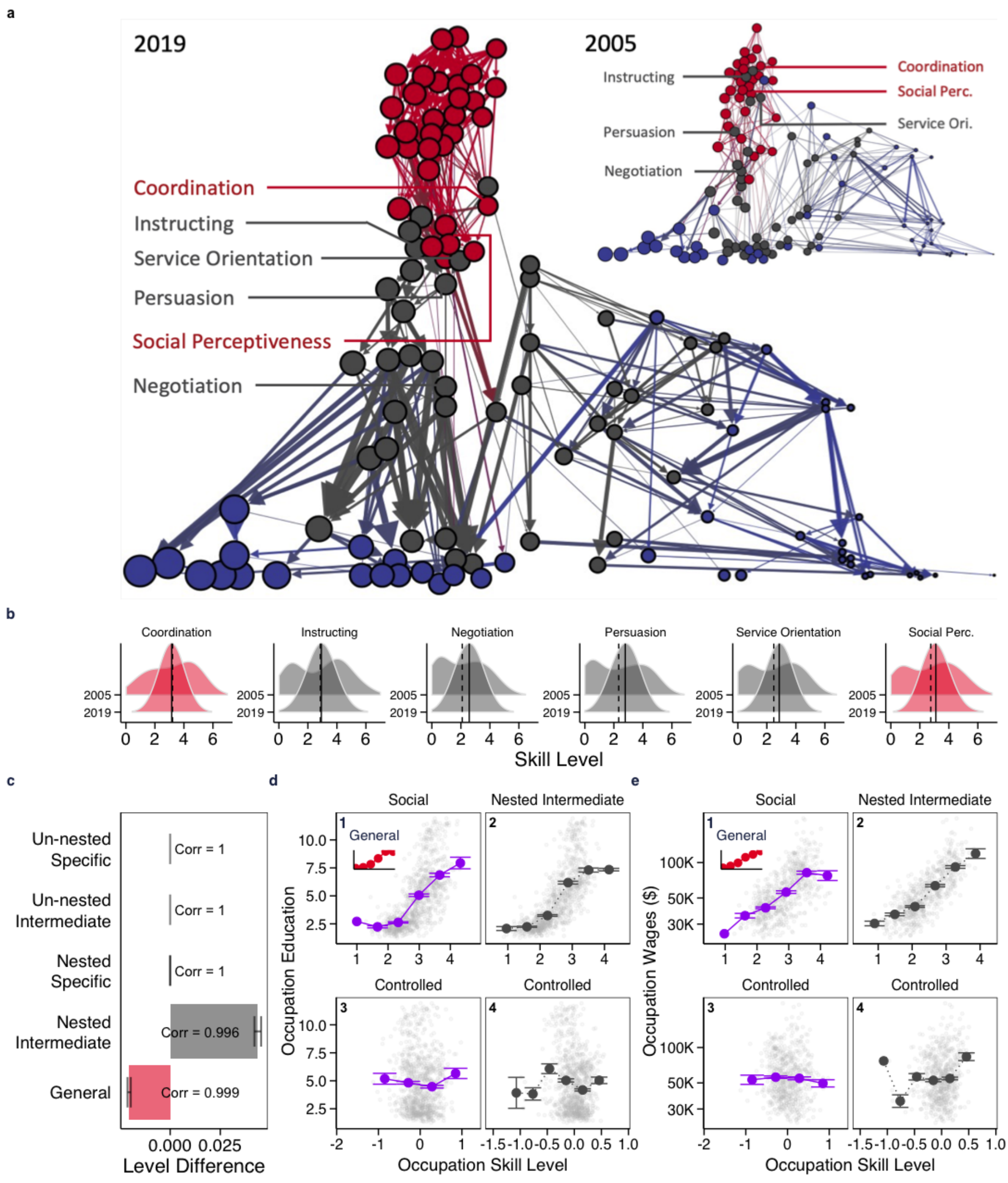

Supplementary Figure 64: Analysis of the Social Skills and their Relationship with our Work. **(a)** Annotates the skill hierarchy of main text Fig. 2 and that of the year 2005 with social skills included in O*NET. **(b)** Skill profiles of social skills in 2005 (average shown as dashed line) and in 2019 (average shown as a solid line). **(c)** The differences in skill levels when social skills are excluded ($n_{moves} = 12,561,319$). **(d-e)** The equivalent plots of Fig. 5 for educaton ($n_{occ} = 968$) and wage ($n_{occ} = 774$) without social skills. Error bars in c-d report 95% CI.

References

1. Frey, C. B. & Osborne, M. A. The future of employment: How susceptible are jobs to computerisation? *Technological Forecasting and Social Change* **114,** 254–280. arXiv: 1602.03506 (2017).
2. Rousseeuw, P. J. Silhouettes: A graphical aid to the interpretation and validation of cluster analysis. *Journal of Computational and Applied Mathematics* **20,** 53–65 (1987).
3. Tibshirani, R., Walther, G. & Hastie, T. Estimating the number of clusters in a data set via the gap statistic. *Journal of the Royal Statistical Society: Series B (Statistical Methodology)* **63,** 411–423 (2001).
4. Hidalgo, C. A. & Hausmann, R. The building blocks of economic complexity. eng. *Proceedings of the National Academy of Sciences of the United States of America* **106,** 10570–10575 (2009).
5. Bustos, S. & Yıldırım, M. A. Production Ability and economic growth. *Research Policy* **51,** 104153 (2022).
6. Bascompte, J., Jordano, P., Melián, C. J. & Olesen, J. M. The nested assembly of plant–animal mutualistic networks. *Proceedings of the National Academy of Sciences* **100,** 9383–9387 (2003).
7. Fortuna, M. A. *et al.* Nestedness versus modularity in ecological networks: two sides of the same coin? *Journal of Animal Ecology* **79,** 811–817 (2010).
8. Saavedra, S., Stouffer, D. B., Uzzi, B. & Bascompte, J. Strong contributors to network persistence are the most vulnerable to extinction. *Nature* **478,** 233–235 (2011).
9. Bastolla, U. *et al.* The architecture of mutualistic networks minimizes competition and increases biodiversity. *Nature* **458,** 1018–1020 (2009).
10. Almeida-neto, M., Guimarães, P., Jr, P. R. G., Loyola, R. D. & Ulrich, W. A consistent metric for nestedness analysis in ecological systems: reconciling concept and measurement. *Oikos* **117,** 1227–1239 (2008).
11. Serrano, M. Á., Boguñá, M. & Vespignani, A. Extracting the multiscale backbone of complex weighted networks. *Proceedings of the National Academy of Sciences* **106,** 6483–6488 (2009).
12. Stone, L. & Roberts, A. The checkerboard score and species distributions. *Oecologia* **85,** 74–79 (1990).
13. Wright, D. & Reeves, J. On the meaning and measurement of nestedness of species assemblages. *Oecologia* **92,** 416–428 (1992).
14. Atmar, W. & Patterson, B. D. The measure of order and disorder in the distribution of species in fragmented habitat. *Oecologia* **96,** 373–382 (1993).
15. Jo, W. S., Park, J., Luhur, A., Kim, B. J. & Ahn, Y.-Y. *Extracting hierarchical backbones from bipartite networks* 2020.
16. Mones, E., Vicsek, L. & Vicsek, T. Hierarchy measure for complex networks. eng. *PloS one* **7,** e33799 (2012).
17. Kosack, S. *et al.* Functional structures of US state governments. *Proceedings of the National Academy of Sciences* **115,** 11748–11753 (2018).
18. Neffke, F. M. H. The value of complementary co-workers. *Science Advances* **5** (2019).

19. Anderson, K. A. Skill networks and measures of complex human capital. *Proceedings of the National Academy of Sciences* **114,** 12720–12724 (2017).

20. Alabdulkareem, A. *et al.* Unpacking the polarization of workplace skills. *Science Advances* **4,** 1–10 (2018).

21. Frank, M. R. *et al.* Toward understanding the impact of artificial intelligence on labor. *Proceedings of the National Academy of Sciences,* 201900949 (2019).

22. Jovanovic, B. & Nyarko, Y. Stepping-stone mobility. *Carnegie-Rochester Conference Series on Public Policy* **46,** 289–325 (1997).

23. Nedelkoska, L., Patt, A. & Ederer, P. Learning by problem solving. *Available at SSRN* (2015).

24. Hong, I., Frank, M. R., Rahwan, I., Jung, W. S. & Youn, H. The universal pathway to innovative urban economies. *Science Advances* **6,** 1–7 (2020).

25. Balland, P.-A. *et al.* Complex economic activities concentrate in large cities. *Nature Human Behaviour* (2020).

26. Glaeser, E. L. Learning in Cities. *Journal of Urban Economics* **46,** 254–277 (1999).

27. Wheeler, C. H. Search, Sorting, and Urban Agglomeration. *Journal of Labor Economics* **19,** 879–899 (2001).

28. Youn, H. *et al.* Scaling and universality in urban economic diversification. *Journal of The Royal Society Interface* **13,** 20150937. eprint: `https://royalsocietypublishing.org/doi/pdf/10.1098/rsif.2015.0937` (2016).

29. Gomez-Lievano, A., Patterson-Lomba, O. & Hausmann, R. Explaining the prevalence, scaling and variance of urban phenomena. *Nature Human Behaviour* **1,** 0012 (2016).

30. Bettencourt, L. M. A., Samaniego, H. & Youn, H. Professional diversity and the productivity of cities. *Scientific Reports* **4,** 5393 (2014).

31. Gomez-Lievano, A. & Patterson-Lomba, O. Estimating the drivers of urban economic complexity and their connection to economic performance. *Royal Society Open Science* **8,** 210670. arXiv: `1812.02842` (2021).

32. Lucas, R. E. On the mechanics of economic development. *Journal of Monetary Economics* **22,** 3–42 (1988).

33. Bacolod, M., Blum, B. S. & Strange, W. C. Skills in the city. *Journal of Urban Economics* **65,** 136–153 (2009).

34. Glaeser, E. L. & Mare, D. C. Cities and Skills. *Journal of Labor Economics* **19,** 316–342 (2001).

35. Tong, D., Wu, L. & Evans, J. A. *Low-skilled Occupations Face the Highest Re-skilling Pressure* 2021.

36. Bertrand, M., Goldin, C. & Katz, L. F. Dynamics of the gender gap for young professionals in the corporate and financial sectors (2009).

37. Goldin, C. Hours flexibility and the gender gap in pay. *Center for American Progress* **31** (2015).

38. Canon, M. & Golan, L. Gender Pay Gap May Be Linked to Flexible and Irregular Hours. *The Regional Economist* (2016).

39. Cha, Y. & Weeden, K. A. Overwork and the slow convergence in the gender gap in wages. *American Sociological Review* **79,** 457–484 (2014).

40. Hopson, A. Mapping Employment Projections and O*NET data: a methodological overview. *Monthly Labor Review,* 1–11 (2021).

41. Park, J., Sun, L. & Youn, H. *Industrial Topics in Urban Labor System* 2020.

42. Liu, Y. & Grusky, D. B. The Payoff to Skill in the Third Industrial Revolution. *The American journal of sociology* **118,** 1330–1374 (2013).
