# Supplementary material for: Skill dependencies uncover nested human capital
Source: Nat Hum Behav. 2025 Feb 24;9(4):673–87. doi: 10.1038/s41562-024-02093-2 (PMC12018457; doi:10.1038/s41562-024-02093-2)
Supplement: Supplementary file 2 — Reporting Summary [file 41562_2024_2093_MOESM2_ESM.pdf]

nature portfolio

Corresponding author(s): Hyejin Youn

Last updated by author(s): 11/04/2024

Reporting Summary

Nature Portfolio wishes to improve the reproducibility of the work that we publish. This form provides structure for consistency and transparency in reporting. For further information on Nature Portfolio policies, see our Editorial Policies and the Editorial Policy Checklist.

Statistics

For all statistical analyses, confirm that the following items are present in the figure legend, table legend, main text, or Methods section.

n/a	Confirmed	
☐	☒	The exact sample size (n) for each experimental group/condition, given as a discrete number and unit of measurement
☒	☐	A statement on whether measurements were taken from distinct samples or whether the same sample was measured repeatedly
☒	☐	The statistical test(s) used AND whether they are one- or two-sided<br>*Only common tests should be described solely by name; describe more complex techniques in the Methods section.*
☐	☒	A description of all covariates tested
☒	☐	A description of any assumptions or corrections, such as tests of normality and adjustment for multiple comparisons
☐	☒	A full description of the statistical parameters including central tendency (e.g. means) or other basic estimates (e.g. regression coefficient) AND variation (e.g. standard deviation) or associated estimates of uncertainty (e.g. confidence intervals)
☒	☐	For null hypothesis testing, the test statistic (e.g. F, t, r) with confidence intervals, effect sizes, degrees of freedom and P value noted<br>*Give P values as exact values whenever suitable.*
☒	☐	For Bayesian analysis, information on the choice of priors and Markov chain Monte Carlo settings
☒	☐	For hierarchical and complex designs, identification of the appropriate level for tests and full reporting of outcomes
☒	☐	Estimates of effect sizes (e.g. Cohen's d, Pearson's r), indicating how they were calculated

Our web collection on statistics for biologists contains articles on many of the points above.

Software and code

Policy information about availability of computer code

Data collection	This study has used three publicly available data sources, Occupational Information Network (O*NET), Current Population Survey (CPS), occupational information, including wages, employment and age demographic prepared by the US Bureau of Labor Statistics (BLS). We have also utilized a proprietary source containing a database of anonymized resume, widely used in the literature.
Data analysis	All algorithms previously developed and used in processing and preparing data and analysis have been cited. The code used for data processing and analysis is available at https://github.com/mohhoss/Nested-Skills-in-Labor-Ecosystems.

For manuscripts utilizing custom algorithms or software that are central to the research but not yet described in published literature, software must be made available to editors and reviewers. We strongly encourage code deposition in a community repository (e.g. GitHub). See the Nature Portfolio guidelines for submitting code & software for further information.

Data

Policy information about availability of data

All manuscripts must include a data availability statement. This statement should provide the following information, where applicable:

- Accession codes, unique identifiers, or web links for publicly available datasets
- A description of any restrictions on data availability
- For clinical datasets or third party data, please ensure that the statement adheres to our policy

This study has used three publicly available data sources: Occupational Information Network (O*NET): https://www.onetonline.org, Current Population Survey

(CPS): https://www.bls.gov/cps, occupational information, including wages, employment and age demographic prepared by the US Bureau of Labor Statistics (BLS): https: //www.bls.gov/oes. Anonymized resumes in Fig. 4 (g-i) are proprietary data.

Research involving human participants, their data, or biological material

Policy information about studies with human participants or human data. See also policy information about sex, gender (identity/presentation), and sexual orientation and race, ethnicity and racism.

Reporting on sex and gender	Studying genders is a not a central theme of this study. However, we have utilized the richness of Current Population Survey (publicly available) to uncover possible sources of social and economic gender disparities as implied by our framework. In consistent with the Nature portfolio policy, we have adopted the term "gender", given the social and cultural context of our study. Where applicable, we have articulated how our findings differ as it pertains to different genders. To be clear, Current Population Survey is the only data source used where (merely binary) gender information is made available.
Reporting on race, ethnicity, or other socially relevant groupings	Studying race and ethnicity is a not a central theme of this study. However, Current Population Survey (publicly available) includes self-declared information on race/ethnicity of surveyed individuals as per federal guidelines (https://www.bls.gov/cps/rvcps03.pdf). We have utilized such information to uncover possible sources of social and economic race/ethnic disparities as implied by our framework. Given the limitations of the dataset, we have explicitly examined a subset of such social groups, and have articulated such decisions in the Method section.
Population characteristics	NA
Recruitment	NA
Ethics oversight	NA

Note that full information on the approval of the study protocol must also be provided in the manuscript.

Field-specific reporting

Please select the one below that is the best fit for your research. If you are not sure, read the appropriate sections before making your selection.

☐ Life sciences ☒ Behavioural & social sciences ☐ Ecological, evolutionary & environmental sciences

For a reference copy of the document with all sections, see nature.com/documents/nr-reporting-summary-flat.pdf

Behavioural & social sciences study design

All studies must disclose on these points even when the disclosure is negative.

Study description	We study the structure of skill dependencies in workplace skills, reframing classic topics in human capital. The study is quantitative, combining network analysis, data and regression analysis.
Research sample	We include brief descriptions of the four datasets used in this study to supplement in-depth description offered the Method section:<br>1. The Occupational Information Network (O*NET) includes survey records of job-oriented attributes and worker-oriented descriptors that pertains to a representative sample of individuals and establishment in the United States.<br>2. Current Population Survey (CPS) is a primary source of monthly labor force statistics. We have utilized CPS micro-data that contains surveyed demographic and work-related information, as well as aggregate-level estimates on occupational age information.<br>3. Occupational Employment and Wage Statistics (OEWS) from the Bureau of Labor Statistics (BLS). OEWS program produces employment and wage estimates annually for approximately 830 occupations. These estimates are available for the nation as a whole, for individual states, and for metropolitan and non-metropolitan areas; national occupational estimates for specific industries are also available. We have utilized national-level and metropolitan and non-metropolitan information.<br>4. Proprietary resume data offers information on over 70 million jobs organized in 20 million individual resumes between 2007 and 2020.
Sampling strategy	We used the full set of publication record of countries. No sampling strategy is used.
Data collection	No primary data is collected.
Timing	2005-2022
Data exclusions	We used the complete publication records of countries.
Non-participation	NA
Randomization	Our study doesn't involve random-control design.

Reporting for specific materials, systems and methods

We require information from authors about some types of materials, experimental systems and methods used in many studies. Here, indicate whether each material, system or method listed is relevant to your study. If you are not sure if a list item applies to your research, read the appropriate section before selecting a response.

Materials & experimental systems

n/a	Involved in the study	
☒	☐	Antibodies
☒	☐	Eukaryotic cell lines
☒	☐	Palaeontology and archaeology
☒	☐	Animals and other organisms
☒	☐	Clinical data
☒	☐	Dual use research of concern
☒	☐	Plants

Methods

n/a	Involved in the study	
☒	☐	ChIP-seq
☒	☐	Flow cytometry
☒	☐	MRI-based neuroimaging

Plants

Seed stocks	*Report on the source of all seed stocks or other plant material used. If applicable, state the seed stock centre and catalogue number. If plant specimens were collected from the field, describe the collection location, date and sampling procedures.*
Novel plant genotypes	*Describe the methods by which all novel plant genotypes were produced. This includes those generated by transgenic approaches, gene editing, chemical/radiation-based mutagenesis and hybridization. For transgenic lines, describe the transformation method, the number of independent lines analyzed and the generation upon which experiments were performed. For gene-edited lines, describe the editor used, the endogenous sequence targeted for editing, the targeting guide RNA sequence (if applicable) and how the editor was applied.*
Authentication	*Describe any authentication procedures for each seed stock used or novel genotype generated. Describe any experiments used to assess the effect of a mutation and, where applicable, how potential secondary effects (e.g. second site T-DNA insertions, mosiacism, off-target gene editing) were examined.*
